# Complete integration of carbene transfer chemistry into biosynthesis

**DOI:** 10.1038/s41586-023-06027-2

**Published:** 2023-05-03

**Authors:** Jing Huang, Andrew Quest, Pablo Cruz-Morales, Kai Deng, Jose Henrique Pereira, Devon Van Cura, Ramu Kakumanu, Edward E. K. Baidoo, Qingyun Dan, Yan Chen, Christopher J. Petzold, Trent R. Northen, Paul D. Adams, Douglas S. Clark, Emily P. Balskus, John F. Hartwig, Aindrila Mukhopadhyay, Jay D. Keasling

**Affiliations:** 1 Biological Systems and Engineering Division, Lawrence Berkeley National Laboratory, Berkeley, CA, USA.; 2 Joint BioEnergy Institute, Lawrence Berkeley National Laboratory, Emeryville, CA, USA.; 3 Department of Chemical and Biomolecular Engineering, University of California, Berkeley, CA, USA.; 4 Department of Chemistry, University of California, Berkeley, CA, USA.; 5 Chemical Sciences Division, Lawrence Berkeley National Laboratory, Berkeley, CA, USA.; 6 Novo Nordisk Foundation Center for Biosustainability, Technical University of Denmark, Kongens Lyngby, Denmark.; 7 Department of Biomaterials and Biomanufacturing, Sandia National Laboratories, Livermore, CA, USA.; 8 Molecular Biophysics and Integrated Bioimaging Division, Lawrence Berkeley National Laboratory, Berkeley, CA, USA.; 9 Department of Chemistry and Chemical Biology, Harvard University, Cambridge, MA, USA.; 10 Howard Hughes Medical Institute, Harvard University, Cambridge, MA, USA.; 11 Environmental Genomics and Systems Biology Division, Lawrence Berkeley National Laboratory, Berkeley, CA, USA.; 12 Department of Bioengineering, University of California, Berkeley, CA, USA.; 13 Synthetic Biochemistry Center, Institute for Synthetic Biology, Shenzhen Institutes for Advanced Technologies, Shenzhen, China.

## Abstract

Biosynthesis is an environmentally benign and renewable approach to produce a broad range of natural and, in some cases, new-to-nature products; however, biology lacks many of the reactions available to synthetic chemists, resulting in the scope of accessible products through biosynthesis to be narrower than those by synthetic chemistry. A prime example of such chemistry is carbene transfer reactions^1^. Although it was recently shown that carbene transfer reactions can be performed in a cell and used for biosynthesis^2,3^, carbene donors and unnatural cofactors needed to be added exogenously and transported into cells to effect the desired reactions, precluding cost-effective scale-up of the biosynthesis process with these reactions. Here, we report the access to a diazo ester carbene precursor by cellular metabolism and a microbial platform for introducing unnatural carbene transfer reactions into biosynthesis. The α-diazoester azaserine was produced by expressing a previously uncharacterized biosynthetic gene cluster in *Streptomyces albus*. The intracellularly produced azaserine was used as a carbene donor to cyclopropanate another intracellularly produced molecule, styrene. The reaction was catalyzed by engineered P450 mutants harboring a native cofactor with excellent diastereoselectivity and moderate yield. This work establishes a scalable, microbial platform for conducting intracellular abiological carbene transfer reactions to functionalize a range of natural and new-to-nature products and expands the scope of organic products that can be produced by cellular metabolism.

The repertoire of natural enzyme-catalyzed reactions lacks many of the common reactions of organic synthesis, including the carbene transfer reactions^4–7^. Although a few natural enzymatic transformations were proposed to involve carbene intermediates, it has been challenging to repurpose them for unnatural biosynthesis^8–12^. Recently, metalloenzymes have been engineered to catalyze many abiological carbene transfer reactions, such as the cyclopropanation of alkenes and insertions of carbene units into N–H and C–H bonds^13–15^. These enzymes exhibit impressive unnatural activity and selectivity (Fig. 1a). Integration of such unnatural carbene transfer reactions into microbial metabolism would open new possibilities to generate new-to-nature products in vivo through biosynthesis. Although abiological carbene transfer reactions catalyzed by small organometallic catalysts or artificial metalloenzymes have been interfaced with natural biosynthesis pathways to generate new products^2,3,16^, the diazo compound acting as carbene precursor and the metal cofactors were added to cells exogenously, and this addition limits the scale at which the reactions can be performed economically and the scope of substrates that can be imported into the cell. An intracellular biosynthetic route to the carbene precursor and engineered catalysts would mitigate these limitations and greatly expand the utility of enzymatic non-natural carbene transfer chemistry (Fig. 1b). Here, we show that the natural α-diazoester azaserine can be produced by *Streptomyces albus* engineered with a previously uncharacterized biosynthetic gene cluster and act as the carbene precursor for coupling with intracellularly produced styrene to generate unnatural amino acids containing a cyclopropyl group, which has wide applications in organic synthesis and occurs frequently in medicine development^17,18^, by a reaction catalyzed by an evolved cytochrome P450 mutant with its native cofactor. This work sets the stage for full integration of those carbene transfer reactions into biosynthesis and broadens the scope and structural diversity of chemicals that can be produced by cellular metabolism.

While there are many different carbene precursors in synthetic chemistry and methods to synthesize them in situ, most of these compounds are not made by living systems^7,19–21^. Diazo compounds, such as the widely used unnatural compound ethyl diazoacetate (EDA) ^22,23^, are common precursors to metal-carbene complexes. There are natural products that contain diazo groups^24^, and we considered that those natural molecules could serve as carbene precursors that could be produced biosynthetically (Fig. 2a). 6-Diazo-5-oxo-L-norleucine (DON) is a natural diazo compound, and its biosynthetic pathway has been elucidated recently^25^. DON and its derivatives have been used in carbene transfer reactions^26^. However, only N- and O-protected DON (the amino group and carboxyl group in DON were both modified with protecting groups) has been used for intermolecular reactions because the unprotected DON undergoes intramolecular insertion and fails to react intermolecularly under the conditions tested^26^. Although the biosynthetic pathways for some other natural diazo compounds have been reported, their molecular size, instability and/or reactivity may make them unsuitable to act as a carbene precursor^24,27,28^. However, the size and electronic properties of the natural product azaserine, initially identified as a drug for treating cancer^29,30^, are similar to those of EDA. Therefore, we reasoned that it could be a suitable reagent for intracellular carbene transfer reactions.

Azaserine was first tested for its ability to act as a carbene precursor for the cyclopropanation of styrene catalyzed by the artificial cofactor Ir(Me)MPIX (MPIX, mesoporphyrin IX) and hemin, both of which have been used previously to catalyze cyclopropanation^13,31^. The reaction products were analyzed by liquid chromatography-high resolution mass spectrometry (LC-HRMS) and compared with the independently synthesized authentic products. The cyclopropanation products were detected, indicating that azaserine can serve as a carbene precursor (Fig. 2b). While Na_2_S_2_O_4_ was essential for the activity of hemin (Extended Data Fig. 1b), consistent with previous studies^13^, it was not required for that of Ir(Me)MPIX (Fig. 2b and Extended Data Fig. 1a).

Having confirmed that azaserine can act as a carbene precursor, we searched for the biosynthetic pathway for it. There are two known organisms that produce azaserine naturally, *Streptomyces fragilis*^24^ and *Glycomyces harbinensis*^32^, and their genome sequences are available. Using comparative genomics, a gene cluster likely encoding for the biosynthesis of azaserine (*aza*), which is similar to those for some natural products containing nitrogen-nitrogen (N–N) bonds^27,33,34^, was identified in both strains (Extended Data Fig. 3a). To verify the prediction, given that the native producers are not genetically tractable, the gene cluster from *S. fragilis* was cloned and heterologously expressed in other hosts. Since the azaserine gene cluster is from *S. fragilis*, which is a prokaryote, the most commonly used model prokaryote *Escherichia coli* and a model streptomycete *S. albus*, which belongs to the same genus as *S. fragilis*, were selected. Because azaserine has antibiotic activity, expression of the pathway in a heterologous host would likely be toxic. To select a suitable host, the toxicity of azaserine toward *E. coli* and *S. albus* was tested. Consistent with previous studies^35^, the growth of *E. coli* BL21(DE3) was inhibited by 40 mg/L of azaserine added exogenously (Extended Data Fig. 2a). However, the growth of *S. albus* was not inhibited under the tested conditions (Extended Data Fig. 2b). Thus *S. albus* was selected as the host. The predicted gene cluster was cloned and integrated into the chromosome of *S. albus*. Proteomic analysis indicated 20 out of 23 proteins encoded in the cloned region were expressed (Extended Data Fig. 3b). Under the tested conditions, azaserine was biosynthesized by the engineered *S. albus* strain, providing functional validation of the predicted gene cluster (Fig. 2c). A detailed analysis of the enzymes encoded by the *aza* gene cluster and elucidation of the biosynthetic logic of azaserine production via feeding studies and in vitro biochemical characterization are described in a separate study (D.V.C., T.L.N., J.H., H.H., J.F.H., J.D.K., E.P.B. manuscript submitted). Under the optimized culture conditions, a titer of 103 mg/L was achieved at 48 h when the cells were grown in 1B medium (Fig. 2d). The titer of azaserine decreased with cultivation time in TSB medium, even after the cells were removed, possibly due to a reaction of azaserine with a component made by the cells when they were cultivated in TSB medium (azaserine was stable in fresh TSB medium and weak alkaline conditions^36^) (Extended Data Fig. 4a, b).

To incorporate an abiological carbene transfer reaction into biosynthesis, an appropriately engineered enzyme capable of using azaserine as the carbene precursor was required. Several P450s and their mutants (Supplementary Table 1) previously reported to catalyze cyclopropanation by carbene transfer^3,37,38^ were screened for activity toward the reaction of styrene with azaserine by using *E. coli* expressing their encoding genes. In addition, P450-T2, a cytochrome P450 recently identified from a metagenomic database originating from a hot-spring collection, was tested because it is a thermophilic enzyme that was reported to bind L-mimosine^39^, which is similar in structure to the hydrophilic azaserine. Given that the heterologous host *S. albus* is an aerobe and the reaction will be introduced into it, the screening process was conducted under aerobic conditions. In the initial screening, *E. coli* cells expressing the wild-type (WT) P450-T2 and a CYP119 mutant exhibited both significant activity and different diastereoselectivity compared to the control expressing the red fluorescence protein (RFP) (Fig. 3a). The control formed only a low level of products, possibly produced by the reaction catalyzed by the free heme inside the cell. Because the activity of P450-T2 was the highest among the enzymes tested in the initial screening, the activity of this enzyme was further confirmed by in vitro reaction with purified protein (Extended Data Fig. 5b). Although the diastereoselectivity (dr, the percentage of the major diastereomer versus the sum of all diastereomers in the product) from the reaction catalyzed by whole cells containing P450-T2 was 87.8%, it was 99.3% when the reaction was catalyzed by purified protein (Fig. 3c and Extended Data Fig. 5b). The lower diastereoselectivity achieved by whole cells was possibly due to the interference of free heme inside the cell, which can also catalyze the reaction with low diastereoselectivity (Extended Data Fig. 1b). CYP203A1 from *Rhodopseudomonas palustris*, a homolog of P450-T2^39^, and mutants of CYP203A1 with modification to the amino acid acting as the axial ligand to iron (C337H, C337S and C337T) were also tested as catalysts for the reaction of styrene with azaserine. The reactions catalyzed by *E. coli* cells expressing CYP203A1 WT and the C337H mutant formed more products than that from the reactions catalyzed by cells expressing RFP. However, the activity of CYP203A1 and its mutant was lower than that of P450-T2 WT (Extended Data Fig. 6). Thus, P450-T2 WT was selected for further engineering.

To guide the directed evolution, the crystal structure of P450-T2 was determined (Fig. 3b). Based on the structure and previous studies, several sites around the heme binding pocket and on specific loop regions were selected for site saturation mutagenesis and evaluation for the activity and selectivity of the resulting mutants. Turnover number (TON) per cell was used as the figure of merit instead of TON per catalyst because the former value includes any changes in protein production in the cell due to mutations. The first-round mutant (S239V) catalyzed the reaction to form 46 times the amount of product as was formed by the wild type enzyme (Fig. 3c). This site corresponds to the T268 residue in P450 BM3 (Extended Data Fig. 7), a site that has been reported to be important for increasing the activity of P450 BM3 toward carbene transfer reactions^13^. The third-round mutant (S239V, F338H) was about 5 times more active than the S239V mutant (Fig. 3c). The F338 position corresponds to F393 in P450 BM3 (Extended Data Fig. 7), whose mutation was reported to change the reduction potential of the heme and to affect its catalytic behavior^40^. After 5 rounds of evolution, the fifth-round mutant (P450-T2–5) catalyzed the reaction with high diastereoselectivity (dr >99%) and moderate yield (42.7%); this yield is 251 times higher than that from the reaction catalyzed by WT enzyme (Fig. 3c). Aside from catalyzing cyclopropanation of styrene with azaserine, P450-T2 WT and P450-T2–5 also catalyzed the insertion of the carbene unit from azaserine into the *sp*^*3*^ C–H bond of phthalan. The WT enzyme catalyzed the reaction with high diastereoselectivity (dr = 97.2%) (Fig. 3d). The activity of P450-T2–5 was about 220 times higher than that of WT; the diastereoselectivity was high (dr = 85.8%), although slightly lower than that of the WT. At this point, the highest yield for this reaction was 2.5%.

Having shown that the carbene precursor could be generated biosynthetically in bacteria and that it reacts with styrene in good yield when catalyzed by a mutant of P450-T2, we sought to biosynthetically generate the final component of the reaction, styrene. To do so, the biosynthetic pathway for styrene was reconstituted in the azaserine-producing *S. albus* strain by integrating the required genes into the genome (Fig. 4a). The biosynthesis of styrene had been established in *E. coli* and *Saccharomyces cerevisiae*^41–43^, but not in *Streptomyces*. The pathway consists of two enzymes to convert phenylalanine (Phe) to styrene: a phenylalanine ammonia lyase (PAL), the enzyme catalyzing the rate limiting step, and a ferulic acid decarboxylase (FDC). Although it was reported that a prenyltransferase was required to synthesize the cofactor of FDC^44^, we found that the additional prenyltransferase gene was not required for the activity of FDC, possibly because a homolog of this prenyltransferase (BioCyc database collection, accession ID: XNR_3566) is naturally expressed in *S. albus* (Extended Data Fig. 8a). Given that styrene is volatile, its production was conducted in sealed tubes possessing sufficient headspace for the oxygen needed to grow *S. albus*. Styrene was produced in both TSB medium and 1B medium. Feeding the culture with phenylalanine, which is the precursor to styrene, the titer reached a level of 20 mg/L (Fig. 4b and Extended Data Fig. 8b).

After engineering *S. albus* to produce both substrates for cyclopropanation of styrene with azaserine and evolving the cytochrome P450-T2 to catalyze the reaction with significantly increased activity, the evolved P450-T2–5 mutant was integrated into the genome of the azaserine-, styrene-producing *S. albus* strain to construct the final strain, which can biosynthetically produce all the components for the reaction. The final strain was then cultured to test if the unnatural cyclopropanes would be produced through the intended biosynthesis. After culturing the strain in 1B medium with 4 mM Phe for 72 h, the unnatural cyclopropanes were detected and confirmed by LC-HRMS and liquid chromatography tandem mass spectrometry (LC-MS/MS) (Fig. 4c and Extended Data Fig. 9a). The results of a time course show that the titer does not increase after 72 h under the current conditions (Extended Data Fig. 9b). Having observed this product, different media were tested, and one more copy of the P450-T2–5 gene was integrated into the chromosome to increase the titer. Under these final conditions, 222 μg/L of the cyclopropane product formed with 95% dr (Fig. 4d). Thus, the engineered *S. albus* was able to biosynthesize all reaction components for an abiological carbene transfer reaction and produce unnatural products without the need for exogenously added carbene precursors and catalysts. Currently, the conversion efficiency for the integrated carbene transfer reaction and the final product titer are not high, but sufficient for proof of concept. Although we were able to increase the activity of P450-T2 by 251-fold through mutations, we believe the major limitation is still the activity of the P450-T2–5 mutant, which was engineered to catalyze an abiological reaction of two non-native substrates by a non-natural catalytic mechanism.

This study established a platform for introducing abiological carbene transfer reactions into microbial metabolism by producing the carbene precursor azaserine and showed that abiological carbene transfer reactions can be fully incorporated into biosynthesis. With all components in the carbene transfer reaction made by the microbe, the system will be easier and less expensive to scale industrially than if one or more components need to be added to the system. This strategy is not limited to azaserine. There are other natural and potentially unnatural carbene precursors that could be synthesized in vivo. In addition, many natural and unnatural products, such as limonene, carvone and indoles^3,45^, have been reported to be substrates for carbene chemistry catalyzed by engineered hemoproteins, some of which could be generated biosynthetically as the carbene acceptor, thereby creating new therapeutics, scaffolds for drug discovery, and precursors to materials. Furthermore, hemoproteins have been engineered to catalyze carbene insertions into a variety of chemical bonds (C–H, N–H and S–H) ^14,15^ that are common in natural and unnatural compounds. It is also possible that the carbene acceptor can be added exogenously to the medium to react with the azaserine produced in vivo, and the reaction can be catalyzed by the engineered enzymes inside the microbes. In this case, the product could be harvested directly or serve as an intermediate for further enzymatic transformations inside or outside the cell. Finally, it may be possible to incorporate azaserine into a protein as a noncanonical amino acid and then perform the carbene transfer reaction on the resulting protein or to perform the carbene transfer reaction of azaserine prior to its incorporation into a protein, either of which would result in a protein with unnatural amino acids, further broadening the applicable scope of this platform.

*Streptomyces* was used as the host in this study, and this genus produces many widely-used antibiotics and other valuable complex organic chemicals. Modification and diversification of those molecules would generate unnatural medicinal candidates for exploration. Such unnatural biosynthesis is not limited to *Streptomyces* or even bacteria, as one could imagine introducing those carbene transfer reactions into other widely-used industrial microorganisms, such as *Corynebacterium* or *Saccharomyces.* With the increasing number of reactions previously possible only through synthetic chemistry being executed by engineered enzymes, the integration of these enzymes into microbes and their reactions into biosynthesis should create new opportunities for bio-manufacturing.

## Methods

### Chemical standards

All commercial (ordered from MilliporeSigma) and chemically synthesized standards have a purity of 95% or higher. Methods for chemical standard synthesis are available in Supplementary Information.

### In vitro reaction using purified proteins or metal cofactors

In a 2-mL brown GC vial, a specific volume of M9-N buffer was transferred to the reaction vial followed by the addition of Na_2_S_2_O_4_ (0.5 M stock in M9-N buffer), azaserine (100 mM stock in M9-N buffer) and styrene (100 mM stock in ethanol). Catalysts, metal cofactor (4 mM stock in DMSO) or purified proteins (100 μM stock in M9-N buffer), were added, resulting in a specific concentration and a final reaction volume of 300 μL. The vials were sealed with caps and shaken at 200 rpm and 22 °C for 18 h. The reaction was quenched by adding 300 μL methanol, vortexed at a speed of 3000/min for 10 min, and stored at −80 °C before analysis. The samples were thawed, briefly vortexed, and centrifuged at 20,000 xg for 8 min. The supernatant was filtered with a 3 kDa centrifugal filter and analyzed by liquid chromatography-mass spectrometry (LC-MS). To make the M9-N reaction buffer, salts (47.7 mM Na_2_HPO_4_, 22.0 mM KH_2_PO_4_, 8.6 mM NaCl) were dissolved in 1 L ddH_2_O and autoclaved to give a medium with pH ~7.4. Solutions of MgSO_4_ (1 M), CaCl_2_ (1 M) and glucose (20 w/v%) were added to give a final concentration of 2.0 mM MgSO_4_, 0.1 mM CaCl_2_, and 0.4 w/v% glucose.

### Reaction using *E. coli* whole cells

Plasmids harboring the genes encoding the desired enzymes were transformed into BL21(DE3) competent cells (NEB). Selection plates were incubated at 30 °C overnight. Three colonies for each strain were inoculated into 3 mL Terrific Broth (TB) medium containing 50 μg/mL kanamycin and grown at 30 °C with shaking (200 rpm) overnight. In a 50-mL glass tube, 50 μL overnight culture was inoculated into 5 mL fresh TB medium containing 50 μg/mL kanamycin and grown at 37 °C with shaking at 200 rpm. When the OD_600_ (optical density at 600 nm) reached approximately 0.6, the culture was cooled to 22 °C with shaking (200 rpm) for 30 min. IPTG and 5-aminolevulinic acid were added to final concentrations of 0.2 mM and 0.5 mM, respectively. The cultures were grown at 22 °C and 200 rpm shaking for 18 h. After measuring the OD_600_, 9 OD_600_ units of cells were collected by centrifugation at 10,000 xg for 1 min and resuspended in 270 μL M9-N buffer. The cell suspension was transferred to a 2-mL brown GC vial. Azaserine (100 mM stock in M9-N buffer) and styrene (100 mM stock in ethanol) or phthalan (200 mM stock in ethanol) were added resulting in specific final concentrations. The final reaction volume was 300 μL, and the final cell concentration was 30 OD_600_. The vials were sealed with screw caps and shaken at 200 rpm and 22 °C for 18 h. The reaction was quenched by adding 300 μL methanol, vortexed at speed of 3000/min for 10 min, and stored at −80 °C prior to analysis. Before analysis, the samples were thawed, briefly vortexed, and centrifuged at 20,000 xg for 8 min. The supernatant was filtered through a 3 kDa centrifugal filter and analyzed by LC-MS.

### Azaserine toxicity

For *E. coli*, 3 single colonies were inoculated into Luria-Bertani (LB) medium and grown overnight at 30 °C with shaking at 200 rpm. The culture was diluted 1:100 into 5 mL of fresh LB medium with different concentrations of azaserine in 50-mL glass tubes and grown at 30 °C with shaking at 200 rpm. The cell density was analyzed at 24 h and 48 h.

For *S. albus*, 20 μL spores were inoculated into 50-mL glass tubes containing 3 mL Tryptic Soy Broth (TSB) medium with 4–6 glass beads of ~5 mm diameter and grown at 30 °C and 200 rpm shaking for 48 h. Then, the culture was diluted 1:50 into 5 mL of fresh TSB medium with different concentrations of azaserine in 50-mL glass tubes containing 4–6 glass beads. The cells were grown at 30 °C and 200 rpm shaking. At 24 and 48 h after inoculation, 1 mL samples of the cultures were collected and stored at −80 °C. Biomass was quantified using the methylene blue method^46^.

### Identification of the biosynthetic gene cluster for azaserine

Comparative genomics was used to identify the azaserine biosynthetic gene cluster (*aza* BGC). A database was assembled with the genomes of 7,763 bacterial strains retrieved from the GenBank and 1,913 BGC entries from the MiBIG database. This database included the genomes of *Streptomyces fragilis* NBRC 12862 (a.k.a. strain PD 04926) and *Glycomyces harbinensis* CGMCC 4.3516, as well as the biosynthetic gene clusters for the triacsins from *Kitasatospora aureofaciens* (MIBIG entry: BGC0001983), s56-p1 from *Streptomyces* sp. SoC090715LN (BGC0001764), pyrazomycin from *Streptomyces candidus* (BGC0001985), and actinopyridazinone from *Streptomyces* sp. MSD090630SC-05. All these DNA sequences were annotated using RAST^47^, and their amino acid sequences and tabular feature files were used as input for the CORASON pipeline^48^. The pipeline was modified to improve the command line interface, allow processing of large genome datasets, eliminate dependencies, increase the speed of the analysis and improve phylogenetic analysis by incorporation of maximum likelihood phylogenetic calculations with model selection using IQtree^49^. The modified code is freely available at: https://github.com/WeMakeMolecules/myCORASON. The genome mining was performed using tri17 (protein ID QCT05750.1) and tri28 (protein ID QCT05761.1) as queries, with a cutoff e-value of 1E^−12^ and a bit score cutoff of 200. Orthologous BGCs were called only if homologs of Tri28 or Tri17 were found within a gene neighborhood that included at least one more ortholog from the triacsin BGCs. The gene neighborhoods were defined as 15 genes upstream and 15 genes downstream of the tri17 or tri 28 homologs.

### Protein expression and purification

For protein expression, a single colony of *E. coli* BL21(DE3) harboring a plasmid encoding P450-T2 WT or mutant was grown overnight in 30 mL of TB medium with 50 μg/mL kanamycin at 37 °C and 200 rpm shaking. The culture was used to seed (1:100 dilution) 500 mL TB medium containing 50 μg/mL kanamycin in a 2-L non-baffled flask. The cells were grown at 37 °C and 200 rpm shaking to OD_600_ = 0.7 before moving to 22 °C and 200 rpm shaking. After 30 min, IPTG and 5-aminolevulinic acid were added to final concentrations of 0.25 mM and 0.5 mM, respectively. The cultures were grown at 22 °C and 200 rpm shaking for 18–20 h before collecting the cells by centrifugation at 4,000 xg for 30 min. The pellets were weighed (~ 5 g) and stored at −80 °C.

For protein purification, cells collected above were resuspended in 50 mL lysis buffer (25 mM Tris-HCl, pH 8.0, 100 mM NaCl, 30 mM imidazole, 0.1 mg/mL lysozyme, 0.05 mg/mL DNase, 1 mM MgCl_2_ and 5 mg hemin) and mixed by vortexing for 30 min. The lysate soluble fraction was obtained by further sonication, high speed centrifugation at 37,000 xg for 30 min, and filtering through a 0.45-μm filter. The lysate was then subjected to a 5-mL Ni-NTA column, washed with 10 column volumes of Ni-NTA wash buffer (25 mM Tris-HCl, pH 8.0, 100 mM NaCl, 10 mM imidazole) at 3 mL/min flow rate, and eluted with an imidazole gradient (10 mM – 300 mM in 20 min). The eluted fractions were assessed by SDS-PAGE, and fractions with the target protein were further subjected to a 5-mL Q-trap column. The protein was washed with 5 column volumes of Q-trap wash buffer (25 mM Tris-HCl, pH 8.0) at 3 mL/min flow rate, and eluted with a NaCl gradient (0 mM – 500 mM in 20 min). Elution fractions were assessed by SDS-PAGE, and selected fractions were dialyzed (1:100 v:v) at 4 °C overnight in storage buffer (25 mM Tris-HCl, pH 8.0, 200 mM NaCl, 10% glycerol). Finally, the protein concentration was determined by NanoDrop, followed by liquid N_2_ flash cooling and −80 °C storage.

### Crystallization, X-Ray data collection and structure determination

The P450-T2 was concentrated to 10 mg/mL. Crystals of P450-T2 were found in 0.2 M ammonium sulfate and 20 % PEG 3,350. The crystal of P450-T2 was placed in a reservoir solution containing 20% (v/v) glycerol, then flash-cooled in liquid nitrogen. The X-ray diffraction data set for P450-T2 was collected at the Berkeley Center for Structural Biology beamline 8.2.1 at the Advanced Light Source at Lawrence Berkeley National Laboratory. The diffraction data was collected using a wavelength of 1.000 Å and the temperature of 100 K. The data set was processed using the program Xia2^50^. The crystal structure of P450-T2 was solved by molecular replacement with the program PHASER^51^ using a model generated by AlphaFold^52^. The atomic positions obtained from the molecular replacement were used to initiate refinement with the Phenix suite^53^. Structure refinement was performed using the phenix.refine program^54^. Manual rebuilding was performed with COOT^55^. Root-mean-square deviations from ideal geometries for bond lengths, bond angles and dihedral angles were calculated with Phenix^54^. The stereochemical quality of the final model of P450-T2 was assessed by the program MOLPROBITY^56^. The Ramachandra plot showed 97.4 % of residues present in favored regions, 2.6 % of residues in allowed regions and no residues are outliers. Summary of crystal parameters, data collection, and refinement statistics can be found in Supplementary Table S3.

### Site-saturation mutagenesis

Plasmid pBbE7k^57^ was used as the vector for cloning and expression in *E. coli* and digested by NdeI and BamHI. 19 individual PCRs (20 μL for each) were performed for each screened residue site with primers containing specific mutations for saturation mutagenesis. 19 PCRs were divided into 3 groups, each group with 6–7 PCR products. Aliquots of 8 μL from individual PCR reactions from the same group was taken and mixed together, resulting three PCR product mixtures. The mixtures were added with 5 μL of DpnI and incubated at 37 °C for 3 h. All the fragments were purified by gel electrophoresis. Purified DNA fragments were ligated by Gibson Assembly. 2 μL of the assembly products were added to 50 μL BL21(DE3) competent cells (NEB) for each group. 24 colonies were picked from the transformation plate for each group and patched to 2 parallel LB agar plates containing 50 μg/mL kanamycin. One plate was sent for sequencing and the other was kept for inoculation.

### Strain construction

The *S. albus* J1074 strains harboring the exogenous genes were constructed following a protocol described previously^58^ with minor modifications as below. When spectinomycin resistance was used as the selection, 100 μg/mL spectinomycin was used for *E. coli* selection and 200 μg/mL for *S. albus* selection. Single *S. albus* colonies from conjugation plates were inoculated into 2 mL TSB medium containing 200 μg/mL spectinomycin (or 50 μg/mL apramycin) and 25 μg/mL nalidixic acid in 24 well plates (~4 glass beads with 5 mm diameter were added to each well), and the plates were incubated at 30 °C and 200 rpm shaking for 3 days. Candidates with the correct integration were screened and verified by PCR or whole cell reaction (using cells as catalysts to convert phenylalanine to styrene for verifying strains constructed to produce styrene). For the whole cell conversion, 1 mL culture was centrifuged at 20,000 xg for 2 min. The supernatant was discarded, and the pellets were washed with 500 μL M9-N buffer, resuspended in 300 μL M9-N buffer, and transferred to 2-mL GC vials. Phenylalanine was added to a final concentration of 5 mM (15 μL 0.1 M phenylalanine stock), the vials were capped and shaken at 200 rpm and 30 °C for 24 h. Then 600 μL hexane containing 80 μg/mL beta-pinene was added. The vials were vortexed at a speed of 3000/min for 5 min. Samples were transferred to a 1.7-mL tube and centrifuged at 20,000 xg for 8 min. The organic layer (200 μL) was transferred to a vial for styrene analysis by gas chromatography-mass spectrometry (GC-MS). Strains with positive PCR results or styrene production were stored at −80 °C.

### Media and *S. albus* cultivation

For azaserine production, spores (30 μL) of *S. albus* J1074 with the azaserine gene cluster integrated into genome were inoculated into 4 ml TSB medium in 50-mL glass tubes with approximately 10 glass beads of 5 mm diameter. The cells were grown at 30 °C and 200 rpm shaking for 48 h. Then the cells were diluted 1:50 into 30 mL various media in 250-mL non-baffled flasks with 8-layer cotton gauze caps and grown at 28 °C with 200 rpm shaking. At several time points, samples of 500 μL were removed and stored at −80 °C. For analysis of azaserine titers, the samples were thawed, 500 μL of methanol was added, and the mixture was vortexed at 3000/min for 10 min and then centrifuged at 20,000 xg for 8 min. The supernatant was filtered through a 3-kDa centrifugal filter and analyzed by LC-MS.

For styrene production, 60 μL of mycelium stock (stored at −80 °C) were inoculated into 3 mL (1:50) TSB medium with 200 μg/mL spectinomycin and 25 μg/mL nalidixic acid in 50-mL glass tubes containing approximately 10 glass beads of 5 mm diameter. The cultures were grown at 30 °C and 200 rpm shaking for 72 h. Then the cells were diluted 1:50 into 5 mL fresh media (containing 200 μg/mL spectinomycin and 25 μg/mL nalidixic acid) with various concentrations of phenylalanine in 50-mL conical tubes containing 6 glass beads of 5 mm diameter, which were sealed, incubated at 28 °C with 200 rpm shaking. At the specific time points, 500 μL samples were taken and extracted with 500 μL hexane containing 80 ng/μL beta-pinene, vortexed at 3000/min for 10 min, and centrifuged at 20,000 xg for 8 min. The organic layer (200 μL) was transferred to a vial for GC-MS analysis.

For generation of the product by styrene and azaserine cross-coupling, the cells were cultured as described above for styrene production, except that 0.5 mM 5-aminolevulinic acid was added to the growth medium. The samples were analyzed using LC-MS as described above for azaserine. MYI medium (1 L) consisted of 4 g yeast extract, 10 g malt extract, 4 g glucose, add ddH_2_O to 1 L, autoclave at 121 °C, 20 min. 1B medium (1 L) (modified from an expired patent^59^): glucose 10 g, soybean meal 10 g, casein lysate 5 g, yeast extract 5 g, sodium chloride 0.5 g, ammonium nitrate 2.5 g, add ddH_2_O water to 1 L, adjust pH to 7.5. 1 g of CaCO_3_ was added to 1 L of the medium prior to autoclaving at 121 °C for 20 min.

### Plasmid construction

All plasmids (see Supplementary Table 2) were constructed using Gibson Assembly or restriction enzyme cloning. The DNA products were transformed into *E. coli* DH5α competent cells, plated onto LB agar plates with corresponding antibiotics (100 μg/mL spectinomycin, 50 μg/mL kanamycin or 50 μg/mL apramycin). For cloning fragments larger than 15 kb, the transformed cells were incubated at 30 °C, whether on agar plates or the subsequent liquid cultures. All other transformed cells were incubated at 37 °C. Mini-prepped plasmids were digested with restriction enzymes. Candidates with correct digestion patterns were sent for sequencing. To clone the azaserine gene cluster, *Streptomyces fragilis* genomic DNA was used as template for amplification of the gene fragment. All other exogenous genes to be expressed in *S. albus* were codon optimized for *S. albus* and ordered from IDT or GeneScript. The conjugation plasmids used for integrating genes into *S. albus* contained the p15A origin.

### GC-MS and LC-MS analyses

Styrene was analyzed using an Agilent Technologies GC-MS system equipped with an HP-5 column (Agilent, USA). The temperature in the GC oven was increased from 50 °C to 110 °C at a rate of 10 °C/min. The solvent delay was set at 3 min. Single ions (m/z 78, 93, 104, 121) were detected. The other products were analyzed using high-performance liquid chromatography-mass spectrometry (HPLC–MS) using an Agilent 1260 infinity ii with mass selective detector iQ (MSD iQ) or an Agilent Technologies 6520 with quadrupole time-of-flight mass spectrometer (Q-TOF-MS). The HPLC autosampler and column compartment were maintained at 6 °C and 25 °C, respectively. An Agilent Technologies ZORBAX Eclipse Plus C18 (3.5 μm, 4.6 mm x 150 mm) column was used for azaserine analysis. A MilliporeSigma Astec CYCLOBOND I 2000 HP-RSP Chiral HPLC (5 μm, 25 cm x 4.6 mm) column was used to analyze the reaction products of azaserine with styrene (aza-sty) or with phthalan (aza-phtha). For aza-sty analysis, the mobile phase was composed of 10 mM ammonium formate in water with 50 μL formic acid added per liter (solvent A) and 0.1% (v/v) formic acid in acetonitrile (solvent B). For aza-sty separation and analysis, the column was eluted with 14% B from 0–32 min at a flow rate of 0.35 mL/min for 0–23 min, increased to 0.8 mL/min over 0.2 min, and held at 0.8 mL/min for 23.2–32 min and then flushed for the next analysis. For aza-phtha analysis, the column was eluted with a linearly increasing concentration of B from 13% to 13.7% over 40 min with a flow rate of 0.2 mL/min and then flushed for the next analysis.

Electrospray ionization was performed in the positive ion mode with a capillary voltage of 3,500 V, for [M+H]^+^ ions. Drying gas temperature and flow rate were maintained at 340 °C and 11 L/min, respectively, and the nebulizer gas was set to 30 lb/in^2^. Fragmentor, skimmer, and OCT 1 RF Vpp voltages were set to 120 V, 60 V, and 250 V, respectively. Targeted MS/MS was performed with a 250.107384 precursor m/z ion selection in the quadrupole at the analyte’s retention time using a delta retention time window of 2.5 minutes, an isolation width of ~1.3 m/z, and a collision energy of 20 eV for collision-induced dissociation. The resulting fragment ions were analyzed by the TOF mass analyzer.

### Proteomics method

Azaserine-producing *S. albus* strains were grown in TSB medium. Following 24 h of growth at 30 °C, samples were collected for proteomic analysis. Protein was extracted and tryptic peptides were prepared following established proteomic sample preparation procedures. Briefly, cell pellets were resuspended in Qiagen P2 Lysis Buffer (Qiagen, Hilden, Germany, Cat.#19052) to promote cell lysis. Proteins were precipitated by addition of 1 mM NaCl and 4 volumes of acetone, followed by two additional washes with 80% acetone in water. The recovered protein pellet was homogenized in 100 mM ammonium bicarbonate in 20% methanol. Protein concentration was determined by the DC protein assay (BioRad, Hercules, CA). Protein reduction was accomplished using 5 mM tris 2-(carboxyethyl)phosphine (TCEP) for 30 min at room temperature, and alkylation was performed with 10 mM iodoacetamide (IAM; final concentration) for 30 min at room temperature in the dark. Overnight digestion with trypsin was accomplished with a 1:50 trypsin:total protein ratio. The resulting peptide samples were analyzed on an Agilent 1290 UHPLC system coupled to a Thermo Scientific Orbitrap Exploris 480 mass spectrometer for discovery proteomics^60^. Briefly, peptide samples were loaded onto an Ascentis^®^ ES-C18 Column (Sigma–Aldrich, St. Louis, MO) and separated with a 10-minute LC gradient (10% Buffer A (98% H2O, 2% ACN) – 35% Buffer B (2% H2O, 98% ACN)). Eluting peptides were introduced to the mass spectrometer operating in positive-ion mode and were measured in data-independent acquisition (DIA) mode with a duty cycle of 3 survey scans from m/z 380 to m/z 985 and 45 MS2 scans with precursor isolation width of 13.5 m/z to cover the mass range. DIA raw data files were analyzed by an integrated software suite DIA-NN^61^. The database used in the DIA-NN search (library-free mode) is the latest Uniprot *S. albus* proteome FASTA sequence plus the protein sequences of the identified azaserine gene cluster and common proteomic contaminants. DIA-NN determines mass tolerances automatically based on first pass analysis of the samples with automated determination of optimal mass accuracies. The retention time extraction window was determined individually for all MS runs analyzed via the automated optimization procedure implemented in DIA-NN. Protein inference was enabled, and the quantification strategy was set to Robust LC = High Accuracy. Output main DIA-NN reports were filtered with a global FDR = 0.01 on both the precursor level and protein group level. The Top3 method, which is the average MS signal response of the three most intense tryptic peptides of each identified proteins, was used to plot the quantity of the azaserine gene cluster proteins in the samples^62^.

## Extended Data

**Extended Data Fig. 1 |:**
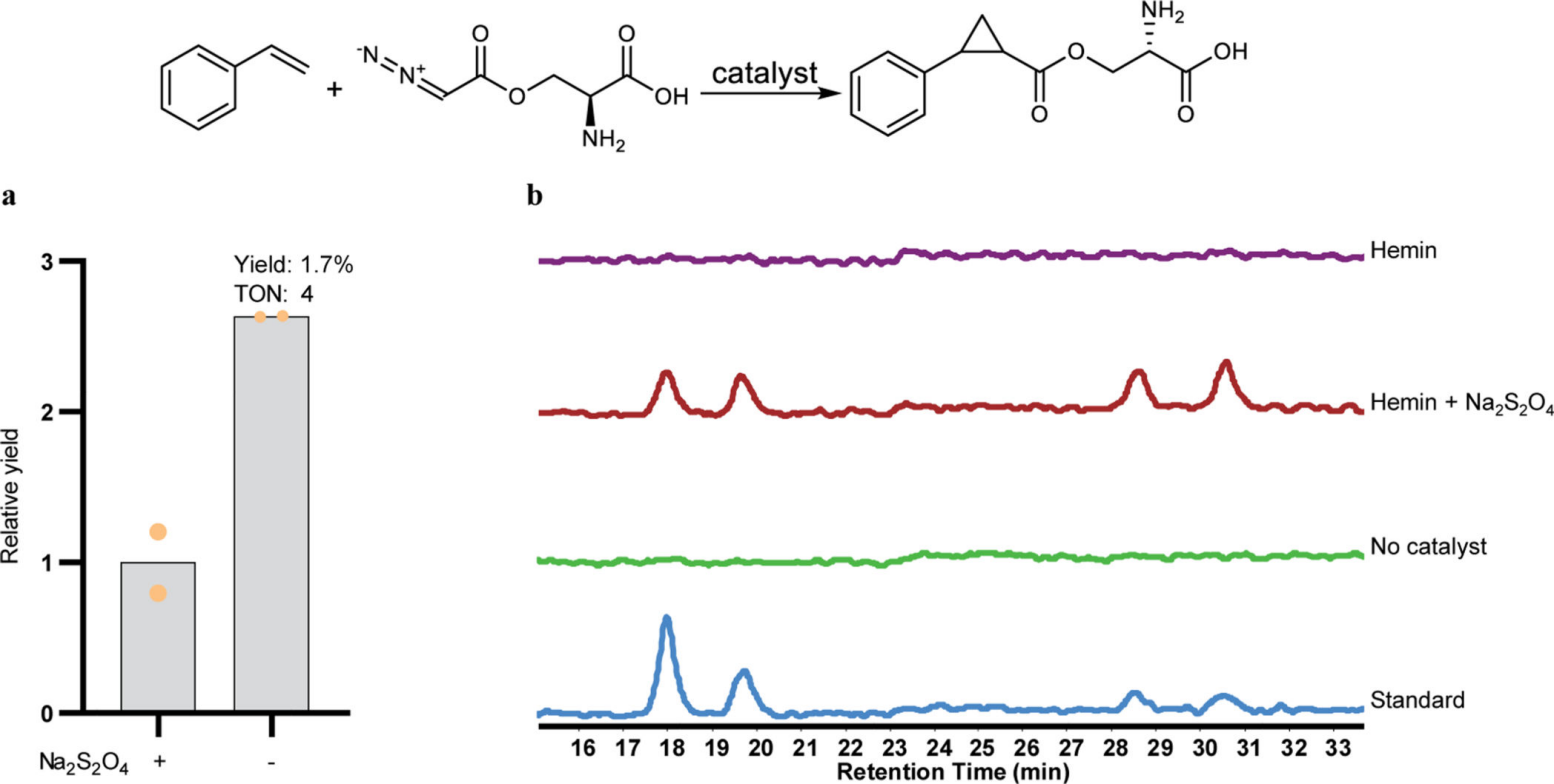
Effect of Na_2_S_2_O_4_ on the activity of hemin and Ir(Me)MPIX for the reaction of styrene with azaserine. **a,** Addition of Na_2_S_2_O_4_ decreased the reaction yield when using Ir(Me)MPIX as catalyst. TON, turn-over number. Reaction conditions are described in Fig. 2 legend. Data are mean value for 2 reaction replicates. **b,** Na_2_S_2_O_4_ is necessary for the activity of hemin toward the reaction. EIC ([M+H]^+^, *m*/*z* 250.1074) for target products. The traces are representative of two reaction replicates. The reaction contained 5 mM styrene, 5 mM azaserine, 10 μM hemin or no catalyst, 0 or 10 mM Na_2_S_2_O_4_, 5 vol% ethanol, and M9-N buffer and was conducted at 22 °C under aerobic conditions for 18 h. Standard, chemically synthesized authentic standard mixture of the four diastereomers.

**Extended Data Fig. 2 |:**
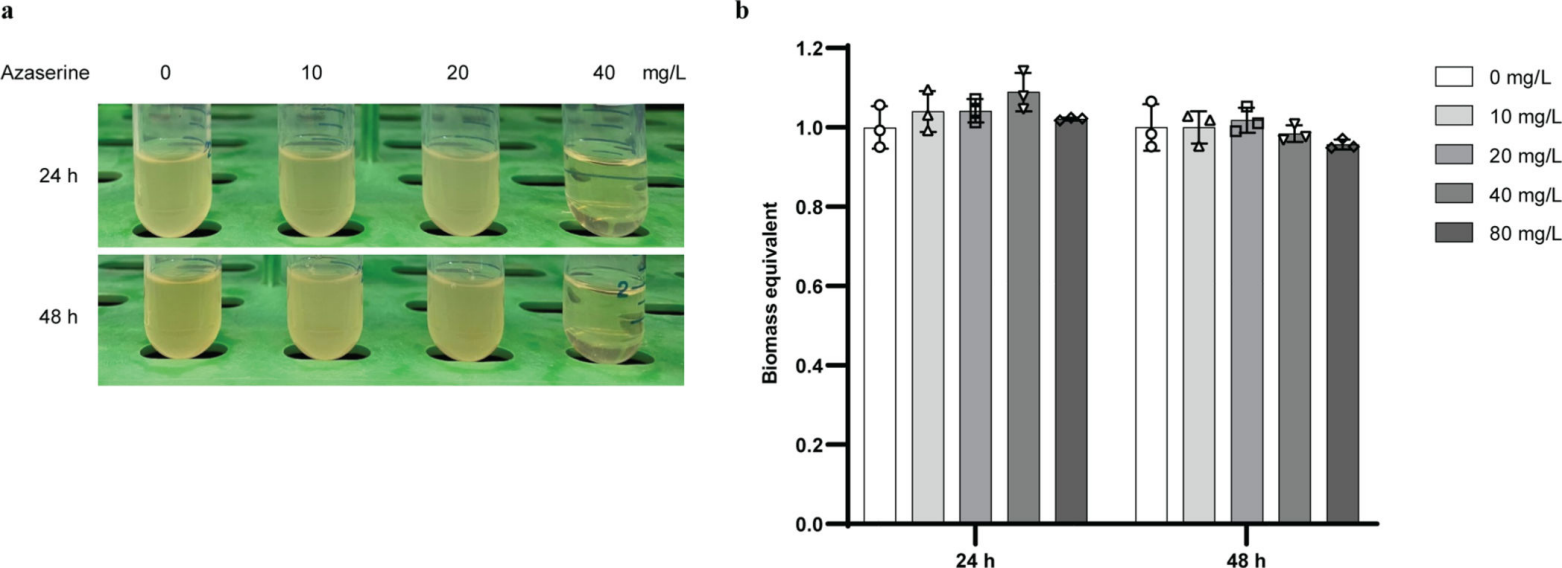
Azaserine toxicity on *E. coli* and *S. albus*. **a,** Azaserine is toxic to *E. coli* BL21(DE3). **b,** Azaserine does not affect the growth of *S. albus* under tested concentrations. Biomass was normalized to that of culture without addition of azaserine. Data are mean ± s.d.; *n* = 3 biological replicates.

**Extended Data Fig. 3 |:**
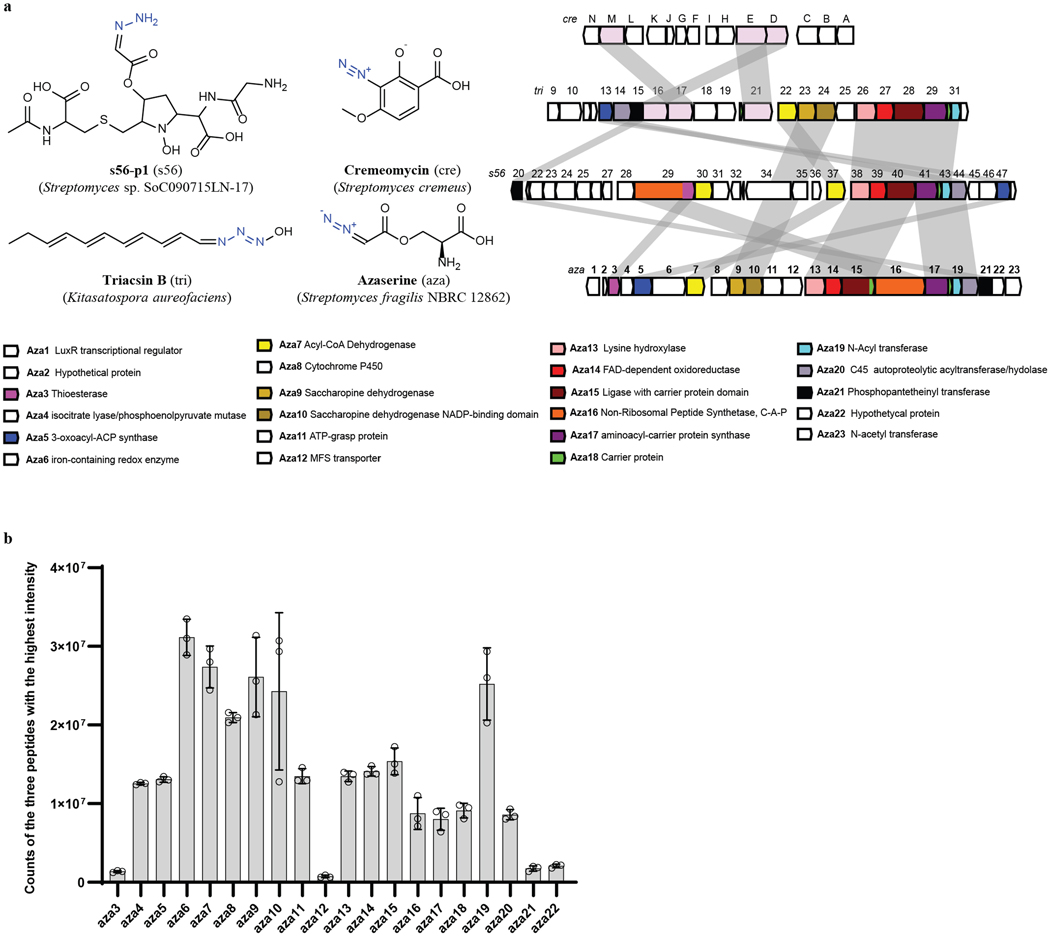
Expression of azaserine gene cluster from *S. fragilis* in *S. albus*. **a,** Bioinformatic annotation of the azaserine gene cluster and comparison with the biosynthetic gene clusters for some natural N–N bond-containing compounds. **b,** Proteomic analysis of the azaserine gene cluster when expressed in *S. albus*. Data are mean ± s.d.; *n* = 3 biological replicates.

**Extended Data Fig. 4 |:**
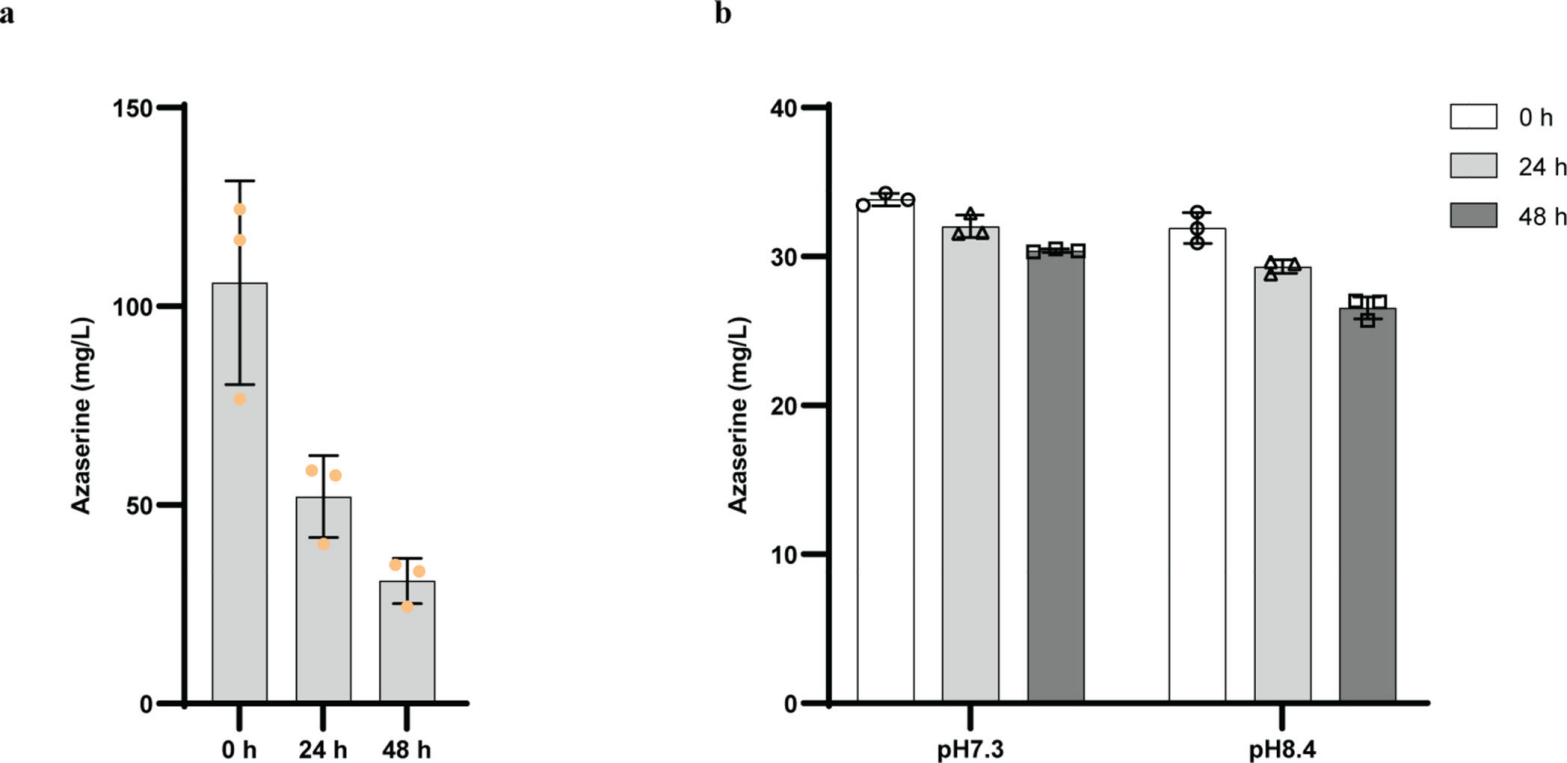
Differences in azaserine degradation in various media. **a,** Azaserine titer continues to decrease after removal of *S. albus* cells. The azaserine-producing *S. albus* was grown in TSB medium. After 24 h, the cells were removed from culture broth using a 0.22-μm sterile filter, the filtrate was incubated at 30 ℃ (labeled as 0 h), and the azaserine concentration was monitored at different time points. **b,** Azaserine is stable in fresh TSB medium of normal pH 7.3 or adjusted pH 8.4. Equal volume of azaserine stock was added to a final concentration of about 35 mg/L at 0 h. Data are mean ± s.d.; *n* = 3 biological (**a**) or technical (**b**) replicates.

**Extended Data Fig. 5 |:**
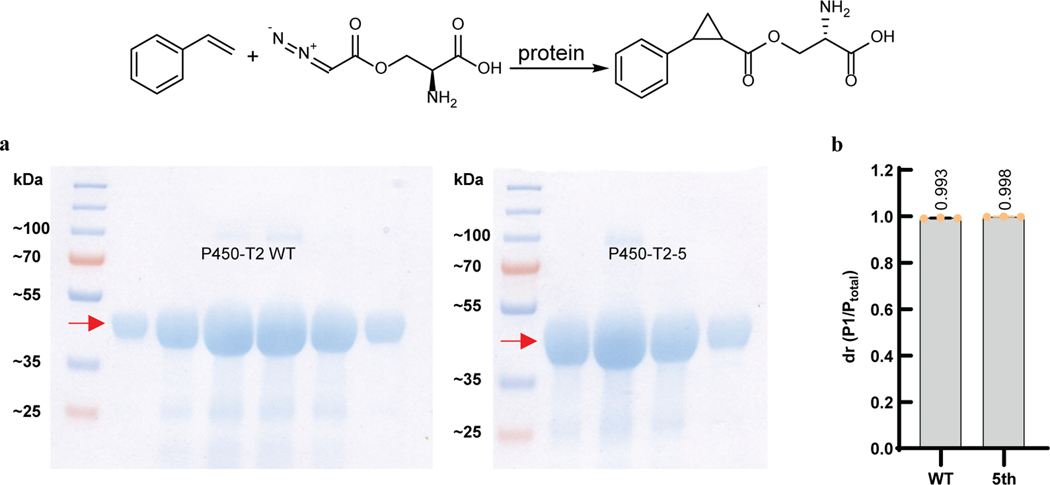
Purified P450-T2 WT and mutant catalyzing the reaction of styrene with azaserine in vitro. **a,** Coomassie Blue stained SDS-PAGE gel of purified protein P450-T2 WT (left) and P450-T2–5 mutant (right). Each lane is a sample from fractions collected during ion exchange purification. **b,** Purified P450-T2 WT and P450-T2–5 mutant proteins catalyze the reaction in vitro with high diastereoselectivity. 5th, P450-T2–5 mutant. Reaction conditions: 5 mM styrene, 5 mM azaserine, 10 μM enzyme, 10 mM Na_2_S_2_O_4_, 5 vol% ethanol, M9-N buffer, conducted at 22 °C under aerobic condition for 18 h. P_total_, sum area for all diastereomers. Grey bars indicate the Dr. Data are mean ± s.d.; *n* = 3 reaction replicates.

**Extended Data Fig. 6 |:**
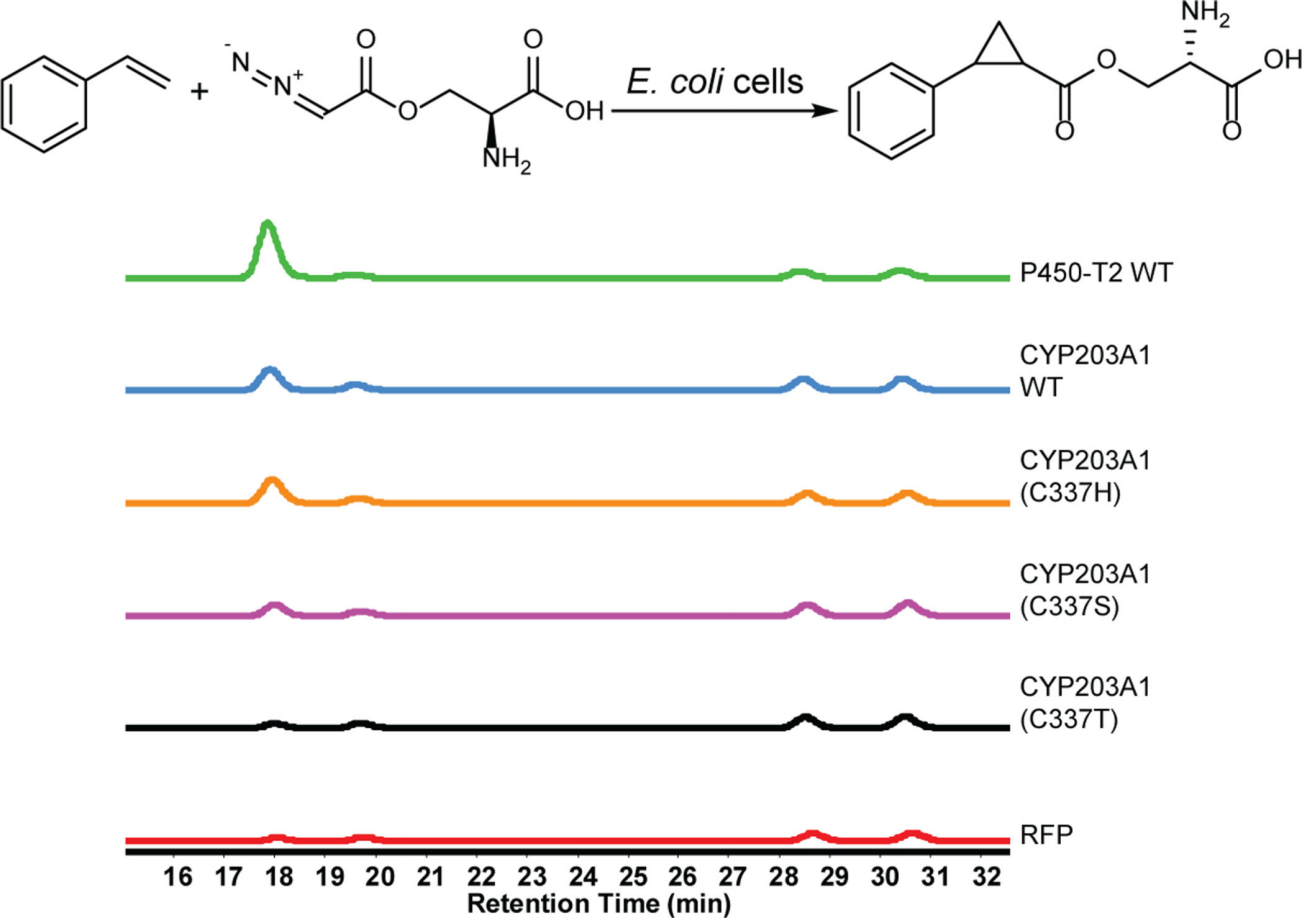
CYP203A1 WT and axial ligand mutants for the reaction of styrene with azaserine. EIC ([M+H]^+^, *m*/*z* 250.1074) for target products. Representative traces for two repeated experiments. The reactions contained 5 mM styrene, 5 mM azaserine, *E. coli* cells with concentration of 30 OD_600_ as catalyst, 5 vol% ethanol, and M9-N buffer and were conducted at 22 °C under aerobic conditions for 18 h.

**Extended Data Fig. 7 |:**
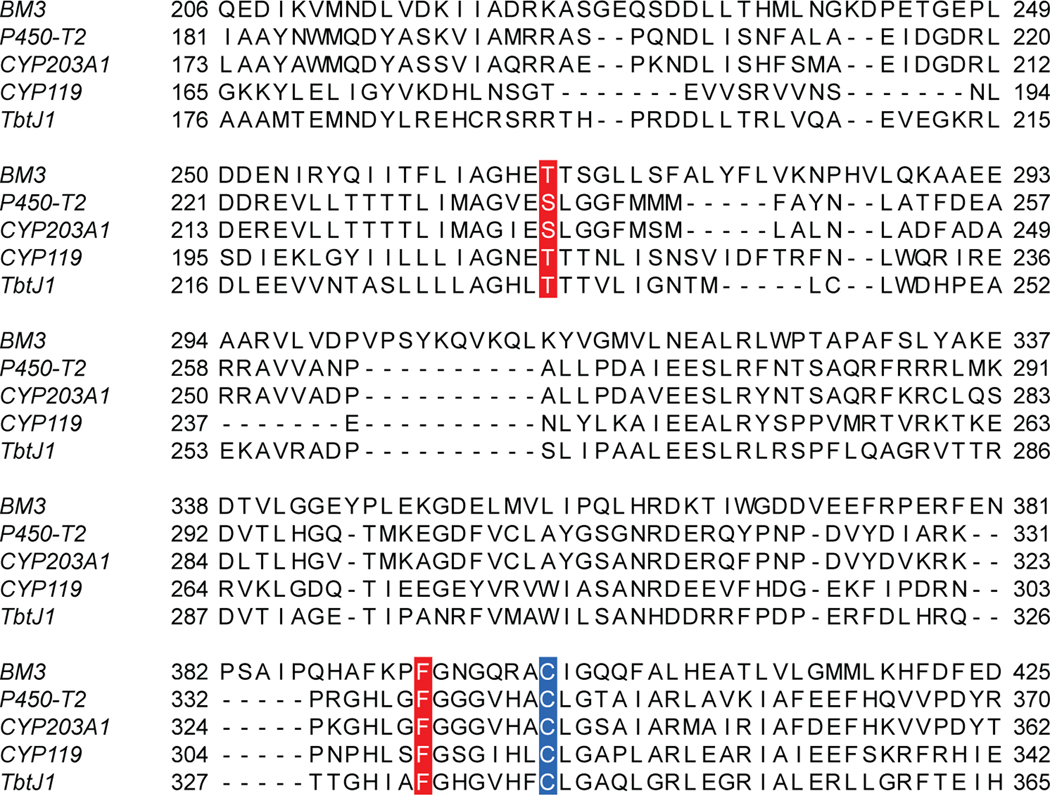
Conserved amino acids selected for saturation mutagenesis. The residue labeled in blue is the heme ligand cysteine in those P450s. Residues labeled in red are two conserved residues previously reported to affect the catalytic behavior in P450 BM3. Sequences were aligned with Clustal Omega^64^.

**Extended Data Fig. 8 |:**
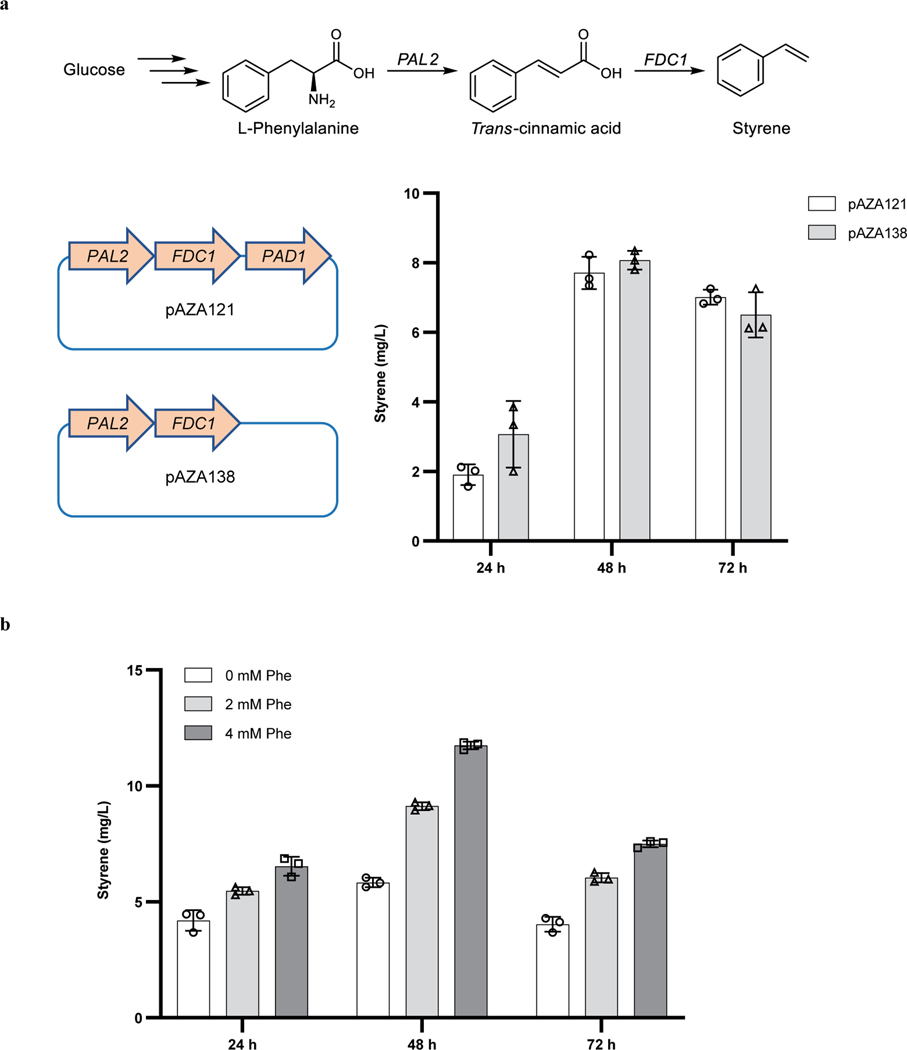
Exploration of the conditions for styrene biosynthesis in *S. albus*. **a,** Heterologously expressing *PAL2* and *FDC1* are sufficient to generate styrene. pAZA121 and pAZA138 are two integration plasmids used to introduce the styrene pathway into *S. albus*. **b,** Production of styrene by engineered *S. albus* grown in TSB medium supplemented with additional 0 mM, 2 mM or 4 mM Phe. Data are mean ± s.d.; *n* = 3 biological samples.

**Extended Data Fig. 9 |:**
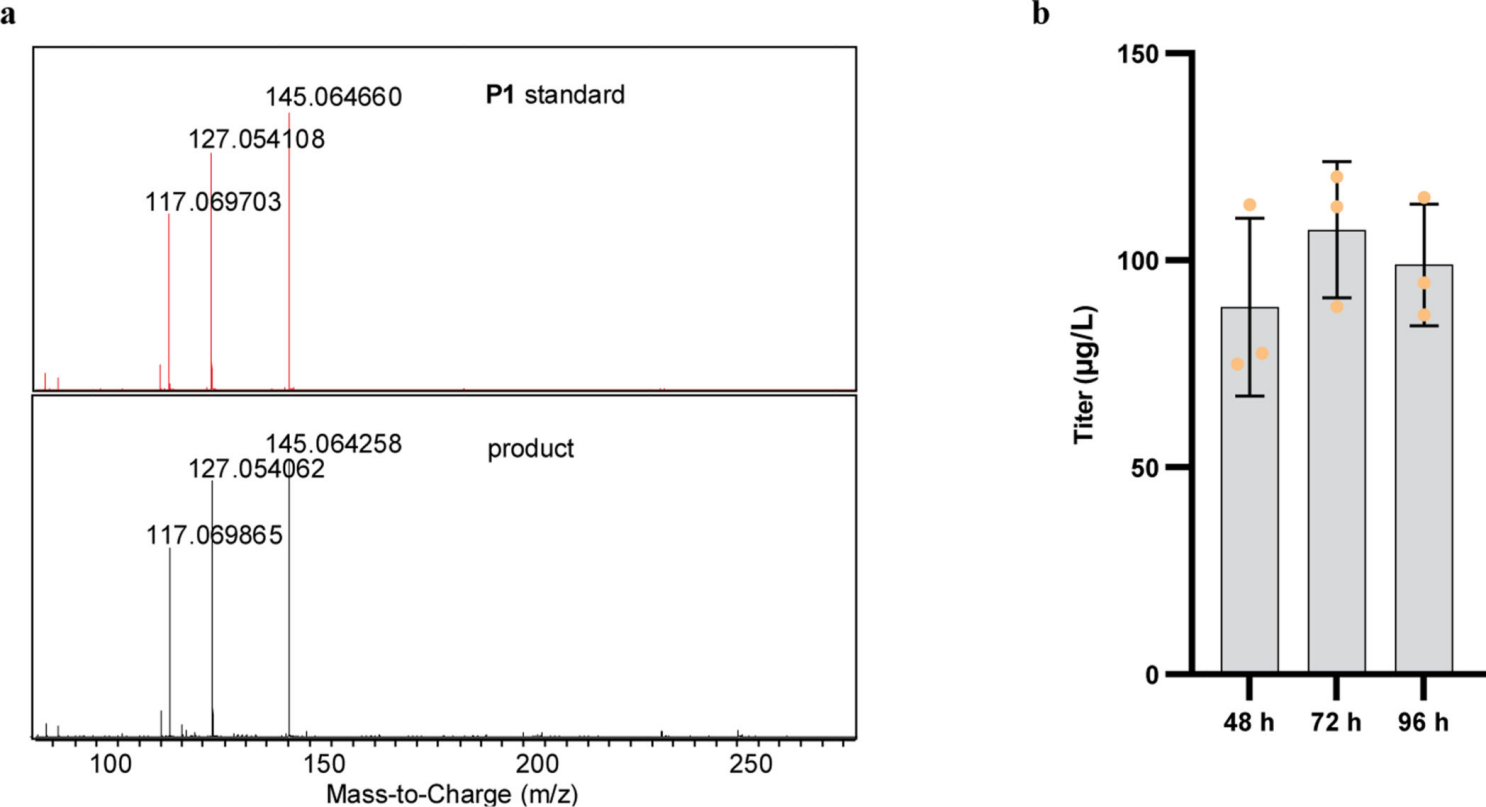
Characterization and time course of accumulation of the final products. **a,** MS/MS (20 eV) spectra of the **P1** standard (red) and the biosynthesized major product (black). **b,** Biosynthesis of final products during the 96-h fermentation process. The 24-h data are not presented because small quantities of product were observed (<10 μg/L), and thus the titer could not be accurately calculated. 1B medium with 4 mM Phe was used to generate the final products. Data are mean ± s.d.; *n* = 3 biological samples.

**Extended Data Table 1 |:** Screened sites for directed evolution of P450-T2.

Round	Parent	Screened sites	Selected mutation
1	P450-T2 WT	G347, S239	S239V
2	S239V	A235, E238, F285	No
3	S239V	F384, T231, T77, L70, F338	F338H
4	S239V, F338H	N65, P71, R73	P71D
5	S239V, F338H, P71D	K176, L66, S381, T383	T383E

## Supplementary Material

Supplementary information

## Figures and Tables

**Fig. 1 |:**
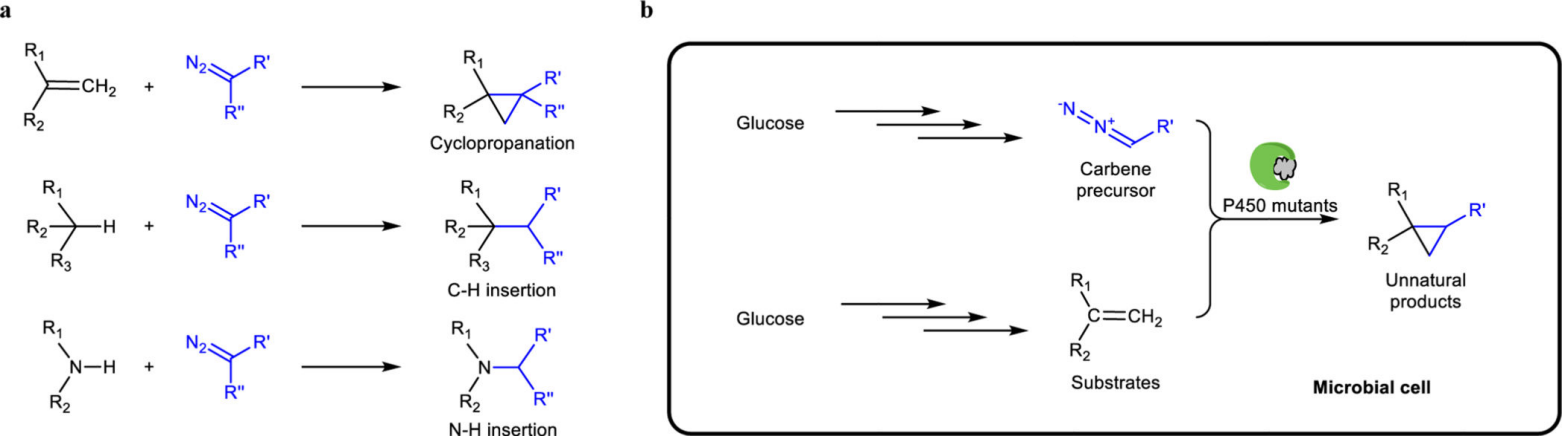
Schematic diagram of carbene transfer reactions for biosynthesis. **a,** Examples of recently developed carbene transfer reactions catalyzed by engineered cytochrome P450s^14^. **b,** Schematic diagram for integrating carbene transfer reactions into biosynthesis with all components in the reaction produced by microbial cells.

**Fig. 2 |:**
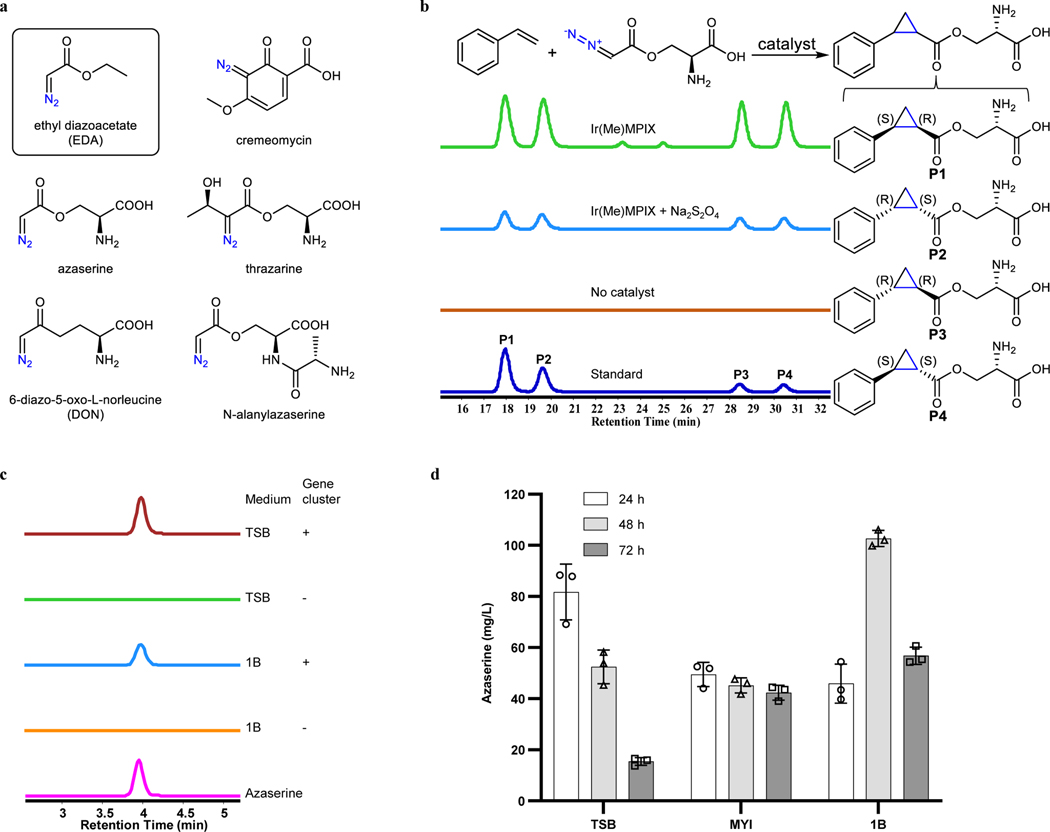
Heterologous biosynthesis of the carbene precursor azaserine. **a,** Selected diazo compounds found in nature^24^, except for EDA (in black rectangle). **b,** Azaserine acts as a carbene precursor for cyclopropanation. Extracted ion chromatogram (EIC, [M+H]^+^, *m*/*z* 250.1074) for target products. Representative traces are for 2 repeated reactions. **P1** to **P4** are the four diastereomers formed by cyclopropanation of styrene with azaserine (shown on the right). The reaction contained 5 mM styrene, 5 mM azaserine, 10 μM Ir(Me)MPIX or no catalyst, 0 or 10 mM Na_2_S_2_O_4_, 5 vol% ethanol, and M9-N buffer and was conducted at 22 °C under aerobic conditions for 18 h. Standard indicates a mixture of the four chemically synthesized diastereomers. **c,** Biosynthesis of azaserine in *S. albus* harboring the identified azaserine gene cluster in its genome. EIC ([M+H]^+^, *m*/*z* 174.0509) for azaserine. Representative traces for two repeated experiments. **d,** Azaserine production in three different culture media. Data are mean ± s.d.; *n* = 3 biological replicates.

**Fig. 3 |:**
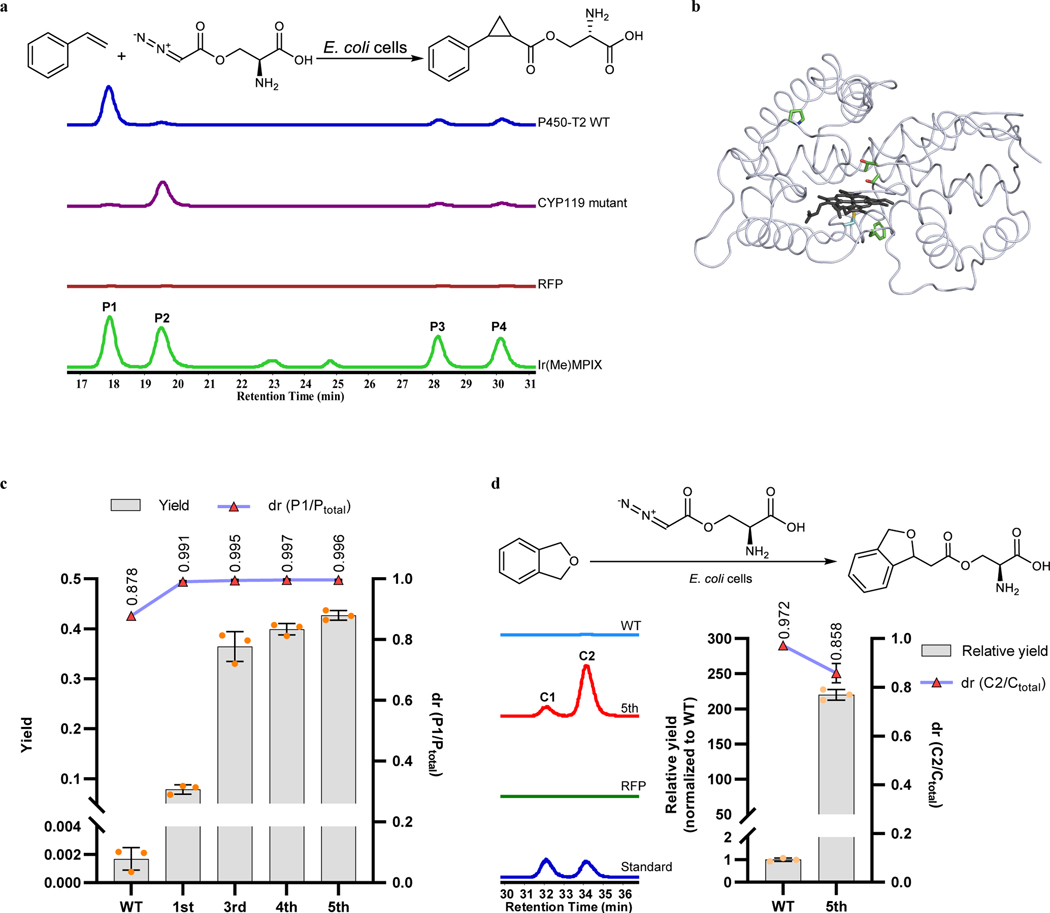
Engineering cytochrome P450s to catalyze carbene transfer reactions with azaserine as the carbene precursor. **a,** Screening P450s for catalyzing the reaction of styrene with azaserine. See the list of screened P450s and their mutants in Supplementary Table 1. EIC ([M+H]^+^, *m*/*z* 250.1074) for target products of reactions catalyzed by *E. coli* cells expressing different proteins. Representative traces are for two repeated experiments. RFP, *E. coli cells* expressing RFP (red fluorescence protein) as negative control; Ir(Me)MPIX, trace of the reaction products using Ir(Me)MPIX (with Na_2_S_2_O_4_) as catalyst for comparison. **P1** to **P4** are the four diastereomers formed by cyclopropanation of styrene with azaserine. **b,** Crystal structure of P450-T2 (PDB: 8FBC). Residues in green are mutation sites in the final evolved mutant. **c,** Directed evolution of P450-T2 for cyclopropanation of styrene with azaserine. **d,** P450-T2 WT and P450-T2–5 mutant for insertion of the carbene unit into the *sp*^*3*^ C–H bond of phthalan. **C1** and **C2** are the two diastereomers formed by the reaction of phthalan with azaserine. EIC ([M+H]^+^, *m*/*z* 266.1023) for target products. Representative traces for 3 biological repeats (left). Reaction conditions: 5 mM styrene or 10 mM pthalan, 5 mM azaserine, *E. coli* cells expressing different P450s or RFP (as control) with concentration of 30 OD_600_ (optical density at 600 nm) as catalysts, 5 vol% ethanol, M9-N buffer, conducted at 22 °C under aerobic conditions for 18 h. P_total_ and C_total_, sum area for all corresponding diastereomers. In (**c**) and (**d**), numbers on the horizontal axis represent the selected mutants in each round of directed evolution (for the specific mutations, see Extended Data Table 1). Data are mean ± s.d.; *n* = 3 biological replicates.

**Fig. 4 |:**
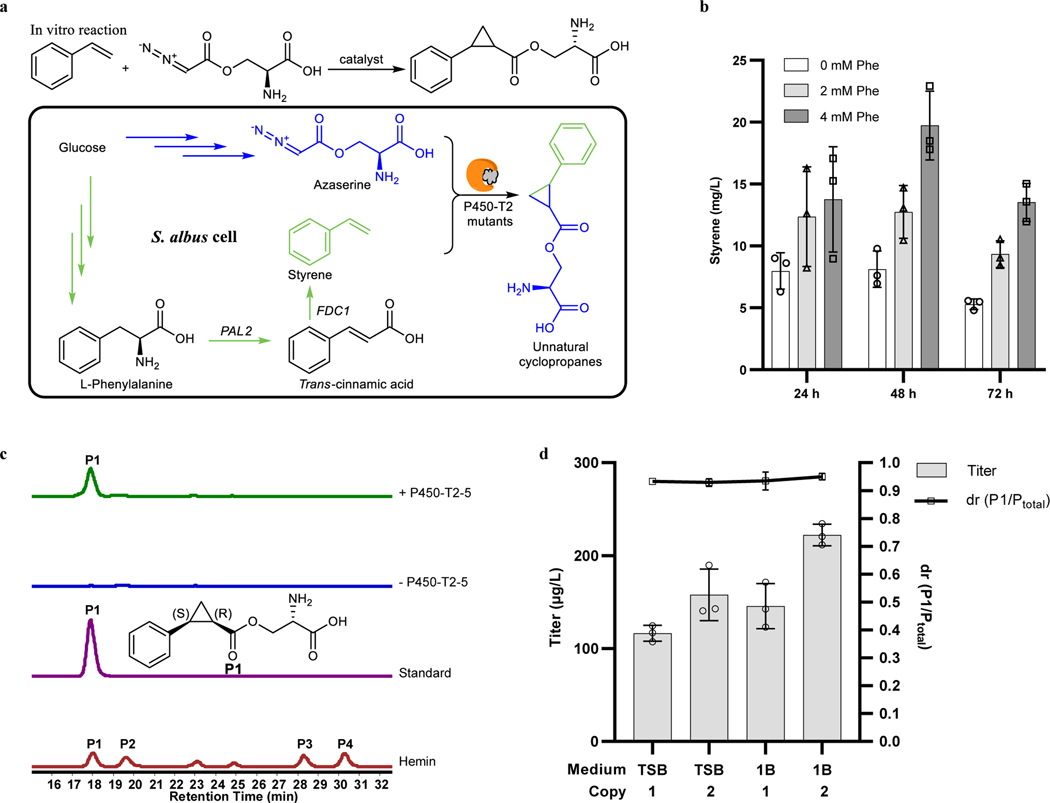
Biosynthesis of unnatural cyclopropanes by an abiological carbene transfer reaction. **a,** Schematic diagram for producing final products with substrates and enzymes made by the cells. **b,** Styrene biosynthesis in *S. albus*. 1B medium supplemented with 0 mM, 2 mM or 4 mM phenylalanine (Phe) for styrene production. **c,** Bio-production of the unnatural cyclopropanes. 1B medium with 4 mM Phe was used to generate the final products. EIC ([M+H]^+^, *m*/*z* 250.1074) for target products produced by the styrene-, azaserine-producing strain with or without P450-T2–5 mutant. Representative traces for 3 biological replicates. Standard, chemically synthesized single diastereomer standard of **P1**; hemin, trace of the reaction products of azaserine with styrene using hemin as catalyst (with Na_2_S_2_O_4_) for comparison. **d,** Final product titer optimization. TSB or 1B medium was used for azaserine-, styrene-producing *S. albus* strains with 1 or 2 copies of P450-T2–5 to generate the final products. **P1** to **P4** are the four diastereomers formed by cyclopropanation of styrene with azaserine. P_total_, sum area for all diastereomers. Grey bars indicate the titer (**b** and **d**); white square indicates the dr (**d**). Data are mean ± s.d.; *n* = 3 biological replicates.

## Data Availability

The DNA sequences of plasmids used in this study have been deposited in the public version of the JBEI registry (http://public-registry.jbei.org). Please refer to Supplementary Table 2 for accession codes (Part ID). The sequences and annotation of the *aza* BGC is available at the GenBank accession number NZ_BEVZ01000003.1 spanning base 82400 to111549. The determined structure has been deposited in the Protein Data Bank (accession code 8FBC). The mass spectrometry proteomics data have been deposited to the ProteomeXchange Consortium via the PRIDE^63^ partner repository with the dataset identifier PXD037509.
