# Supplementary material for: Complete integration of carbene transfer chemistry into biosynthesis
Source: Nature. Author manuscript; Available in PMC 2024 Aug 20. (PMC11334723; doi:10.1038/s41586-023-06027-2)
Supplement: Supplementary information [file NIHMS2008550-supplement-Supplementary_information.pdf]

Supplementary information

Complete integration of carbene transfer chemistry into biosynthesis

Authors: Jing Huang[1,2,3], Andrew Quest[4,5], Pablo Cruz-Morales[1,2,6], Kai Deng[2,7], Jose Henrique Pereira[2,8], Devon Van Cura[9], Ramu Kakumanu[1,2], Edward E. K. Baidoo[1,2], Qingyun Dan[1,2], Yan Chen[1,2], Christopher J. Petzold[1,2], Trent R. Northen[2,11], Paul D. Adams[2,8,12], Douglas S. Clark[3,8]*, Emily P. Balskus[9,10], John F. Hartwig[4,5]*, Aindrila Mukhopadhyay[1,2,11]*, Jay D. Keasling[1,2,3,6,12,13]*

Affiliations:

[1]Biological Systems and Engineering Division, Lawrence Berkeley National Laboratory, Berkeley, CA, USA.

[2]Joint BioEnergy Institute, Lawrence Berkeley National Laboratory, Emeryville, CA, USA.

[3]Department of Chemical and Biomolecular Engineering, University of California, Berkeley, CA, USA.

[4]Department of Chemistry, University of California, Berkeley, CA, USA.

[5]Chemical Sciences Division, Lawrence Berkeley National Laboratory, Berkeley, CA, USA.

[6]Novo Nordisk Foundation Center for Biosustainability, Technical University of Denmark, Kongens Lyngby, Denmark.

[7]Department of Biomaterials and Biomanufacturing, Sandia National Laboratories, Livermore, CA, USA.

[8]Molecular Biophysics and Integrated Bioimaging Division, Lawrence Berkeley National Laboratory, Berkeley, CA, USA.

[9]Department of Chemistry and Chemical Biology, Harvard University, Cambridge, MA, USA.

[10]Howard Hughes Medical Institute, Harvard University, Cambridge, MA, USA.

[11]Environmental Genomics and Systems Biology Division, Lawrence Berkeley National Laboratory, Berkeley, CA, USA.

[12]Department of Bioengineering, University of California, Berkeley, CA, USA.

[13]Synthetic Biochemistry Center, Institute for Synthetic Biology, Shenzhen Institutes for Advanced Technologies, Shenzhen, China.

*Correspondence to: dsc@berkeley.edu, jhartwig@berkeley.edu, amukhopadhyay@lbl.gov, keasling@berkeley.edu

Table of contents:

Supplementary Table 1 Initially screened P450s	Page 4
Supplementary Table 2 Plasmids used in this study	Page 4-5
Supplementary Table 3 Data collection and refinement statistics	Page 5
DNA and protein sequences	Page 6-8
Synthesis of compounds	Page 9-31
Supplementary Fig. 1 LC-MS traces of authentic aza-sty products	Page 32
NMR spectra (Supplementary Fig. 2 to 37)	Page 33-68
Supplementary Fig. 38 Raw gel image	Page 69
References	Page 69-70

Supplementary Table 1 | Initially screened P450s

Protein	Mutations	Description
TbtJ1[65]	NO	
TbtJ1[65]	C340S	
P450-T2[66]	NO	
CYP119[67]	T213G, V254A, L155W, R256W	
CYP119[67]	T213G, V254A, L155W, R256W, C317S	Mutant shown in Fig. 3a
P450 BM3[68]	T268A, C400S, L437W, V78M, L181V	Only heme domain of P450 BM3
P450 BM3[68]	T268A, C400S, L437W, V78M, L181V	Full length P450 BM3

Supplementary Table 2 | Plasmids used in this study

Name	Part ID[a]	Description
pAZA007	JBx_233142	Plasmid containing azaserine gene cluster from *S. fragilis* (other genes: apramycin resistance gene, phi C31 integrase gene). Individual genes in the azaserine gene cluster (aza1 to aza23) with Part ID from JBx_232920 to JBx_232942.
pAZA121 and pAZA138	JBx_233144 and JBx_236844	Plasmid containing genes for styrene biosynthesis in *S. albus* (other genes: spectinomycin resistance gene, VWB integrase gene)
pAZA128	JBx_233146	Plasmid containing evolved P50-T2-5 mutant gene and genes for styrene biosynthesis in *S. albus* (other genes: spectinomycin resistance gene, VWB integrase gene)
pAZA132	JBx_233148	Plasmid containing 2 copies of evolved P50-T2-5 mutant gene and genes for styrene biosynthesis in *S. albus* (other genes: spectinomycin resistance gene, VWB integrase gene)
pAZA037	JBx_233150	Plasmid containing P450-T2 WT gene under control of T7 promoter for expression in *E. coli*

pAZA076	JBx_233862	Plasmid containing CYP203A1 WT gene under control of T7 promoter for expression in *E. coli*

[a]The accession code in the public version of JBEI registry (http://public-registry.jbei.org).

Supplementary Table 3 | Data collection and refinement statistics

	**P450-T2***
Data collection	
Space group	P 2 21 21
Unit-Cell parameters (Å)	a=85.93 b=95.61 and c=100.43
Resolution range (Å)	41.78 - 1.53 (1.585 - 1.53)
R_{merge} (%)	0.059 (1.56)
I/σ*I*	15.7 (0.9)
Completeness (%)	100 (99.7)
Redundancy	7.0 (5.4)
$CC_{1/2}$	0.999 (0.367)
Refinement	
Resolution range (Å)	41.78 - 1.53 (1.585 - 1.53)
Reflections used in refinement	119251 (10768)
R_{work}	0.160 (0.238)
R_{free}	0.186 (0.283)
No. atoms	
Proteins	6188
Ligands/ion	146
Water	1035
RMS from ideal geometry	
Bond lengths (Å)	0.006
Bond angles (º)	0.85
Average B-factor	26.7
Macromolecules	25.4
Ligands	16.1
Solvent	34.3

* Data from a single crystal was used for P450-T2 structure.
# Values in parentheses are for highest-resolution shell.

DNA and protein sequences

P450-T2 protein sequence (on pAZA037, residues underlined as N-terminal tag, residue in bold numbered as the 1st residue)

MKSSHHHHHHGSSG**M**GLGSFHFDPYSPAIDADPFPSYKRLRDEFPCFWSEEAQMWILSR
YSDIVTAGQDWQTYSSASGNLMTELPGRAGATLGSSDPPKHDRLRGLIQHAFMKRNLM
ALEEPIRDVAKQVFAQVKGVKEFDFKDVSSQFTVKVLMAALGLPMGEDALVPEHEVRE
NAVLMVQSDARTRAKGPEHIAAYNWMQDYASKVIAMRRASPQNDLISNFALAEIDGDR
LDDREVLLTTTTLIMAGVESLGGFMMMFAYNLATFDEARRAVVANPALLPDAIEESLRF
NTSAQRFRRRLMKDVTLHGQTMKEGDFVCLAYGSGNRDERQYPNPDVYDIARKPRGH
LGFGGGVHACLGTAIARLAVKIAFEEFHQVVPDYRRVADQLPWMPSSTFRSPLVLQLKA
Q*

Codon optimized DNA sequence of P450-T2 for expression in *E. coli*

ATGAAATCTTCTCACCATCACCATCACCATGGTAGTTCGGGCATGGGATTAGGTAGC
TTCCACTTCGACCCCTACAGTCCGGCGATCGACGCTGACCCCTTCCCTAGTTATAAA
CGCTTGCGCGATGAGTTCCCCTGCTTCTGGTCTGAAGAGGCCCAAATGTGGATTCTT
TCGCGCTACTCTGATATCGTCACTGCGGGTCAGGACTGGCAAACCTATTCATCGGCC
AGCGGGAACTTAATGACTGAATTGCCGGGTCGCGCAGGCGCAACTCTTGGGTCTTCC
GACCCACCGAAACACGATCGCTTGCGTGGGCTTATTCAGCACGCGTTCATGAAACGT
AACCTGATGGCGTTGGAAGAGCCAATTCGCGACGTCGCGAAACAGGTTTTCGCGCA
AGTGAAAGGAGTAAAGGAGTTTGACTTTAAGGACGTATCTTCTCAGTTTACTGTCAA
GGTTTTGATGGCCGCGTTGGGGCTGCCCATGGGAGAAGATGCACTGGTACCAGAGC
ATGAAGTTCGCGAAAACGCAGTTCTGATGGTGCAATCGGACGCTCGCACTCGCGCG
AAGGGACCTGAGCACATTGCGGCATACAACTGGATGCAAGACTACGCATCAAAAGT
AATTGCTATGCGTCGCGCGAGCCCCCAAAATGACCTGATTAGCAATTTCGCGCTTGC
CGAGATTGATGGAGATCGTTTGGATGATCGCGAGGTGTTACTGACTACAACCACGCT
TATTATGGCCGGAGTGGAGAGCTTAGGTGGTTTCATGATGATGTTCGCATACAATCT

GGCAACTTTCGACGAGGCCCGCCGTGCGGTGGTTGCGAATCCTGCGTTGTTGCCTGA
CGCGATCGAAGAGTCACTTCGTTTCAACACGTCGGCCCAACGCTTCCGTCGCCGCCT
GATGAAAGATGTGACACTGCATGGCCAGACTATGAAGGAAGGTGATTTCGTTTGTCT
GGCCTACGGTTCTGGGAACCGTGACGAGCGTCAATATCCCAACCCAGACGTTTATGA
CATTGCCCGCAAGCCACGTGGGCATCTTGGCTTTGGAGGAGGTGTTCATGCCTGTTT
AGGCACAGCTATCGCACGCCTGGCGGTCAAGATCGCATTTGAAGAGTTTCACCAGGT
AGTCCCCGATTACCGTCGCGTGGCCGATCAGTTGCCGTGGATGCCCTCCTCCACCTT
CCGTTCACCTTTGGTGCTTCAGTTGAAAGCCCAGTAG

Codon optimized DNA sequence of P450-T2-5 for expression in *S. albus*

ATGGGGCTCGGCTCGTTCCACTTCGACCCCTACTCGCCCGCGATCGACGCGGACCCC
TTCCCGTCCTACAAGCGGCTGCGCGACGAATTCCCCTGCTTCTGGTCCGAAGAAGCC
CAGATGTGGATCCTCTCGCGGTACAGCGACATCGTGACCGCCGGCCAGGACTGGCA
GACCTACTCCTCCGCCAGCGGCAACCTCATGACGGAACTCGACGGGCGCGCGGGGG
CGACCCTCGGGTCCTCGGACCCCCCGAAGCACGACCGGCTGCGCGGCCTGATCCAG
CACGCCTTCATGAAGCGCAACCTGATGGCGCTCGAGGAGCCGATCCGCGACGTGGC
CAAGCAGGTGTTCGCCCAGGTGAAGGGGGTCAAGGAATTCGACTTCAAGGACGTCT
CGAGCCAGTTCACCGTGAAGGTGCTGATGGCCGCGCTCGGCCTGCCGATGGGCGAG
GACGCGCTCGTCCCGGAACACGAAGTGCGGGAAAACGCCGTCCTCATGGTCCAGTC
GGACGCGCGCACCCGGGCCAAGGGGCCCGAGCACATCGCCGCGTACAACTGGATGC
AGGACTACGCCTCGAAGGTCATCGCCATGCGCCGCGCGTCCCCGCAGAACGACCTG
ATCTCGAACTTCGCCCTCGCCGAGATCGACGGCGACCGCCTCGACGACCGGGAAGT
GCTGCTGACCACGACCACCCTGATCATGGCGGGGGTCGAGGTCCTCGGGGGCTTCAT
GATGATGTTCGCGTACAACCTCGCGACCTTCGACGAAGCGCGGCGCGCCGTCGTCGC
GAACCCGGCGCTGCTGCCCGACGCCATCGAGGAATCCCTGCGCTTCAACACCTCCGC
CCAGCGGTTCCGGCGCCGCCTGATGAAGGACGTCACGCTGCACGGGCAGACCATGA
AGGAAGGCGACTTCGTGTGCCTGGCCTACGGGAGCGGGAACCGGGACGAACGGCA
GTACCCCAACCCCGACGTGTACGACATCGCGCGGAAGCCCCGGGGGCACCTGGGGC
ACGGGGGTGGTGTCCACGCGTGCCTGGGTACGGCCATCGCCCGGCTGGCCGTCAAG
ATCGCGTTCGAAGAATTCCACCAGGTGGTCCCGGACTACCGGCGCGTCGCGGACCA

GCTCCCGTGGATGCCGTCCAGCGAGTTCCGGAGCCCCCTCGTCCTCCAGCTCAAGGC
GCAGTGA

Synthesis of authentic standards for the product (aza-sty, 1, 2, 3, 4 compounds correspond to P1, P2, P3, P4 in the manuscript) of the reaction of azaserine with styrene:

Unless stated otherwise, all chemicals, salts, and solvents were obtained from commercial suppliers (e.g. Sigma-Aldrich, Ambeed, Strem Chemicals) and used without further purification. Ethyl diazoacetate was purchased from Sigma-Aldrich as an 87 wt% solution in DCM and was used without further purification. Tetrahydrofuran and dichloromethane were purified by passage through a solvent column comprised of activated alumina, degassed over a copper column, and stored over activated 4Å molecular sieves under an atmosphere of nitrogen for 24 h before use. All air- and moisture-sensitive manipulations were conducted using standard Schlenk techniques under an atmosphere of nitrogen. Solvents and solutions were transferred using air-tight syringes. All flame-dried vessels were placed under vacuum and externally heated using a propane flame. All reactions were performed in flame-dried glassware under an atmosphere of nitrogen and were stirred using Teflon-coated magnetic stirring bars unless otherwise stated. Reactions were monitored by thin layer chromatography (TLC) on Kieselgel 60 F254 glass plates precoated with 0.25 mm thickness of silica gel and visualized by UV irradiation. Flash column chromatography was conducted with a Teledyne Isco CombiFlash R_f 200 System using SiliCycle Silia*Sep* Premium 25 µm columns.

Nuclear magnetic resonance (NMR) spectra were acquired on a Bruker NEO at 500 MHz for ^{1}H and 126 MHz for ^{13}C or on a Bruker AV-600 at 600 MHz for ^{1}H and 151 MHz for ^{13}C at the NMR facility of the College of Chemistry, University of California, Berkeley. Chemical shifts were reported in ppm downfield of TMS and were referenced to residual solvent signal (^{1}H-NMR: $CDCl_3$ δ = 7.76 ppm, $CD_3C(O)OD$ = 2.03 ppm, CD_3OD = 3.31 ppm; ^{13}C-NMR: $CDCl_3$ δ = 77.16 ppm, $CD_3C(O)OD$ = 20.00 ppm, CD_3OD = 49.00 ppm). NMR Spectra are reported as follows: chemical shift (multiplicity, coupling constants where applicable, number of nuclei). Splitting is reported with the following symbols: s = singlet, d = doublet, t = triplet, q = quartet, dd = doublet of doublets, dt = doublet of triplets, td = triplet of doublets, m = multiplet. Coupling constants are reported in Hz. High resolution mass spectrometry (HRMS) was performed on a Perkin Elmer axION ESI TOF at the LBNL Catalysis Laboratory, University of California, Berkeley. HRMS data only provided for newly reported compounds. Optical rotation was measured on a Perkin-Elmer 241 Automatic Polarimeter. Sample concentration for polarimetry is given in c = g/mL. Fourier Transform Infrared (FTIR) spectrometry was performed on a Bruker Vertex80 Time-

Resolved FTIR at the LBNL Catalysis Laboratory, University of California, Berkeley. FTIR spectra are reported as follows: transmission wavenumber (cm^{-1}), relative intensity, and characteristic bond (e.g. C=O). Relative transmission intensity is reported with the following symbols: br = broad, s = strong, vs = very strong. Only selected resonances are reported. High Performance Liquid Chromatography Mass Spectroscopy (HPLC-MS) analyses were performed on either an Agilent 1260 infinity ii with mass selective detector iQ (MSD iQ) or Agilent 6545 with quadrupole time-of-flight (Q-TOF) with a MilliporeSigma Astec CYCLOBOND I 2000 HP-RSP Chiral HPLC (5 µm, 25 cm x 4.6 mm) analytical column. The mobile phase consisted of 10 mM ammonium formate in water with 50 µL formic acid added per liter (solvent A) and 0.1% (v/v) formic acid in acetonitrile (solvent B), and separations were performed as follows: held at 14% B from 0 min to 32 min. The flow rate was held at 0.35 mL/min from 0 min to 23 min, increased to 0.8 mL/min in 0.2 min, held at 0.8 mL/min from 23.2 min to 32 min. Retention time (T_r) is reported as detection time (in minutes) after sample injection.

Synthesis of cis-ethyl-2-phenylcyclopropane-1-carboxylate (**S1**) **and trans-ethyl-2-phenyl-cyclopropane-1-carboxylate** (**S2**)

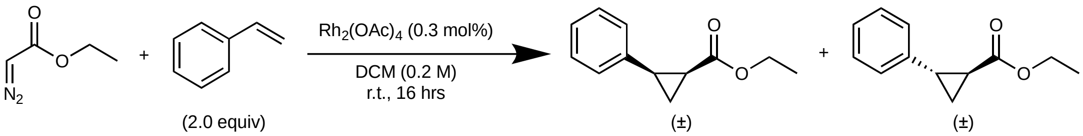

S1 **S2**

To a flame-dried, 500-mL round-bottom flask with magnetic stirring bar was added $Rh_2(OAc)_4$ (134 mg, 0.303 mmol, 0.3 mol%). The flask was placed under vacuum and backfilled with nitrogen three times before 400 mL of dry, degassed dichloromethane (0.25 M) was added, producing a light-green suspension of catalyst. The flask was then flushed with nitrogen for 10 min under constant stirring. Styrene (22.9 mL, 200 mmol, 2.00 equiv) was added to the reaction flask and allowed to stir for 10 min at room temperature. Ethyl diazoacetate (12.1 mL, 100 mmol, 1.00 equiv) was then slowly added (0.80 mL per hour) over 15 h at room temperature under nitrogen using a syringe pump. Once all ethyl diazoacetate had been added, the reaction was allowed to stir under nitrogen for an additional 1 h, during which the reaction maintained a consistent heterogenous blue-green color. The reaction mixture was concentrated to approximately 200 mL under reduced pressure and filtered through silica gel with 100 mL of dichloromethane, giving a yellow-brown solution. This solution was then concentrated under reduced pressure to a

viscous, yellow oil. The crude residue was purified by flash column chromotagraphy (gradient 0%→10% diethyl ether:hexanes) to yield **S1** (5.83 g, 30.7 mmol, 31% yield) as a colorless oil and **S2** (9.00 g, 47.3 mmol, 47% yield) as a colorless, crystalline solid.

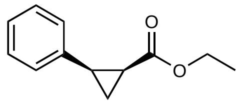

(±)

S1

Data for cis-ethyl-2-phenylcyclopropane-1-carboxylate (**S1**)

^{1}H NMR (500 MHz, $CDCl_3$) δ 7.29 – 7.22 (m, 4H), 7.18 (ddt, *J* = 8.6, 5.6, 2.9 Hz, 1H), 3.86 (q, *J* = 7.1 Hz, 2H), 2.57 (td, *J* = 9.0, 7.5 Hz, 1H), 2.07 (ddd, *J* = 9.3, 7.8, 5.6 Hz, 1H), 1.77 – 1.65 (m, 1H), 1.31 (td, *J* = 8.2, 5.0 Hz, 1H), 0.96 (t, *J* = 7.1 Hz, 3H).

^{13}C NMR (126 MHz, $CDCl_3$) δ 171.09, 136.69, 129.42, 127.99, 126.75, 60.28, 25.58, 21.92, 14.13, 11.22.

FTIR (neat, cm^{-1}) $\boldsymbol{\upsilon}_{\mathbf{max}}$: 1722 vs (C=O), 1175 vs (C–O)

R_f (10:90 ethyl acetate:hexanes): 0.40

^{1}H-NMR and ^{13}C-NMR of **S1** were consistent with those reported previously[69].

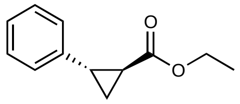

(±)

S2

Data for trans-ethyl-2-phenylcyclopropane-1-carboxylate (**S2**)

^{1}H NMR (500 MHz, $CDCl_3$) δ 7.26 (dd, *J* = 8.4, 6.8 Hz, 2H), 7.21 – 7.15 (m, 1H), 7.12 – 7.04 (m, 2H), 4.16 (q, *J* = 7.1 Hz, 2H), 2.51 (ddd, *J* = 9.2, 6.5, 4.1 Hz, 1H), 1.89 (ddd, *J* = 8.3, 5.3, 4.1 Hz, 1H), 1.59 (dt, *J* = 9.5, 4.9 Hz, 1H), 1.40 – 1.15 (m, 4H).

^{13}C NMR (126 MHz, $CDCl_3$) δ 173.47, 140.21, 128.55, 126.55, 126.24, 60.77, 26.25, 24.27, 17.14, 14.36.

FTIR (neat, cm^{-1}) $\boldsymbol{\upsilon}_{\mathbf{max}}$: 1714 vs (C=O), 1175 vs (C–O)

R_f (10:90 ethyl acetate:hexanes): 0.44

^{1}H-NMR and ^{13}C-NMR of **S2** were consistent with those reported previously[70].

Synthesis of cis-2-phenylcyclopropane-1-carboxylic acid (**S3**)

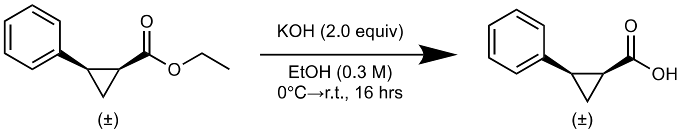

S1 **S3**

To a 50-mL, round-botton flask with magnetic stirring bar under an atmosphere of air was added potassium hydroxide (1.12 g, 20.0 mmol, 2.00 equiv). This material was dissolved in 23 mL of absolute ethanol (final reaction concentration of 0.30 M) at room temperature under vigorous stirring. Once the KOH had fully dissolved, the cloudy solution was cooled to 0 ℃ in an ice-water bath for 15 min. **S1** (1.90 g, 10.0 mmol, 1.00 equiv) was dissolved in 7 mL of absolute ethanol and was slowly added to the stiring ethanolic solution of KOH over 10 min. The reaction was allowed to warm to room temperature overnight, during which time it turned light-orange. The reaction mixture was diluted with diH_2O and transferred to a separatory funnel. The aqueous solution was extracted three times with dichlormethane to remove uncharged impurities. The organic fractions were combined and back-extracted with 0.5 M NaOH; the organic fractions were discarded. The aqueous layers were combined and acidified to pH 1 with 6 N HCl, producing a cloudy, white suspension. This suspension was extracted 5 times with dichloromethane. The organic fractions were combined and sequentially washed with 1 N HCl and brine, dried over $MgSO_4$, and concentrated under reduced pressure to yield a light-orange residue. This residue was recrystallized from boiling pentanes to give **S3** (1.48 g, 9.14 mmol, 91% yield) as off-white needles.

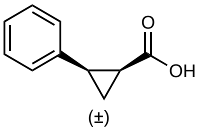

S3

Data for cis-2-phenylcyclopropane-1-carboxylic acid (**S3**)

^{1}H NMR (500 MHz, $CDCl_3$) δ 7.30 – 7.20 (m, 5H), 2.65 (q, *J* = 8.6 Hz, 1H), 2.05 (ddd, *J* = 9.2, 7.7, 5.6 Hz, 1H), 1.68 (dt, *J* = 7.7, 5.3 Hz, 1H), 1.39 (ddd, *J* = 8.7, 7.7, 5.0 Hz, 1H).

^{13}C NMR (126 MHz, $CDCl_3$) δ 177.00, 136.01, 129.39, 128.08, 126.91, 26.70, 21.48, 12.18.

FTIR (neat, cm^{-1}) υ_{max}: 2936 br (O–H), 1688 vs (C=O), 1446 s (O–H)

R_f (20:80 ethyl acetate:hexanes): 0.17

^{1}H-NMR and ^{13}C-NMR of **S3** were consistent with those reported previously[71].

Synthesis of trans-2-phenylcyclopropane-1-carboxylic acid (S4)

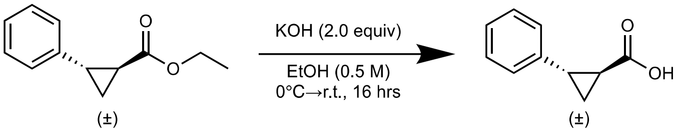

S2 **S4**

To a 100-mL, round-botton flask with magnetic stirring bar under an atmosphere of air was added potassium hydroxide (4.49 g, 80.0 mmol, 2.00 equiv). This material was dissolved in 60 mL of absolute ethanol (final reaction concentration of 0.50 M) at room temperature under vigorous stirring. Once the KOH had fully dissolved, the cloudy solution was cooled to 0 °C in an ice-water bath for 15 min. **S2** (1.90 g, 10.0 mmol, 1.00 equiv) was dissolved in 20 mL of absolute ethanol and was slowly added to the stiring ethanolic solution of KOH over 10 min. The reaction was allowed to warm to room temperature overnight, during which time it became light yellow alongside formation of a white precipitate. The reaction mixture was diluted with diH_2O, dissolving the precipitate, and transferred to a separatory funnel. The aqueous solution was extracted three times with dichlormethane to remove uncharged impurities. The organic fractions were combined and back-extracted with 0.5 M NaOH; the organic fractions were discarded. The aqueous layers were combined and acidified to pH 1 with 6 N HCl, producing a cloudy, white suspension. This suspension was extracted 5 times with dichloromethane. The organic fractions were combined and sequentially washed with 1 N HCl and brine, dried over $MgSO_4$, and concentrated under reduced pressure to yield a yellow residue. This residue was recrystallized from boiling pentanes to give **S4** (5.38 g, 33.2 mmol, 83% yield) as a light-yellow crystalline solid.

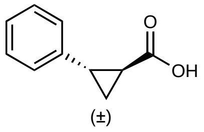

S4

Data for trans-2-phenylcyclopropane-1-carboxylic acid (**S4**)

^{1}H NMR (500 MHz, $CDCl_3$) δ 11.81 (s, 1H), 7.26 (t, *J* = 7.6 Hz, 2H), 7.21 – 7.16 (m, 1H), 7.08 (dd, *J* = 7.3, 1.8 Hz, 2H), 2.58 (ddd, *J* = 9.3, 6.7, 4.1 Hz, 1H), 1.88 (ddd, *J* = 8.8, 5.2, 4.0 Hz, 1H), 1.64 (dt, *J* = 9.6, 4.9 Hz, 1H), 1.38 (ddd, *J* = 8.4, 6.7, 4.6 Hz, 1H).

^{13}C NMR (126 MHz, $CDCl_3$) δ 180.18, 139.62, 128.67, 126.84, 126.41, 27.25, 24.14, 17.65.

FTIR (neat, cm^{-1}) $\boldsymbol{\upsilon_{max}}$: 2938 br (O–H), 1687 vs (C=O), 1446 s (O–H)

R_f (20:80 ethyl acetate:hexanes): 0.16

^{1}H-NMR and ^{13}C-NMR of **S4** were consistent with those reported previously[71].

Synthesis of (*S*)-4-benzyl-3-((1*R*,2*S*)-2-phenylcyclopropane-1-carbonyl)oxazolidin-2-one (S5) and (*S*)-4-benzyl-3-((1*S*,2*R*)-2-phenylcyclopropane-1-carbonyl)oxazolidin-2-one (S6)

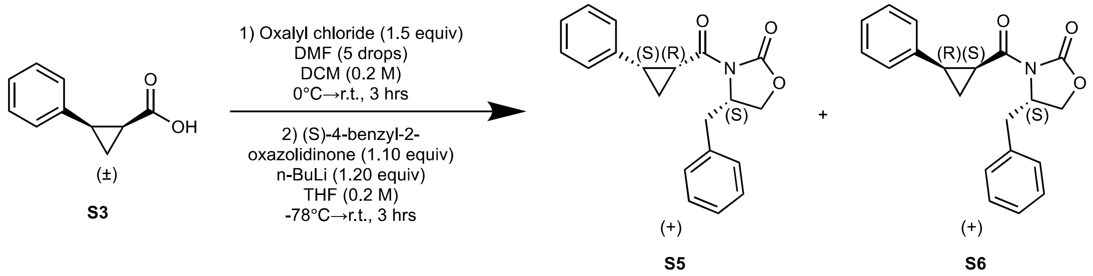

To a flame-dried, 50-mL round-bottom flask containing a magnetic stirring bar were added **S3** (1.30 g, 8.00 mmol, 1.00 equiv) and 40 mL of dry, degassed dichloromethane (0.20 M). This flask was flushed with nitrogen for 10 min under constant stirring, at which time the solution was cooled to 0 °C in an ice-water bath for 15 min. Once cooled, 5 drops of dimethylformamide were added, and the solution was allowed to stir for a further 5 min at 0 °C under an atmosphere of nitrogen. Oxalyl chloride (1.03 mL, 12.0 mmol, 1.50 equiv) was slowly added dropwise to the stirring reaction mixture over the course of 10 min, resulting in the yellowing of the reaction mixture and evolution of gas. This reaction was allowed to warm to room temperature over 3 h while stirring. The solution of acyl chloride was concentrated under reduced pressure and dried under vacuum to give an orange oil. The crude acyl chloride was dissolved in 10 mL of dry, degassed tetrahydrofuran.

To a flame-dried, 100-mL round-bottom flask equipped with magnetic stirring bar were added (S)-4-benzyl-2-oxazolidine (1.56 g, 8.80 mmol, 1.10 equiv) and 30 mL of dry, degassed tetrahydrofuran (final reaction concentration of 0.20 M). This solution was flushed with nitrogen for 10 min under constant stirring at room temperature, at which time the solution was cooled to -78 °C in a dry ice/acetone bath for 20 min. A 2.5 M solution of n-butyl lithium in hexanes (3.84 mL, 9.60 mmol, 1.20 equiv) was added dropwise over 10 min, generating a white precipitate. This reaction mixture was allowed to warm to room temperature under nitrogen over 1 h before cooling the reaction mixture to -78 °C in a dry ice/acetone bath for 20 min. Simultaneously, the solution of acyl chloride in THF was cooled to -78 °C in a dry ice/acetone bath under an atmosphere of nitrogen for 20 min. Once cooled, the solution of acyl chloride was added slowly to the stirring

solution of lithiated oxazolidinone over the course of 5 min, during which time the reaction became light yellow/orange. The reaction mixture was allowed to stir at -78 °C under an atmosphere of nitrogen for 1 h before warming to room temperature over an additional 1 h. The reaction was quenched with the addition of 20 mL of diH_2O, and the resulting mixture was allowed to stir for 10 min. This mixture was transferred to a separatory funnel and extracted 5 times with dichloromethane. The organic layers were combined and sequentially washed with 1 N NaOH, 1 N HCl, diH_2O, and brine. The organic layer then was dried over $MgSO_4$ and concentrated to a light-brown solid. The residue was purified by column chromatography (5%→15% ethyl acetate:hexanes) to give **S5** (1.04 g, 3.22 mmol, 81% yield with respect to starting enantiomer of **S3**) as a granular, colorless solid, and **S6** (1.11 g, 3.44 mmol, 86% yield with respect to starting enantiomer of **S3**) as a colorless, crystalline solid.

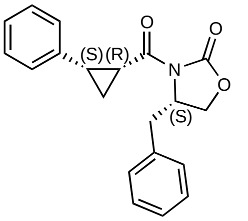

(+)

S5

Data for (*S*)-4-benzyl-3-((1*R*,2*S*)-2-phenylcyclopropane-1-carbonyl)oxazolidin-2-one (**S5**)

^{1}H NMR (500 MHz, $CDCl_3$) δ 7.35 – 7.21 (m, 7H), 7.20 – 7.14 (m, 1H), 7.00 – 6.94 (m, 2H), 4.51 – 4.38 (m, 1H), 4.18 – 4.08 (m, 1H), 4.04 (dd, *J* = 9.1, 2.3 Hz, 1H), 3.36 (ddd, *J* = 9.8, 7.3, 5.9 Hz, 1H), 2.90 (dt, *J* = 9.7, 7.8 Hz, 1H), 2.51 (dd, *J* = 13.5, 3.3 Hz, 1H), 2.15 – 2.02 (m, 2H), 1.45 (ddd, *J* = 8.4, 7.3, 5.2 Hz, 1H).

^{13}C NMR (126 MHz, $CDCl_3$) δ 169.17, 153.68, 135.92, 135.66, 129.37, 129.19, 128.89, 128.08, 127.17, 126.92, 66.02, 55.47, 37.28, 27.15, 24.19, 10.33.

FTIR (neat, cm^{-1}) υ_{max}: 1769 vs (C=O), 1687 vs (C=O)

HRMS (ESI): *m/z* for $C_{20}H_{19}NNaO_3^+$ $[M+Na]^+$ calcd.: 344.1257, found: 344.1268.

$[\alpha]_D^{25}$ = +152 degrees (c = 0.010, $CHCl_3$)

R_f (20:80 ethyl acetate:hexanes): 0.28

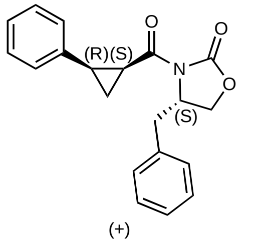

S6

Data of (*S*)-4-benzyl-3-((1*S*,2*R*)-2-phenylcyclopropane-1-carbonyl)oxazolidin-2-one (**S6**)

^{1}H NMR (500 MHz, $CDCl_3$) δ 7.34 – 7.17 (m, 8H), 7.17 – 7.08 (m, 2H), 4.31 (ddt, *J* = 9.5, 7.9, 3.3 Hz, 1H), 4.03 (dd, *J* = 9.0, 3.1 Hz, 1H), 3.92 (t, *J* = 8.5 Hz, 1H), 3.40 (ddd, *J* = 9.6, 7.5, 5.9 Hz, 1H), 3.19 (dd, *J* = 13.4, 3.5 Hz, 1H), 2.81 (q, *J* = 8.4 Hz, 1H), 2.70 (dd, *J* = 13.4, 9.6 Hz, 1H), 2.02 (dt, *J* = 7.4, 5.5 Hz, 1H), 1.47 (td, *J* = 8.0, 5.1 Hz, 1H).

^{13}C NMR (126 MHz, $CDCl_3$) δ 169.51, 153.80, 136.18, 135.33, 129.51, 128.97, 128.95, 128.16, 127.34, 126.84, 65.99, 55.06, 37.97, 27.23, 23.83, 11.18.

FTIR (neat, cm^{-1}) $\boldsymbol{\upsilon_{max}}$: 1771 vs (C=O), 1685 vs (C=O)

HRMS (ESI): *m/z* for $C_{20}H_{19}NNaO_3^+$ $[M+Na]^+$ calcd.: 344.1257, found: 344.1249.

$\mathbf{[\alpha]_D^{25}}$ = +14.3 degrees (c = 0.014, $CHCl_3$)

$\mathbf{R_f}$ (20:80 ethyl acetate:hexanes): 0.49

Synthesis of (*S*)-4-benzyl-3-((1*R*,2*R*)-2-phenylcyclopropane-1-carbonyl)oxazolidin-2-one (S7) and (*S*)-4-benzyl-3-((1*S*,2*S*)-2-phenylcyclopropane-1-carbonyl)oxazolidin-2-one (S8)

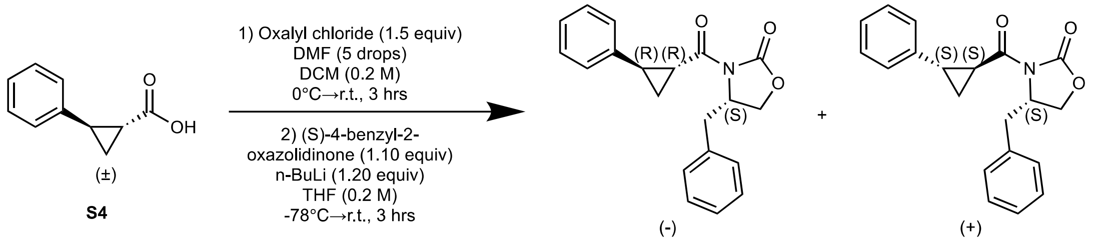

S4 **S7** **S8**

To a flame-dried, 100-mL round-bottom flask containing a magnetic stirring bar were added **S4** (2.17 g, 13.4 mmol, 1.00 equiv) and 70 mL of dry, degassed dichloromethane (0.2 M). This flask was flushed with nitrogen for 10 min under constant stirring, at which time the solution was cooled to 0 ℃ in an ice-water bath for 15 min. Once cooled, 10 drops of dimethylformamide were added, and the solution was allowed to stir for a further 5 min at 0 ℃ under an atmosphere of nitrogen. Oxalyl chloride (1.73 mL, 20.1 mmol, 1.50 equiv) was slowly added dropwise to the

stirring reaction mixture over the course of 10 min, resulting in the yellowing of the reaction mixture and evolution of gas. This reaction was allowed to warm to room temperature over 3 h while stirring. The solution of acyl chloride was concentrated under reduced pressure and dried under vacuum to give a dark-red oil. The crude acyl chloride was dissolved in 15 mL of dry, degassed tetrahydrofuran.

To a flame-dried, 100-mL round-bottom flask equipped with magnetic stirring bar were added (S)-4-benzyl-2-oxazolidine (2.60 g, 14.7 mmol, 1.10 equiv) and 50 mL of dry, degassed tetrahydrofuran (final reaction concentration of 0.2 M). This solution was flushed with nitrogen for 10 min under constant stirring at room temperature, at which time the solution was cooled to -78 ℃ in a dry ice/acetone bath for 20 min. A 2.5 M solution of n-butyl lithium in hexanes (6.43 mL, 16.1 mmol, 1.20 equiv) was added dropwise over 10 min, generating a white precipitate. This reaction mixture was allowed to warm to room temperature under nitrogen over 1 h before cooling the reaction mixture to -78 ℃ in a dry ice/acetone bath for 20 min. Simultaneously, the solution of acyl chloride in THF was cooled to -78 ℃ in a dry ice/acetone bath under an atmosphere of nitrogen for 20 min. Once cooled, the solution of acyl chloride was added slowly to the stirring solution of lithiated oxazolidinone over the course of 5 min, during which time the reaction became red/orange in color. The reaction mixture was allowed to stir at -78 ℃ under an atmosphere of nitrogen for 1 h before warming to room temperature over an additional 1 h. The reaction was quenched with the addition of 30 mL of diH_2O, and the resulting mixture was allowed to stir for 10 min. This mixture was transferred to a separatory funnel and extracted 5 times with dichloromethane. The organic layers were combined and sequentially washed with 1 N NaOH, 1 N HCl, diH_2O, and brine. The organic layer then was dried over $MgSO_4$ and concentrated to a light-brown solid. The residue was purified by column chromatography (5%→20% ethyl acetate:hexanes) to give **S7** (2.02 g, 6.30 mmol, 94% yield with respect to starting enantiomer of **S4**) as a colorless, fibrous solid and **S8** (1.63 g, 5.08 mmol, 76% yield with respect to starting enantiomer of **S4**) as a colorless, needle-like solid.

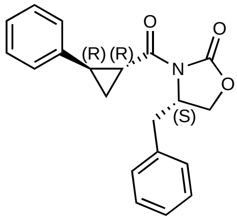

(-)

S7

Data of (*S*)-4-benzyl-3-((1*R*,2*R*)-2-phenylcyclopropane-1-carbonyl)oxazolidin-2-one (**S8**)

^{1}H NMR (500 MHz, $CDCl_3$) δ 7.38 – 7.22 (m, 10H), 4.75 (ddt, *J* = 9.4, 7.7, 3.2 Hz, 1H), 4.30 – 4.15 (m, 2H), 3.62 (ddd, *J* = 8.3, 5.2, 4.2 Hz, 1H), 3.33 (dd, *J* = 13.4, 3.5 Hz, 1H), 2.84 (dd, *J* = 13.4, 9.5 Hz, 1H), 2.77 (ddd, *J* = 9.3, 6.6, 4.1 Hz, 1H), 1.81 (ddd, *J* = 9.3, 5.2, 4.1 Hz, 1H), 1.48 (ddd, *J* = 8.3, 6.6, 4.1 Hz, 1H).

^{13}C NMR (126 MHz, $CDCl_3$) δ 172.78, 153.89, 139.82, 135.42, 129.55, 129.06, 128.63, 127.44, 126.78, 126.68, 66.20, 55.60, 38.16, 29.10, 23.09, 19.26.

FTIR (neat, cm^{-1}) $\boldsymbol{\upsilon}_{\mathbf{max}}$: 1781 vs (C=O), 1674 vs (C=O)

HRMS (ESI): *m/z* for $C_{20}H_{19}NNaO_3^+$ $[M+Na]^+$ calcd.: 344.1257, found: 344.1244.

$[\boldsymbol{\alpha}]_D^{25}$ = -156 degrees (c = 0.011, $CHCl_3$)

R_f (20:80 ethyl acetate:hexanes): 0.37

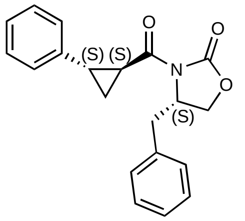

(+)

S8

Data of (*S*)-4-benzyl-3-((1*S*,2*S*)-2-phenylcyclopropane-1-carbonyl)oxazolidin-2-one (**S7**)

^{1}H NMR (500 MHz, $CDCl_3$) δ 7.46 – 7.17 (m, 10H), 4.74 (ddt, *J* = 10.3, 6.8, 3.3 Hz, 1H), 4.29 – 4.13 (m, 2H), 3.63 (ddd, *J* = 8.1, 5.2, 4.1 Hz, 1H), 3.37 (dd, *J* = 13.4, 3.4 Hz, 1H), 2.85 (dd, *J* = 13.4, 9.6 Hz, 1H), 2.75 (ddd, *J* = 9.2, 6.6, 4.2 Hz, 1H), 1.86 (ddd, *J* = 9.3, 5.3, 4.1 Hz, 1H), 1.55 (ddd, *J* = 8.3, 6.6, 4.1 Hz, 1H).

^{13}C NMR (126 MHz, $CDCl_3$) δ 172.75, 153.91, 139.78, 135.48, 129.56, 129.08, 128.61, 127.46, 126.77, 126.60, 66.18, 55.81, 38.10, 28.99, 23.11, 19.42.

FTIR (neat, cm^{-1}) $\boldsymbol{\upsilon}_{\mathbf{max}}$: vs 1758 (C=O), 1673 vs (C=O)

HRMS (ESI): *m/z* for $C_{20}H_{20}NO_3^+$ $[M+H]^+$ calcd.: 322.1438, found: 322.1424.

$[\alpha]_D^{25}$ = +302 degrees (c = 0.011, $CHCl_3$)

R_f (20:80 ethyl acetate:hexanes): 0.46

Synthesis of (1*R*,2*S*)-2-phenylcyclopropane-1-carboxylic acid (S9)

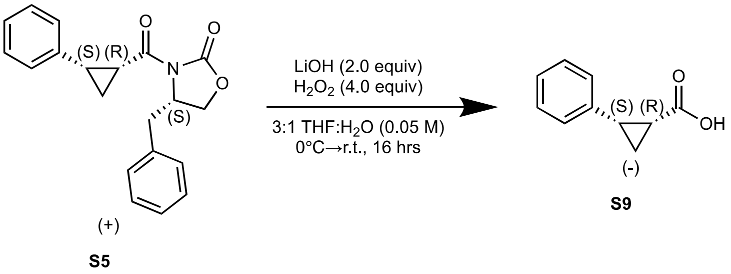

Procedure modified from Evans *et al*[72]. To a 50-mL, round-bottom flask containing a magnetic stirring bar under an atmsophere of air were added **S5** (0.579 g, 1.80 mmol, 1.0 equiv) and 36 mL of 3:1 THF:H_2O (0.05 M). This solution was allowed to stir at room temperature for 5 min before 30% H_2O_2 (0.735 mL, 7.20 mmol, 4.0 equiv) was added. LiOH (86.2 mg, 3.60 mmol, 2.0 equiv) was then added to the reaction mixture as one portion and allowed to stir at room temperature for 16 h. Sodium sulfite (998 mg, 7.92 mmol, 4.4 equiv) was dissolved in 5 mL of diH_2O and added to the reaction mixture. The resulting solution was allowed to stir at room temperature for an additional 10 min. The reaction mixture was transferred to a separatory funnel, 10 mL of 1 N NaOH was added, and the aqueous layer was extracted with dichlormethane twice to remove neutral organic products. The organic layers were combined and back-extracted with 0.5 N NaOH twice; the organic fraction was then discarded. The aqueous fractions were combined, acidified to pH 1 with 6 N HCl, producing a cloudy, white suspension, and extracted 5 times with dichloromethane. The organic fractions were combined and washed with 1 N HCl and brine, then dried over $MgSO_4$ and concentrated under reduced pressure to give a colorless solid. This residue was recrystallized from boiling pentanes to give **S9** (0.249 g, 1.54 mmol, 85% yield) as a fibrous, colorless solid.

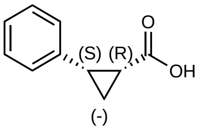

S9

Data for (1*R*,2*S*)-2-phenylcyclopropane-1-carboxylic acid (**S9**)

^{1}H NMR (500 MHz, $CDCl_3$) δ 11.04 (s, 1H), 7.32 – 7.20 (m, 5H), 2.64 (q, *J* = 8.6 Hz, 1H), 2.05 (ddd, *J* = 9.3, 7.7, 5.6 Hz, 1H), 1.68 (dt, *J* = 7.8, 5.4 Hz, 1H), 1.38 (ddd, *J* = 8.7, 7.7, 5.0 Hz, 1H).

^{13}C NMR (500 MHz, $CDCl_3$) δ 177.47, 136.02, 129.37, 128.05, 126.87, 26.68, 21.54, 12.16.

FTIR (neat, cm^{-1}) $\boldsymbol{\upsilon}_{\mathbf{max}}$: 2939 br (O–H), 1684 vs (C=O), 1442 s (O–H)

$[\alpha]_D^{25}$ = -30.0 degrees (c = 0.010, $CHCl_3$)

R_f (20:80 ethyl acetate:hexanes): 0.17

^{1}H-NMR and ^{13}C-NMR, and $[\alpha]_D^{25}$ of **S9** were consistent with those reported previously[73].

Synthesis of (1*S*,2*R*)-2-phenylcyclopropane-1-carboxylic acid (S10)

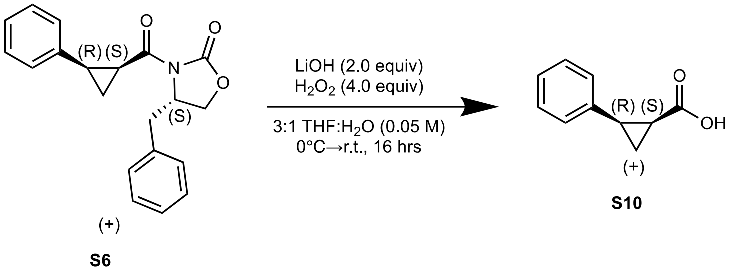

Procedure modified from Evans *et al*[72]. To a 50-mL, round-bottom flask containing a magnetic stirring bar under an atmsophere of air were added **S6** (0.656 g, 2.04 mmol, 1.0 equiv) and 41 mL of 3:1 THF:H_2O (0.05 M). This solution was allowed to stir at room temperature for 5 min before 30% H_2O_2 (0.833 mL, 8.17 mmol, 4.0 equiv) was added. LiOH (171 mg, 4.08 mmol, 2.0 equiv) was then added to the reaction mixture as one portion and allowed to stir at room temperature for 16 h. Sodium sulfite (1.13 g, 8.98 mmol, 4.4 equiv) was dissolved in 10 mL of diH_2O and added to the reaction mixture. The resulting solution was allowed to stir at room temperature for an additional 10 min. The reaction mixture was transferred to a separatory funnel, 10 mL of 1 N NaOH was added, and the aqueous layer was extracted with dichlormethane twice to remove neutral organic products. The organic layers were combined and back-extracted with 0.5 N NaOH twice; the organic fraction was then discarded. The aqueous fractions were combined, acidified to pH 1 with 6 N HCl, producing a cloudy, white suspension, and extracted 5 times with

dichloromethane. The organic fractions were combined and washed with 1 N HCl and brine, then dried over $MgSO_4$ and concentrated under reduced pressure to give a colorless solid. This residue was recrystallized from boiling pentanes to give **S10** (0.254 g, 1.56 mmol, 77% yield) as a fibrous, colorless solid.

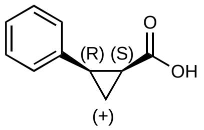

S10

Data for (1*S*,2*R*)-2-phenylcyclopropane-1-carboxylic acid (**S10**)

^{1}H NMR (500 MHz, $CDCl_3$) δ 10.66 (s, 1H), 7.32 – 7.13 (m, 5H), 2.60 (q, *J* = 8.6 Hz, 1H), 2.01 (ddd, *J* = 9.4, 7.7, 5.6 Hz, 1H), 1.64 (dt, *J* = 7.7, 5.3 Hz, 1H), 1.34 (ddd, *J* = 8.9, 7.7, 5.0 Hz, 1H).

^{13}C NMR (500 MHz, $CDCl_3$) δ 177.41, 136.03, 129.38, 128.06, 126.87, 26.68, 21.54, 12.16.

FTIR (neat, cm^{-1}) υ_{max}: 3026 br (O–H), 1683 vs (C=O), 1441 s (O–H)

$[\alpha]_D^{25}$ = +28.7 degrees (c = 0.010, $CHCl_3$)

R_f (20:80 ethyl acetate:hexanes): 0.17

^{1}H-NMR and ^{13}C-NMR, and $[\alpha]_D^{25}$ of **S10** were consistent with those reported previously[73].

Synthesis of (1*R*,2*R*)-2-phenylcyclopropane-1-carboxylic acid (S11)

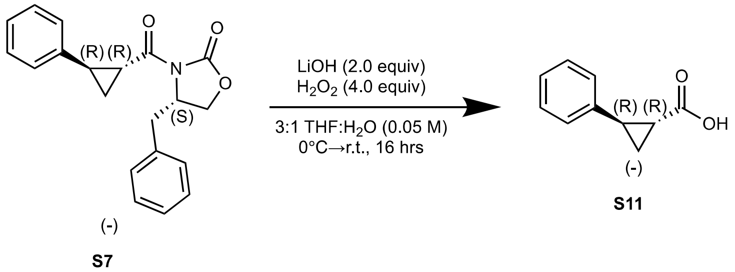

Procedure modified from Evans *et al*[72]. To a 100-mL, round-bottom flask containing a magnetic stirring bar under an atmsophere of air were added **S7** (1.01 g, 3.14 mmol, 1.0 equiv) and 60 mL of 3:1 THF:H_2O (0.05 M). This solution was allowed to stir at room temperature for 5 min before 30% H_2O_2 (1.28 mL, 12.6 mmol, 4.0 equiv) was added. LiOH (264 mg, 6.28 mmol, 2.0 equiv) was then added to the reaction mixture as one portion and allowed to stir at room temperature for 16 h. Sodium sulfite (1.74 g, 13.8 mmol, 4.4 equiv) was dissolved in 10 mL of diH_2O and added to the reaction mixture. The resulting solution was allowed to stir at room

temperature for an additional 10 min. The reaction mixture was transferred to a separatory funnel, 10 mL of 1 N NaOH was added, and the aqueous layer was extracted with dichlormethane twice to remove neutral organic products. The organic layers were combined and back-extracted with 0.5 N NaOH twice; the organic fraction was then discarded. The aqueous fractions were combined, acidified to pH 1 with 6 N HCl, producing a cloudy, white suspension, and extracted 5 times with dichloromethane. The organic fractions were combined and washed with 1 N HCl and brine, then dried over $MgSO_4$ and concentrated under reduced pressure to give a colorless solid. This residue was purified by column chromatography (5%→15% ethyl acetate:hexanes) to give **S11** (0.265 g, 1.63 mmol, 52% yield) as a viscous, light-yellow oil.

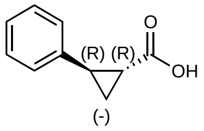

S11

Data for (1*R*,2*R*)-2-phenylcyclopropane-1-carboxylic acid (**S11**)

^{1}H NMR (500 MHz, $CDCl_3$) δ 11.69 (s, 1H), 7.26 (td, *J* = 7.2, 1.3 Hz, 2H), 7.22 – 7.16 (m, 1H), 7.11 – 7.05 (m, 2H), 2.58 (ddd, *J* = 9.2, 6.7, 4.1 Hz, 1H), 1.88 (ddd, *J* = 8.2, 5.2, 4.1 Hz, 1H), 1.64 (dt, *J* = 9.6, 4.9 Hz, 1H), 1.38 (ddd, *J* = 8.3, 6.7, 4.6 Hz, 1H).

^{13}C NMR (500 MHz, $CDCl_3$) δ 179.85, 139.84, 128.62, 126.72, 126.39, 27.00, 24.37, 17.57.

FTIR (neat, cm^{-1}) $\boldsymbol{\upsilon}_{\mathbf{max}}$: 3029 br (O–H), 1689 vs (C=O), 1427 s (O–H)

$[\alpha]_D^{25}$ = -378 degrees (c = 0.011, $CHCl_3$)

R_f (20:80 ethyl acetate:hexanes): 0.16

^{1}H-NMR and ^{13}C-NMR, and $[\alpha]_D^{25}$ of **S11** were consistent with those reported previously[73].

Synthesis of (1*S*,2*S*)-2-phenylcyclopropane-1-carboxylic acid (**S12**)

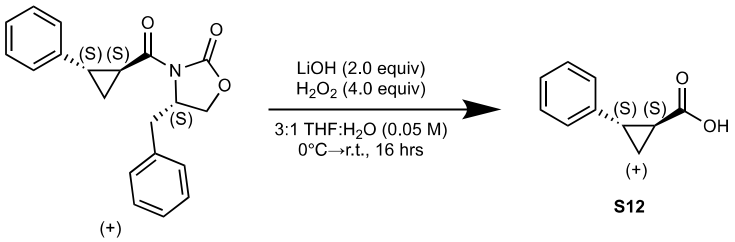

S8

Procedure modified from Evans *et al*[72]. To a 100-mL, round-bottom flask containing a magnetic stirring bar under an atmsophere of air were added **S8** (0.972 g, 3.02 mmol, 1.0 equiv) and 60 mL of 3:1 THF:H_2O (0.05 M). This solution was allowed to stir at room temperature for 5 min before 30% H_2O_2 (1.23 mL, 12.1 mmol, 4.0 equiv) was added. LiOH (254 mg, 6.05 mmol, 2.0 equiv) was then added to the reaction mixture as one portion and allowed to stir at room temperature for 16 h. Sodium sulfite (1.68 g, 13.3 mmol, 4.4 equiv) was dissolved in 10 mL of diH_2O and added to the reaction mixture. The resulting solution was allowed to stir at room temperature for an additional 10 min. The reaction mixture was transferred to a separatory funnel, 10 mL of 1 N NaOH was added, and the aqueous layer was extracted with dichlormethane twice to remove neutral organic products. The organic layers were combined and back-extracted with 0.5 N NaOH twice; the organic fraction was then discarded. The aqueous fractions were combined, acidified to pH 1 with 6 N HCl, producing a cloudy, white suspension, and extracted 5 times with dichloromethane. The organic fractions were combined and washed with 1 N HCl and brine, then dried over $MgSO_4$ and concentrated under reduced pressure to give a colorless solid. This residue was purified by column chromatography (5%→15% ethyl acetate:hexanes) give **S12** (0.328 g, 2.02 mmol, 67% yield) as a viscous, colorless liquid which solidified to a colorless solid after standing for 2 days.

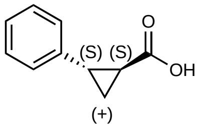

S12

Data for (1*S*,2*S*)-2-phenylcyclopropane-1-carboxylic acid (**S12**)

^{1}H NMR (500 MHz, $CDCl_3$) δ 11.33 (s, 1H), 7.26 (td, *J* = 7.3, 1.4 Hz, 2H), 7.22 – 7.16 (m, 1H), 7.11 – 7.05 (m, 2H), 2.58 (ddd, *J* = 9.3, 6.7, 4.1 Hz, 1H), 1.88 (ddd, *J* = 8.3, 5.2, 4.1 Hz, 1H), 1.64 (dt, *J* = 9.6, 4.9 Hz, 1H), 1.38 (ddd, *J* = 8.4, 6.7, 4.6 Hz, 1H).

^{13}C NMR (500 MHz, $CDCl_3$) δ 180.00, 139.64, 128.67, 126.84, 126.41, 27.24, 24.13, 17.64.

FTIR (neat, cm^{-1}) $\boldsymbol{\upsilon_{max}}$: 3029 br (O–H), 1686 vs (C=O), 1444 s (O–H)

$\boldsymbol{[\alpha]_D^{25}}$ = +388 degrees (c = 0.011, $CHCl_3$)

$\mathbf{R_f}$ (20:80 ethyl acetate:hexanes): 0.16

^{1}H-NMR and ^{13}C-NMR, and $[\alpha]_D^{25}$ of **S12** were consistent with those reported previously[73].

Synthesis of *O*-((1*R*,2*S*)-2-phenylcyclopropane-1-carbonyl)-L-serine (1)

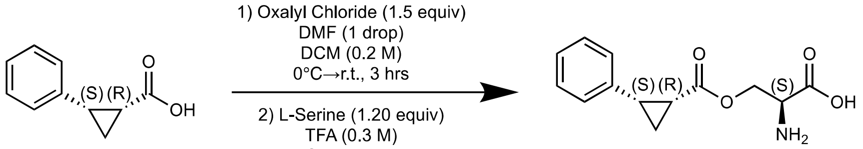

(-) **S9** **1**

To a flame-dried, 1-dram (3.7 mL) vial equipped with magnetic stirring bar was added **S9** (73.6 mg, 0.454 mmol, 1.0 equiv). The vial was placed under vacuum and backfilled with nitrogen 3 times. To the vial was added 2.3 mL of dry, degassed dichloromethane (0.2 M), and this solution was allowed to stir at room temperature under an atmosphere of nitrogen for 5 min. One drop of dimethylformamide was added to the reaction mixture, and the resulting mixture was allowed to stir for 2 min. The vial was allowed to cool to 0 °C in an ice-water bath for 10 min before oxalyl chloride (58 μL, 0.681 mmol, 1.5 equiv) was added dropwise over 5 min. The reaction was allowed to warm to room temperature over 3 h under an atmosphere of nitrogen. The reaction mixture was concentrated under reduced pressure to an orange residue and dried under vacuum to give a orange oil. This oil was dissolved in 0.5 mL of trifluroacetic acid.

To a separate, flame-dried, 1-dram vial containing a magnetic stirring bar was added L-serine (57.3 mg, 0.545 mmol, 1.2 equiv). The vial was placed under vacuum and backfilled with nitrogen 3 times. To the vial was added 1 mL of trifluoroacetic acid (final reaction concentration 0.3 M), and the resulting mixture was allowed to stir at room temperature until all of the L-serine had dissolved. The L-serine solution was then cooled to 0 °C in an ice-water bath under nitrogen for 30 min. In a separate vessel, the acyl chloride solution was allowed to cool to 0 °C in an ice-water bath under nitrogen for 30 min. The solution of acyl chloride was slowly added dropwise to the L-serine solution over 5 min, and this reaction mixture was allowed to warm to room temperature under an atmosphere of nitrogen for 16 h. After completion of the reaction, the reaction mixture was transferred to a beaker containing 10 mL of cold diethyl ether, causing precipitation of a yellow solid. The yellow solid was filtered through a fine glass frit and washed with diethyl ether. To this crude solid was added 5 mL of diH_2O, producing a yellow solution. Saturated $NaHCO_3$ was added until the solution reached pH 5.5, causing a light yellow solid to precipitate. The mother liquor was removed, and the yellow solid was recrystallized from boiling diH_2O to give **1** (22.6 mg, 0.091 mmol, 20% yield) as colorless, reflective crystals.

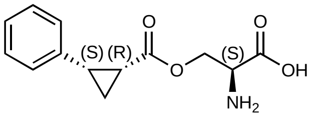

1

Data for *O*-((1*R*,2*S*)-2-phenylcyclopropane-1-carbonyl)-L-serine (**1**)

^{1}H NMR (500 MHz, acetic acid-d$_4$) δ 7.31 – 7.13 (m, 5H), 4.42 (dd, *J* = 12.3, 3.0 Hz, 1H), 4.25 (dd, *J* = 12.4, 5.3 Hz, 1H), 4.21 – 4.13 (m, 1H), 2.68 (q, *J* = 8.5 Hz, 1H), 2.18 (ddd, *J* = 9.1, 7.6, 5.5 Hz, 1H), 1.68 (dt, *J* = 7.5, 5.4 Hz, 1H), 1.41 (td, *J* = 8.2, 4.9 Hz, 1H).

^{13}C NMR (126 MHz, acetic acid-d$_4$) δ 172.55, 171.53, 137.29, 130.27, 129.02, 127.73, 63.45, 54.87, 27.11, 21.93, 12.32.

FTIR (neat, cm^{-1}) $\boldsymbol{\upsilon}_{\mathbf{max}}$: 3192 br (N–H), 2982 br (O–H), 1729 s (C=O), 1603 s (C=O)

HRMS (ESI): *m/z* for $C_{13}H_{15}NNaO_4^+$ $[M+Na]^+$ calcd.: 272.0893, found: 272.0893.

T_r (HPLC-MS): 18.1 min

The absolute configuration of the product is determined by the correlation to the known absolute configuration of the reactants.

Synthesis of *O*-((1*S*,2*R*)-2-phenylcyclopropane-1-carbonyl)-L-serine (2)

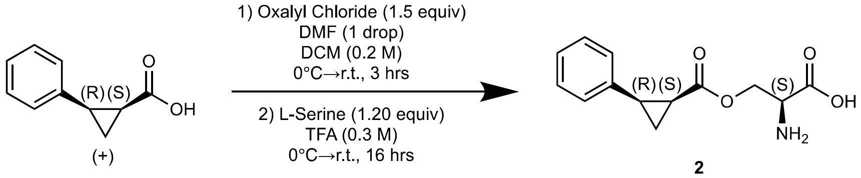

S10 **2**

To a flame-dried, 10-mL conical flask equipped with magnetic stirring bar was added **S10** (200 mg, 1.23 mmol, 1.0 equiv). The flask was placed under vacuum and backfilled with nitrogen 3 times. To the flask was added 6.2 mL of dry, degassed dichloromethane (0.2 M), and this solution was allowed to stir at room temperature under an atmosphere of nitrogen for 5 min. Five drops of dimethylformamide were added to the reaction mixture, and the resulting mixture was allowed to stir for 2 min. This solution was allowed to cool to 0 °C in an ice-water bath for 10 min before oxalyl chloride (0.158 mL, 1.85 mmol, 1.5 equiv) was added dropwise over 5 min. The reaction was allowed to warm to room temperature over 3 h under an atmosphere of nitrogen. The reaction mixture was concentrated under reduced pressure to an orange residue and dried under vacuum to give an orange oil. This oil was dissolved in 1.0 mL of trifluroacetic acid.

To a separate, flame-dried, 5-mL conical flask containing a magnetic stirring bar was added L-serine (0.155 g, 1.48 mmol, 1.2 equiv). The flask was placed under vacuum and backfilled with nitrogen 3 times. To the flask was added 3.0 mL of trifluoroacetic acid (final reaction concentration 0.3 M), and the resulting mixture was allowed to stir at room temperature until all of the L-serine had dissolved. The L-serine solution was then cooled to 0 °C in an ice-water bath under nitrogen for 30 min. In a separate vessel, the acyl chloride solution was allowed to cool to 0 °C in an ice-water bath under nitrogen for 30 min. The solution of acyl chloride was slowly added dropwise to the L-serine solution over 5 min, and this reaction mixture was allowed to warm to room temperature under an atmosphere of nitrogen for 16 h. After completion of the reaction, the reaction mixture was transferred to a beaker containing 20 mL of cold diethyl ether, causing precipitation of an off-white solid. The crude solid was filtered through a fine glass frit and washed with diethyl ether. To the crude solid was added 5 mL of diH_2O, producing a light-yellow solution. Saturated $NaHCO_3$ was added until the solution reached pH 5.5, causing a light yellow solid to precipitate. The mother liquor was removed, and the crude material was recrystallized from boiling diH_2O to give **2** (123 mg, 0.493 mmol, 40% yield) as colorless, reflective crystals.

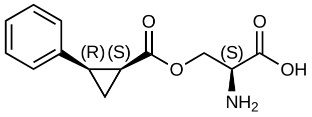

2

Data for *O*-((1*S*,2*R*)-2-phenylcyclopropane-1-carbonyl)-L-serine (**2**)

^{1}H NMR (500 MHz, acetic acid-d_4) δ 7.33 – 7.12 (m, 5H), 4.37 – 4.26 (m, 2H), 4.17 (dd, *J* = 4.9, 3.6 Hz, 1H), 2.70 (q, *J* = 8.5 Hz, 1H), 2.26 – 2.12 (m, 1H), 1.70 (dt, *J* = 7.7, 5.4 Hz, 1H), 1.43 (td, *J* = 8.2, 5.1 Hz, 1H).

^{13}C NMR (126 MHz, acetic acid-d_4) δ 172.67, 171.12, 136.97, 129.99, 128.87, 127.60, 63.19, 54.58, 27.08, 22.00, 12.29.

FTIR (neat, cm^{-1}) $\boldsymbol{\upsilon_{max}}$: 2982 br (O–H), 1728 s (C=O), 1601s (C=O)

HRMS (ESI): *m/z* for $C_{13}H_{15}NNaO_4^+$ $[M+Na]^+$ calcd.: 272.0893, found: 272.0894.

T_r (HPLC-MS): 19.7 min

The absolute configuration of the product is determined by the correlation to the known absolute configuration of the reactants.

Synthesis of *O*-((1*R*,2*R*)-2-phenylcyclopropane-1-carbonyl)-L-serine (3)

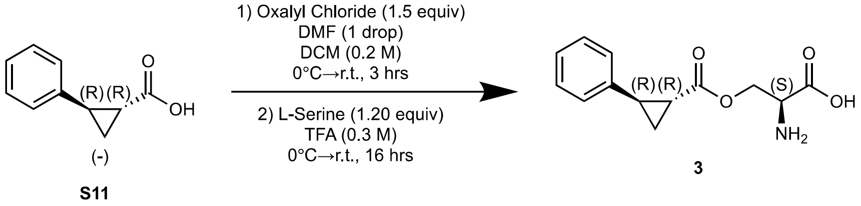

To a flame-dried, 10-mL conical flask equipped with magnetic stirring bar was added **S11** (200 mg, 1.23 mmol, 1.0 equiv). The flask was placed under vacuum and backfilled with nitrogen 3 times. To the flask was added 6.2 mL of dry, degassed dichloromethane (0.2 M), and this solution was allowed to stir at room temperature under an atmosphere of nitrogen for 5 min. Five drops of dimethylformamide were added to the reaction mixture, and the resulting mixture was allowed to stir for 2 min. This solution was allowed to cool to 0 °C in an ice-water bath for 10 min before oxalyl chloride (0.158 mL, 1.85 mmol, 1.5 equiv) was added dropwise over 5 min. The reaction was allowed to warm to room temperature over 3 h under an atmosphere of nitrogen. The reaction mixture was concentrated under reduced pressure to an orange residue and dried under vacuum to give an orange oil. This oil was dissolved in 1.0 mL of trifluroacetic acid.

To a separate, flame-dried, 5-mL conical flask containing a magnetic stirring bar was added L-serine (0.155 g, 1.48 mmol, 1.2 equiv). The flask was placed under vacuum and backfilled with nitrogen 3 times. 3.0 mL of trifluoroacetic acid (final reaction concentration 0.3 M) was added to the flask, and the resulting mixture was allowed to stir at room temperature until all of the L-serine had dissolved. The L-serine solution was then cooled to 0 °C in an ice-water bath under nitrogen for 30 min. In a separate vessel, the acyl chloride solution was allowed to cool to 0 °C in an ice-water bath under nitrogen for 30 min. The solution of acyl chloride was slowly added dropwise to the L-serine solution over 5 min, and this reaction mixture was allowed to warm to room temperature under an atmosphere of nitrogen for 16 h. After completion of the reaction, the reaction mixture was transferred to a beaker containing 20 mL of cold diethyl ether, causing precipitation of an off-white solid. The crude solid was filtered through a fine glass frit and washed with diethyl ether. 5 mL of diH_2O were added to the crude solid, producing a colorless solution. Saturated $NaHCO_3$ was added until the solution reached pH 5.5, causing an off-white solid to precipitate. The mother liquor was removed, and the crude material was recrystallized from boiling diH_2O to give **3** (24.1 mg, 0.097 mmol, 9% yield) as colorless, reflective crystals.

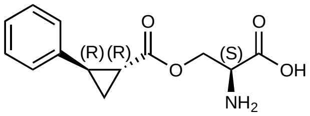

3

Data for *O*-((1*R*,2*R*)-2-phenylcyclopropane-1-carbonyl)-L-serine (**3**)

^{1}H NMR (500 MHz, acetic acid-d$_4$) δ 7.26 (t, *J* = 7.6 Hz, 1H), 7.18 (t, *J* = 7.4 Hz, 1H), 7.10 (d, *J* = 7.6 Hz, 1H), 4.71 – 4.56 (m, 1H), 2.62 – 2.48 (m, 1H), 1.94 (dt, *J* = 9.0, 4.9 Hz, 1H), 1.60 (dt, *J* = 9.7, 5.0 Hz, 1H), 1.41 (q, *J* = 6.7 Hz, 1H).

^{13}C NMR (126 MHz, acetic acid-d$_4$) δ 180.24, 139.73, 128.77, 126.95, 126.52, 27.36, 24.24, 17.75.

FTIR (neat, cm^{-1}) $\boldsymbol{\upsilon}_{\mathbf{max}}$: 3286 br (N–H), 3027 br (O–H), 1713 s (C=O), 1582 vs (C=O)

HRMS (ESI): *m/z* for $C_{13}H_{15}NNaO_4^+$ $[M+Na]^+$ calcd.: 272.0893, found: 272.0888.

T$_r$ (HPLC-MS): 28.1 min

The absolute configuration of the product is determined by the correlation to the known absolute configuration of the reactants.

Synthesis of *O*-((1*S*,2*S*)-2-phenylcyclopropane-1-carbonyl)-L-serine (4)

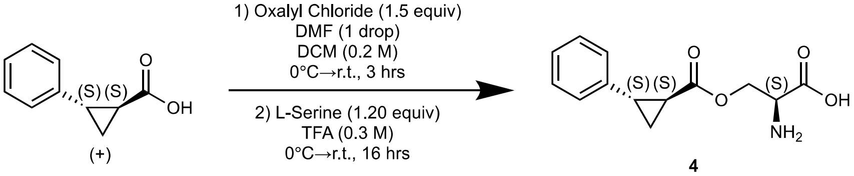

S12 **4**

To a flame-dried, 10-mL conical flask equipped with magnetic stirring bar was added **S12** (200 mg, 1.23 mmol, 1.0 equiv). The flask was placed under vacuum and backfilled with nitrogen 3 times. To the flask was added 6.2 mL of dry, degassed dichloromethane (0.2 M), and this solution was allowed to stir at room temperature under an atmosphere of nitrogen for 5 min. Five drops of dimethylformamide were added to the reaction mixture, and the resulting mixture was allowed to stir for 2 min. This solution was allowed to cool to 0 °C in an ice-water bath for 10 min before oxalyl chloride (0.158 mL, 1.85 mmol, 1.5 equiv) was added dropwise over 5 min. The reaction was allowed to warm to room temperature over 3 h under an atmosphere of nitrogen. The reaction mixture was concentrated under reduced pressure to an orange residue and dried under vacuum to give an orange oil. This oil was dissolved in 1.0 mL of trifluroacetic acid.

To a separate, flame-dried, 5-mL conical flask containing a magnetic stirring bar was added L-serine (0.155 g, 1.48 mmol, 1.2 equiv). The flask was placed under vacuum and backfilled with nitrogen 3 times. To the flask was added 3.0 mL of trifluoroacetic acid (final reaction concentration 0.3 M), and the resulting mixture was allowed to stir at room temperature until all of the L-serine had dissolved. The L-serine solution was then cooled to 0 °C in an ice-water bath under nitrogen for 30 min. In a separate vessel, the acyl chloride solution was allowed to cool to 0 °C in an ice-water bath under nitrogen for 30 min. The solution of acyl chloride was slowly added dropwise to the L-serine solution over 5 min, and this reaction mixture was allowed to warm to room temperature under an atmosphere of nitrogen for 16 h. After completion of the reaction, the reaction mixture was transferred to a beaker containing 20 mL of cold diethyl ether, causing precipitation of an off-white solid. The crude solid was filtered through a fine glass frit and washed with diethyl ether. To the crude solid was added 5 mL of diH_2O, producing a colorless solution. Saturated $NaHCO_3$ was added until the solution reached pH 5.5, causing an off-white solid to precipitate. The mother liquor was removed, and the crude material was recrystallized from boiling diH_2O to give **4** (60.0 mg, 0.241 mmol, 20% yield) as colorless, reflective crystals.

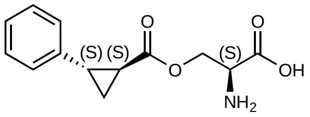

4

Data for *O*-((1*S*,2*S*)-2-phenylcyclopropane-1-carbonyl)-L-serine (**4**)

^{1}H NMR (500 MHz, acetic acid-d_4) δ 7.27 (t, *J* = 7.6 Hz, 2H), 7.19 (t, *J* = 7.3 Hz, 1H), 7.11 (d, *J* = 7.5 Hz, 2H), 4.73 – 4.54 (m, 2H), 4.36 (dd, *J* = 5.2, 2.9 Hz, 1H), 2.53 (ddd, *J* = 10.1, 6.7, 4.0 Hz, 1H), 1.95 (dt, *J* = 8.8, 4.7 Hz, 1H), 1.61 (dt, *J* = 9.6, 4.9 Hz, 1H), 1.46 – 1.36 (m, 1H).

^{13}C NMR (126 MHz, acetic acid-d_4) δ 174.70, 171.62, 140.55, 129.38, 127.47, 126.84, 63.86, 54.82, 27.49, 24.59, 18.02.

FTIR (neat, cm^{-1}) $\boldsymbol{\upsilon}_{\mathbf{max}}$: 3013 br (O–H), 1717 s (C=O), 1603 s (C=O)

HRMS (ESI): *m/z* for $C_{13}H_{16}NO_4^+$ $[M+H]^+$ calcd.: 250.1074, found: 250.1076.

T_r (HPLC-MS): 30.1 min

The absolute configuration of the product is determined by the correlation to the known absolute configuration of the reactants.

Synthesis of standard for the product (aza-phtha, compound 5, mixture of C1 and C2 diastereomers in Fig. 3d) of the reaction of azaserine with phthalan

Synthesis of *tert*-butyl *N*-(*tert*-butoxycarbonyl)-*O*-(2-(1,3-dihydroisobenzofuran-1-yl)acetyl)-L-serinate (S13)

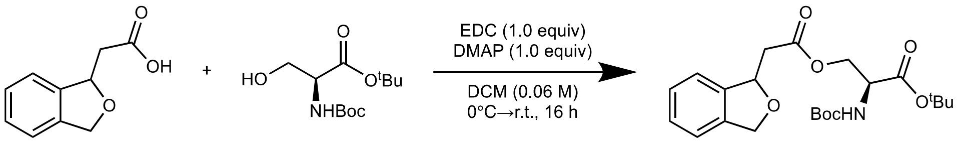

S13

To a stirred solution of 2-(1,3-dihydroisobenzofuran-1-yl)acetic acid (222 mg, 1.25 mmol) in anhydrous dichloromethane (20 mL) at 0 °C under nitrogen was added EDC (239 mg, 1.25 mmol) and the resulting reaction mixture was continued to stir for 30 mins. Then *tert*-butyl (*tert*-butoxycarbonyl)-L-serinate (326 mg, 1.25 mmol) was added, followed by the addition of DMAP (152 mg, 1.25 mmol). The ice bath was removed, and the reaction mixture was stirred at room temperature for 16 h. After evaporation of the solvent under reduced pressure, the residue was subjected to purification by column chromatography to give 409 mg of compound **S13** (*tert*-butyl *N*-(*tert*-butoxycarbonyl)-*O*-(2-(1,3-dihydroisobenzofuran-1-yl)acetyl)-L-serinate) as a mixture of two inseparable diastereomers (coelute from flash chromatography) in 78% yield.

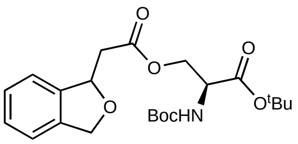

S13

Data of *tert*-butyl *N*-(*tert*-butoxycarbonyl)-*O*-(2-(1,3-dihydroisobenzofuran-1-yl)acetyl)-L-serinate (**S13**):

^{1}**H-NMR** (600 MHz, $CDCl_3$) **δ** (ppm) 7.32-7.27 (m, 4H), 7.25-7.22 (m, 2H), 7.19-7.16 (m, 2H), 5.65-5.60 (m, 2H), 5.50-5.45 (m, 1H), 5.37-5.32 (m, 1H), 5.17-5.12 (m, 2H), 5.09-5.05 (dd, 2H, J = 12.2, 6.4 Hz), 4.50-4.37 (m, 6H), 2.86-2.81 (dd, 1H, J = 17.2, 4.6 Hz), 2.83-2.78 (dd, 1H, J = 17.2, 4.6 Hz), 2.74-2.68 (m, 2H), 1.50-1.43 (m, 36H).

^{13}**C NMR** (151 MHz, $CDCl_3$) **δ** 170.29, 170.17, 168.78, 168.71, 155.24, 155.30, 140.45, 140.40, 139.14, 139.12, 127.98, 127.53, 121.15 (2C), 121.10, 121.08, 82.67, 82.65, 80.22, 80.12, 79.99(2C), 79.94(2C), 72.68, 72.64, 64.98, 64.94, 53.46, 53.39, 41.39, 41.37, 28.3(6C), 27.9 (2C).

HRMS (ESI) $[M+Na]^+$ calculated for $C_{22}H_{31}NO_7Na$: 444.1993, found 444.1996.

Synthesis of O-(2-(1,3-dihydroisobenzofuran-1-yl)acetyl)-L-serine (5)

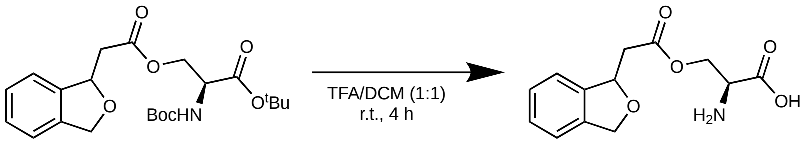

S13 5

To a stirred solution of *tert*-butyl *N*-(*tert*-butoxycarbonyl)-*O*-(2-(1,3-dihydroisobenzofuran-1-yl)acetyl)-L-serinate (**S13**, 15 mg, 0.036 mmol) in dichloromethane (1.0 mL) was added trifluoroacetic acid (1 mL, 13 mmol) and the resulting reaction mixture was continued to stir for 4 h at room temperature. After evaporation of the solvent under reduced pressure, the residue (13 mg) was obtained in 99% yield as a mixture of two diastereomers (with 1:1 ratio), which could not be separated by flash column chromatography. The compounds **5** were used directly without further purification.

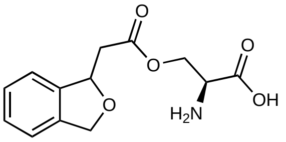

5

Data of *O*-(2-(1,3-dihydroisobenzofuran-1-yl)acetyl)-L-serine (**5**):

^{1}**H-NMR** (600 MHz, CD_3OD) **δ** (ppm) 7.35-7.18 (m, 8H), 5.62-5.56 (m, 2H), 5.12-5.05 (dd, 2H, J = 12.0, 2.4 Hz), 5.02-4.98 (d, 2H, J = 2.4 Hz), 4.60-4.56 (dd, 2H, J = 12.4, 3.3 Hz), 4.55-4.50 (ddd, 2H, J = 12.3, 4.8, 1.4 Hz), 4.35-4.31 (m, 2H), 2.97-2.92 (dd, 2H, J = 15.8, 4.1 Hz), 2.72-2.66 (ddd, 2H, J = 15.8, 8.6, 0.7 Hz).

^{13}**C NMR** (151 MHz, CD_3OD) **δ** 170.2, 170.1, 167.6 (2C), 140.0, 138.7, 127.8 (2C), 127.3 (2C), 120.83, 120.82, 120.79, 120.78, 80.2, 80.1, 72.2 (2C), 61.55, 61.54, 52.0 (2C), 40.6, 40.5.

HRMS (ESI) $[M+H]^+$ calculated for $C_{13}H_{16}NO_5$: 266.1023, found 266.1025.

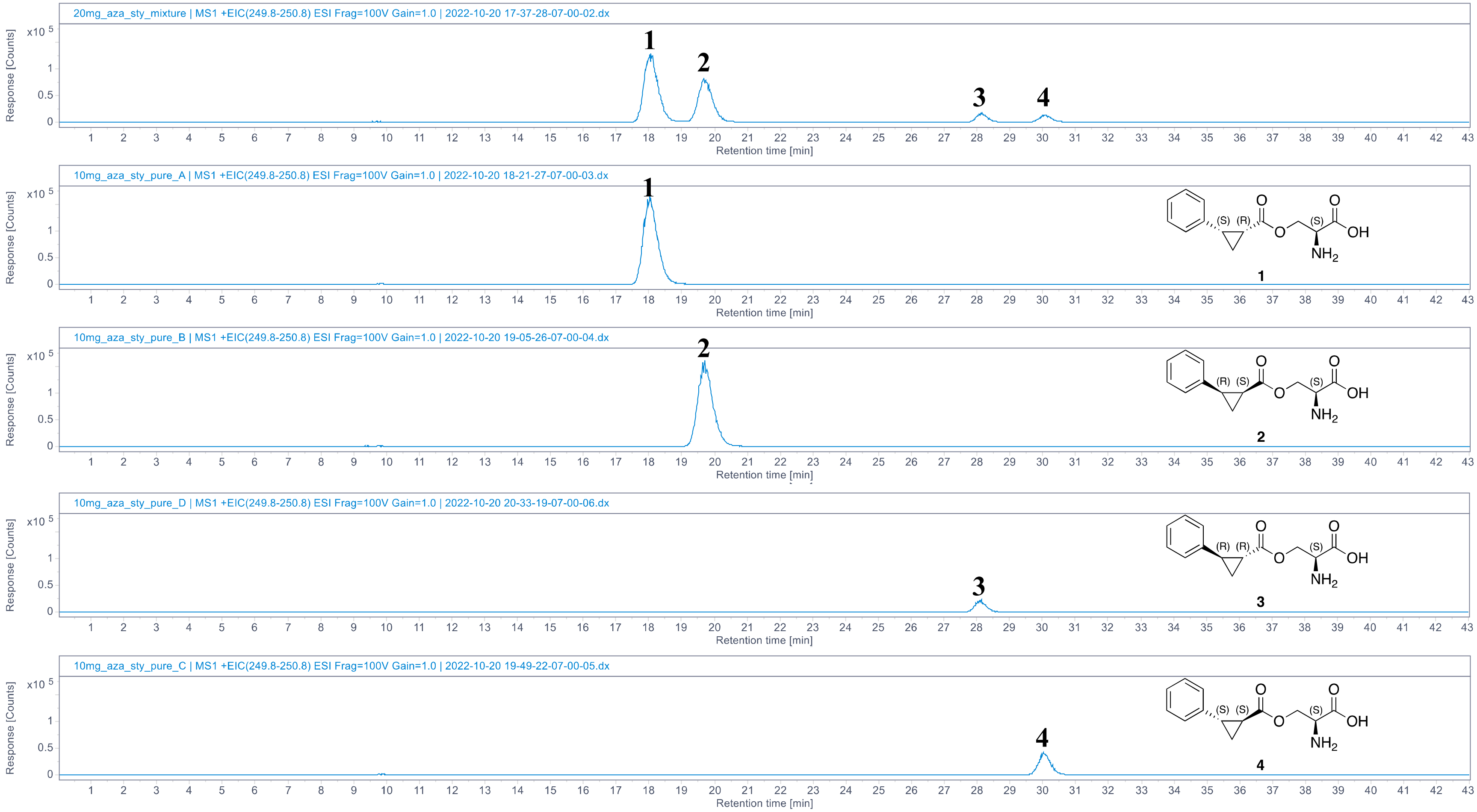

Supplementary Fig. 1 | LC-MS traces of authentic aza-sty products. Extracted ion chromatograms for synthetic aza-sty products (**1**, **2**, **3**, **4** correspond to **P1, P2, P3, P4** peaks in the manuscript) (*m/z* $[M+H]^+$ = 249.8-250.8) by standard HPLC-MS method.

NMR Spectra

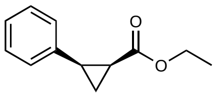

(±)

S1

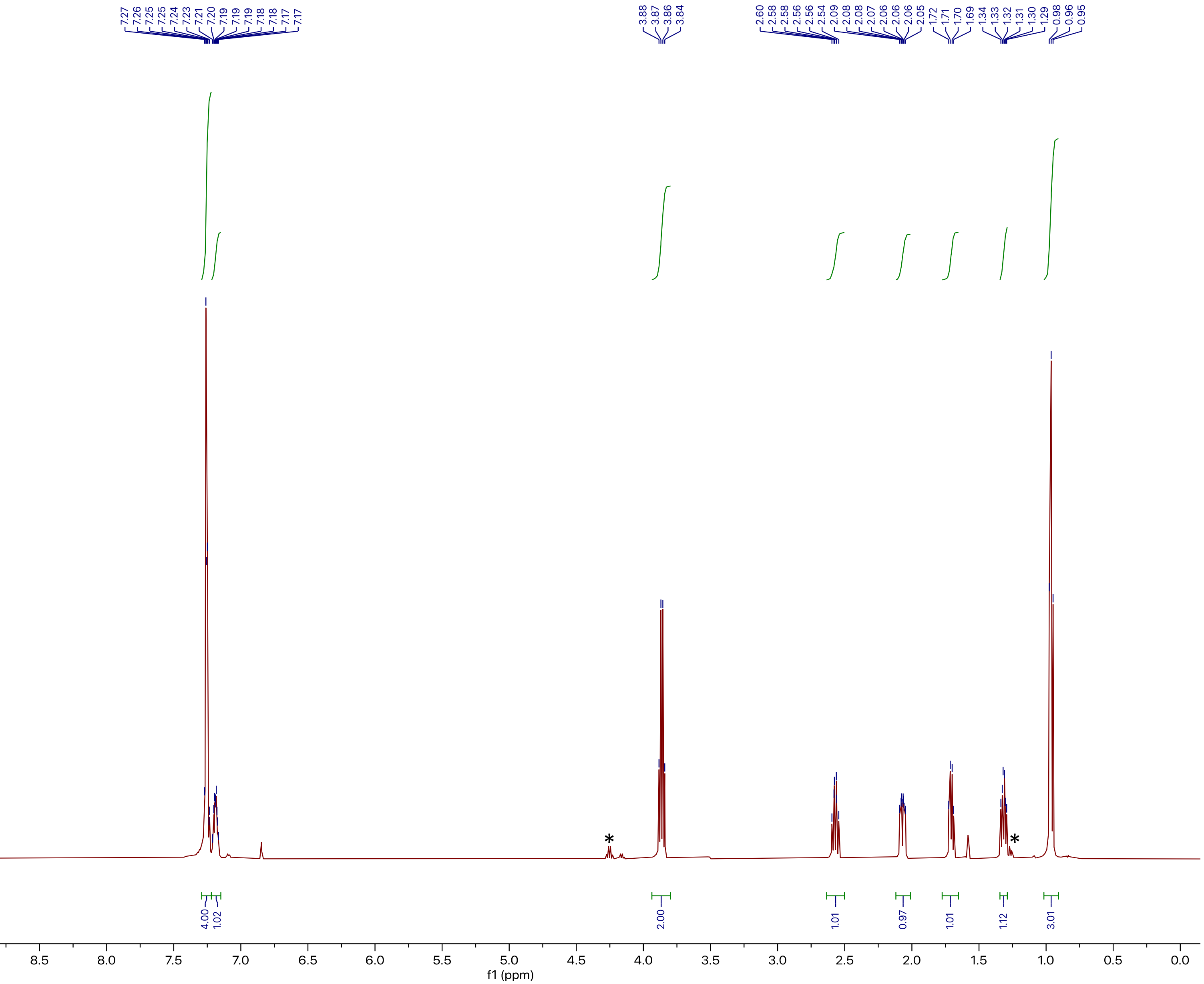

Supplementary Fig. 2 | ^{1}H-NMR (500 MHz, $CDCl_3$) of S1. *Impurity resulting from diastereomer **S2**, which is removed in later steps of the sequence.

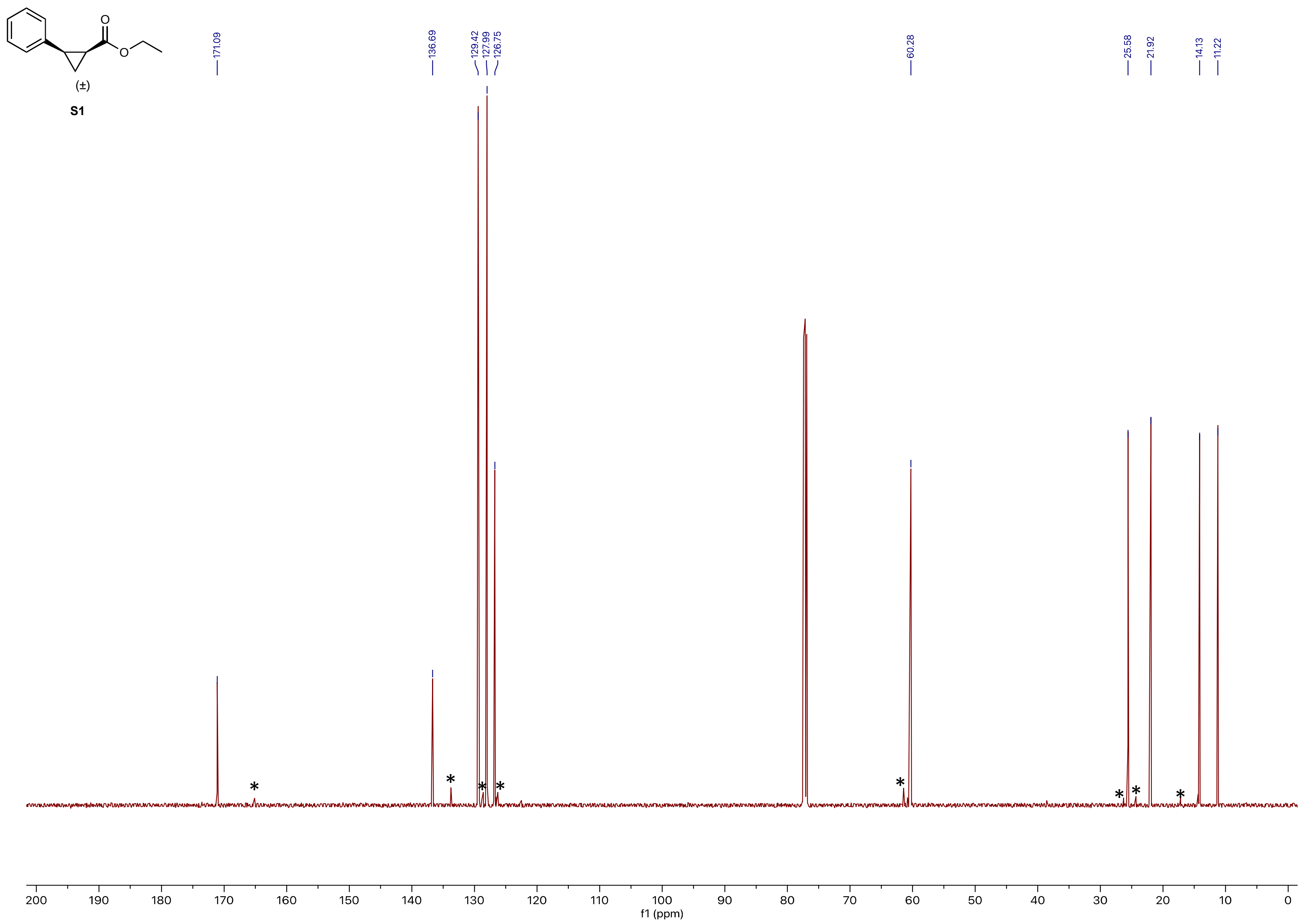

Supplementary Fig. 3 | ^{13}C-NMR (126 MHz, $CDCl_3$) of S1. *Impurity resulting from diastereomer **S2**, which is removed in later steps of the sequence.

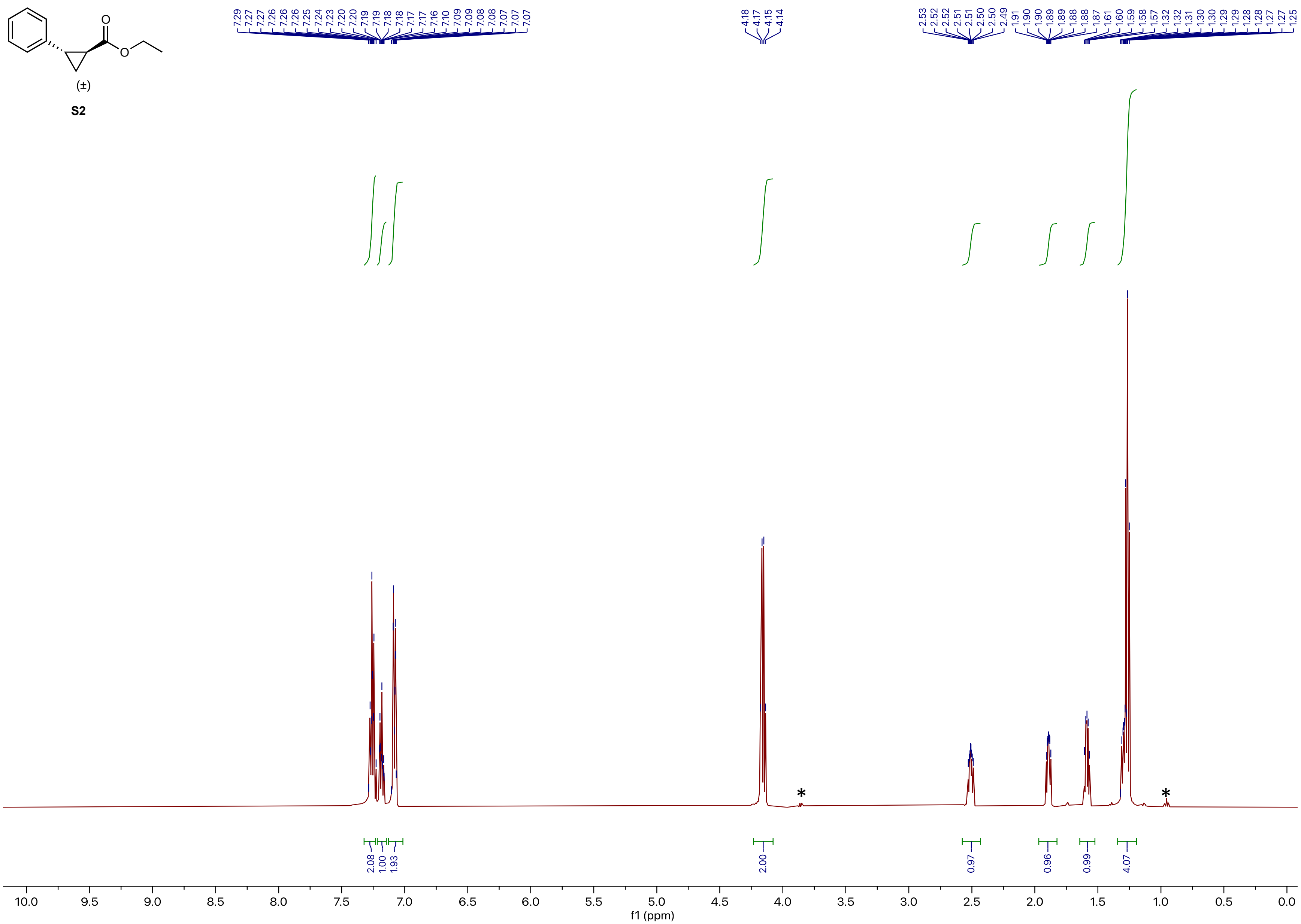

Supplementary Fig. 4 | ^{1}H-NMR (500 MHz, CDCl$_3$) of S2. *Impurity resulting from diastereomer **S1**, which is removed in later steps of the sequence.

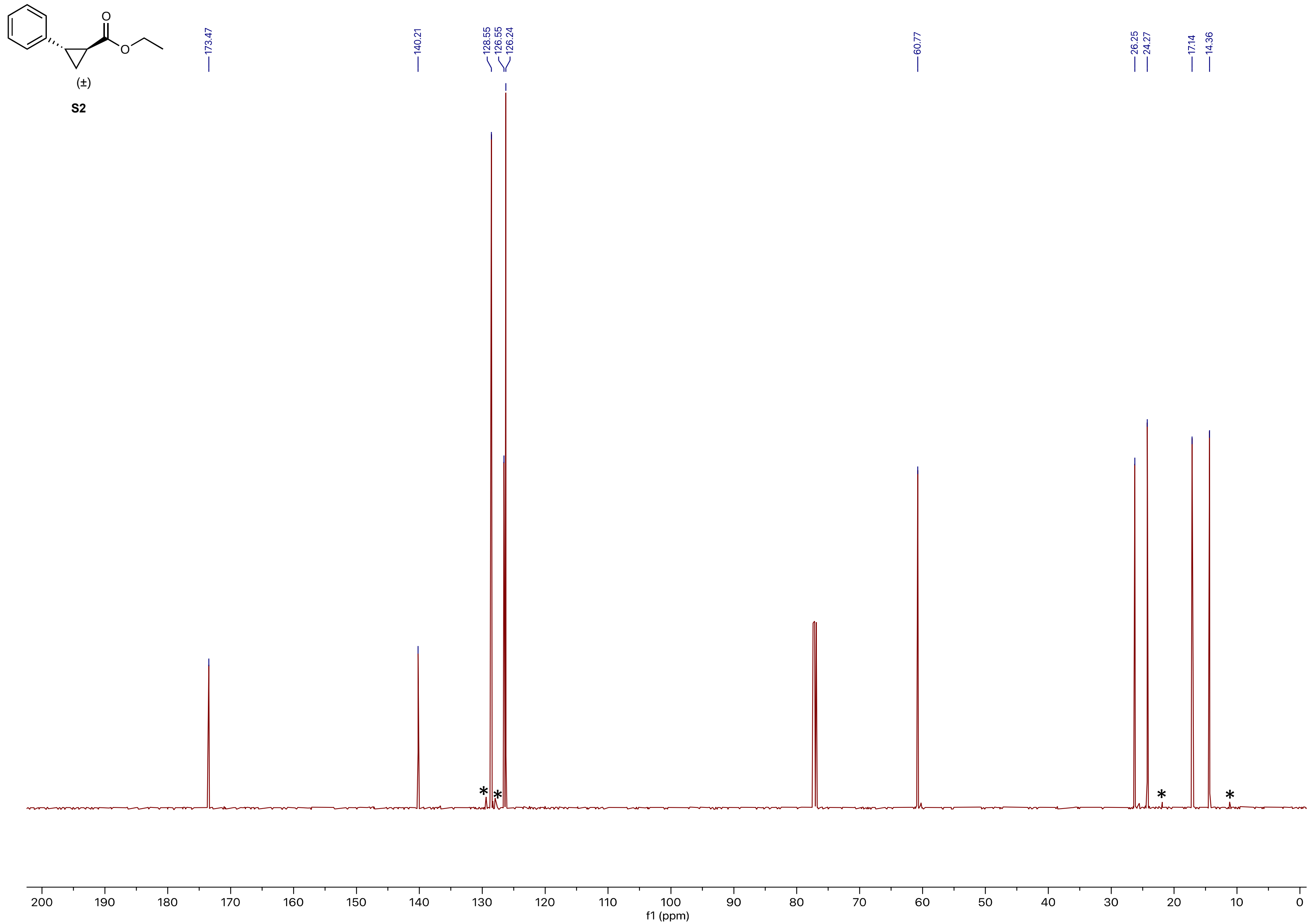

Supplementary Fig. 5 | ^{13}C-NMR (126 MHz, $CDCl_3$) of S2. *Impurity resulting from diastereomer **S1**, which is removed in later steps of the sequence.

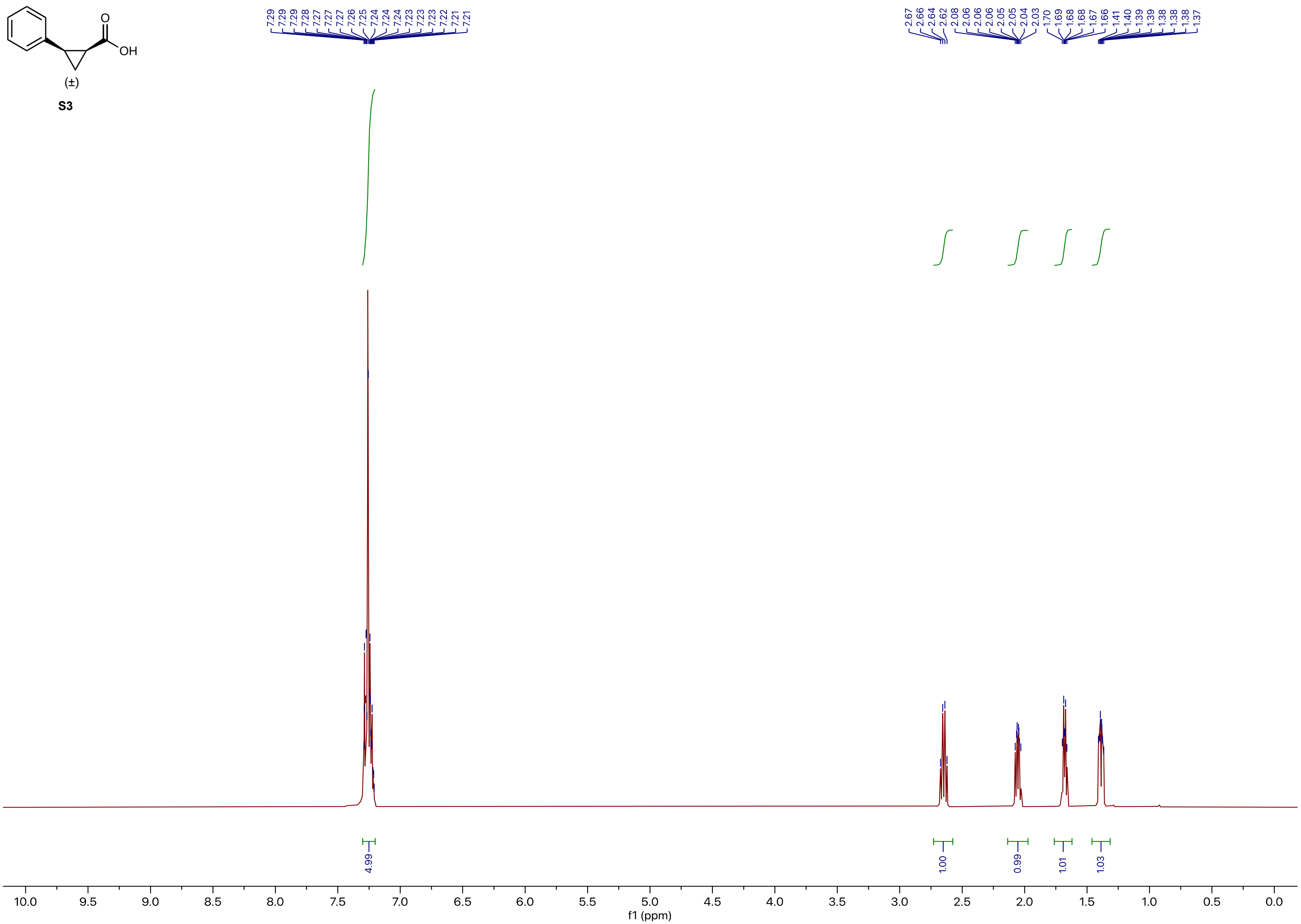

Supplementary Fig. 6 | ^{1}H-NMR (500 MHz, $CDCl_3$) of S3.

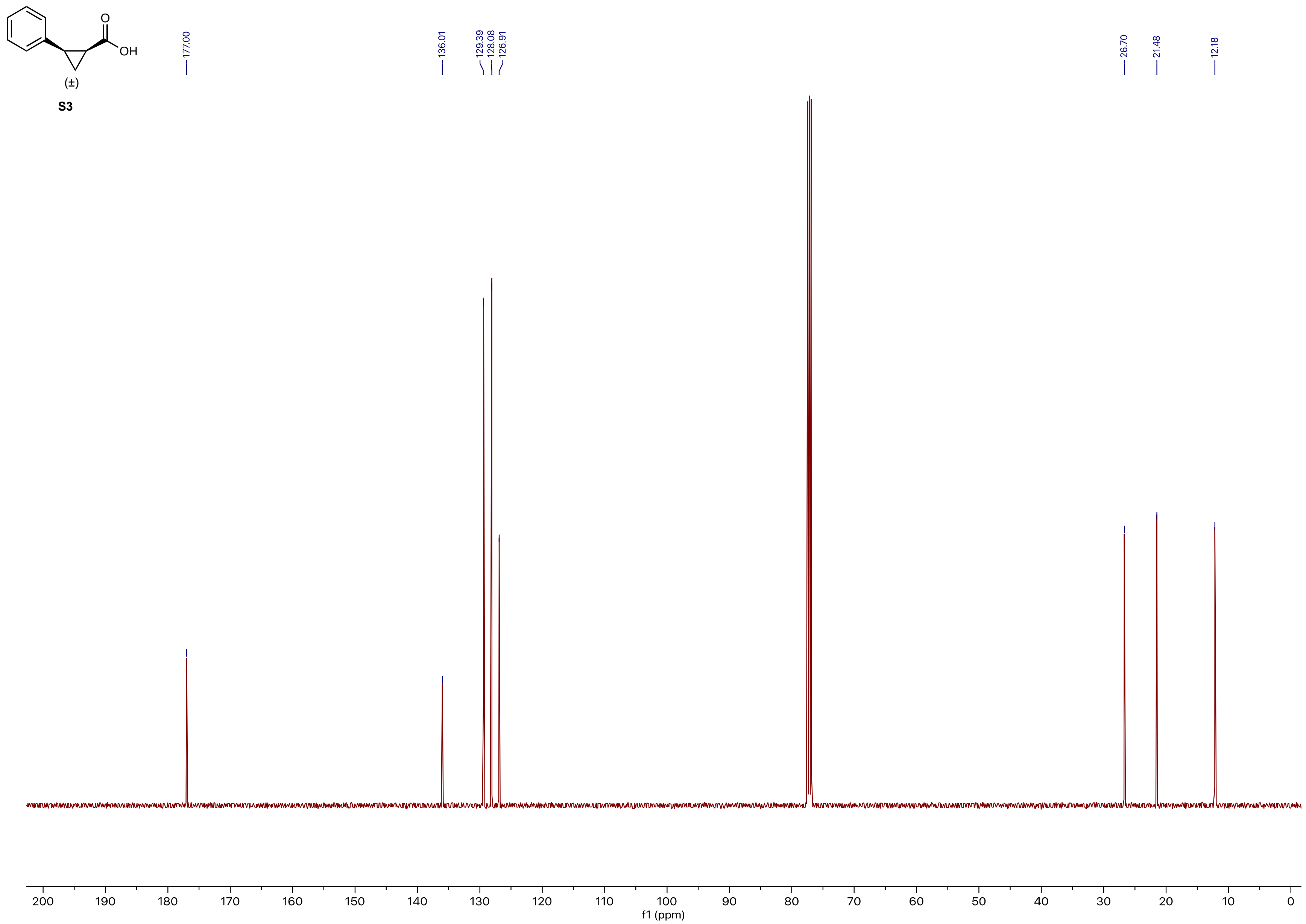

Supplementary Fig. 7 | ^{13}C-NMR (126 MHz, $CDCl_3$) of S3.

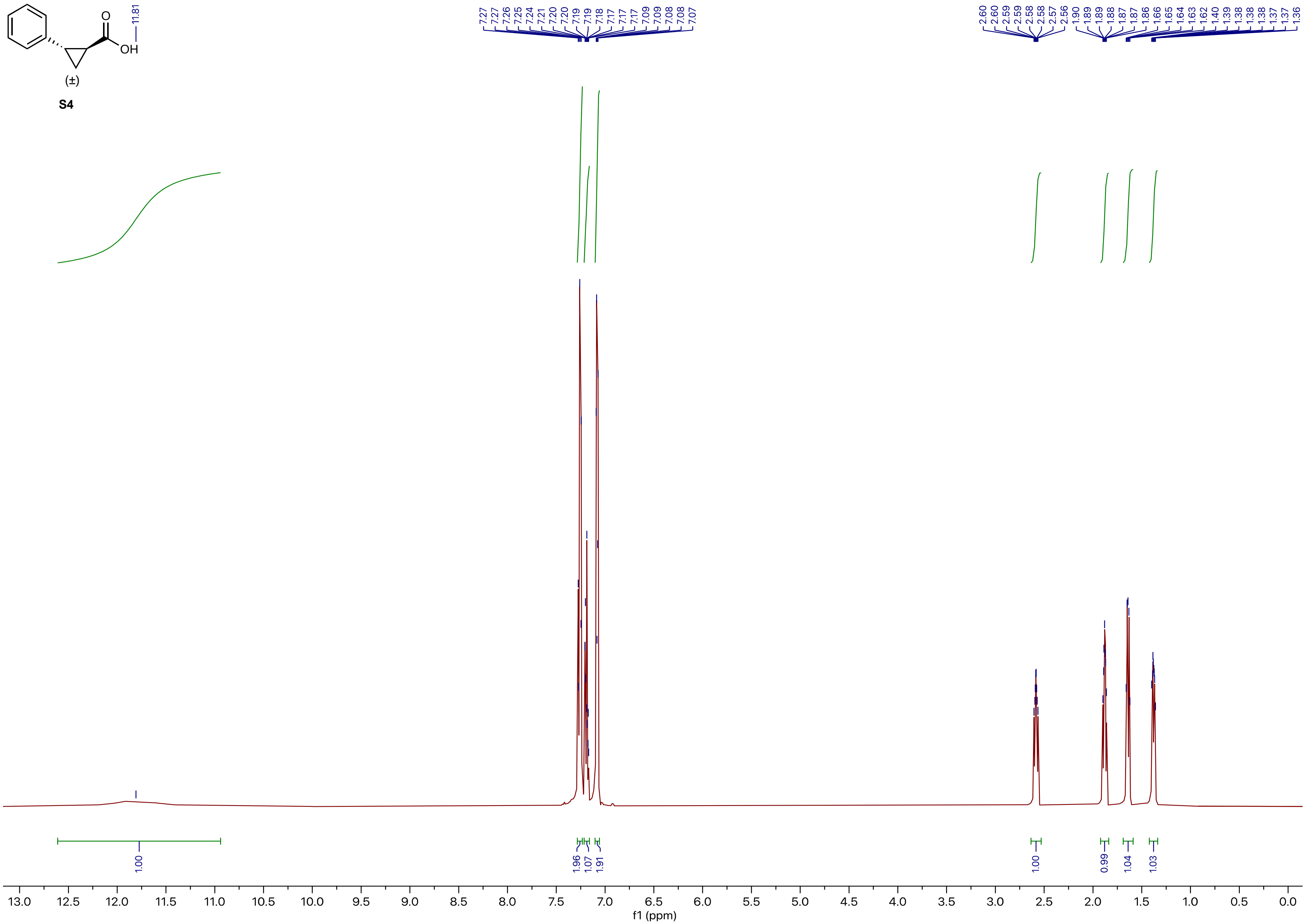

Supplementary Fig. 8 | ^{1}H-NMR (500 MHz, $CDCl_3$) of S4.

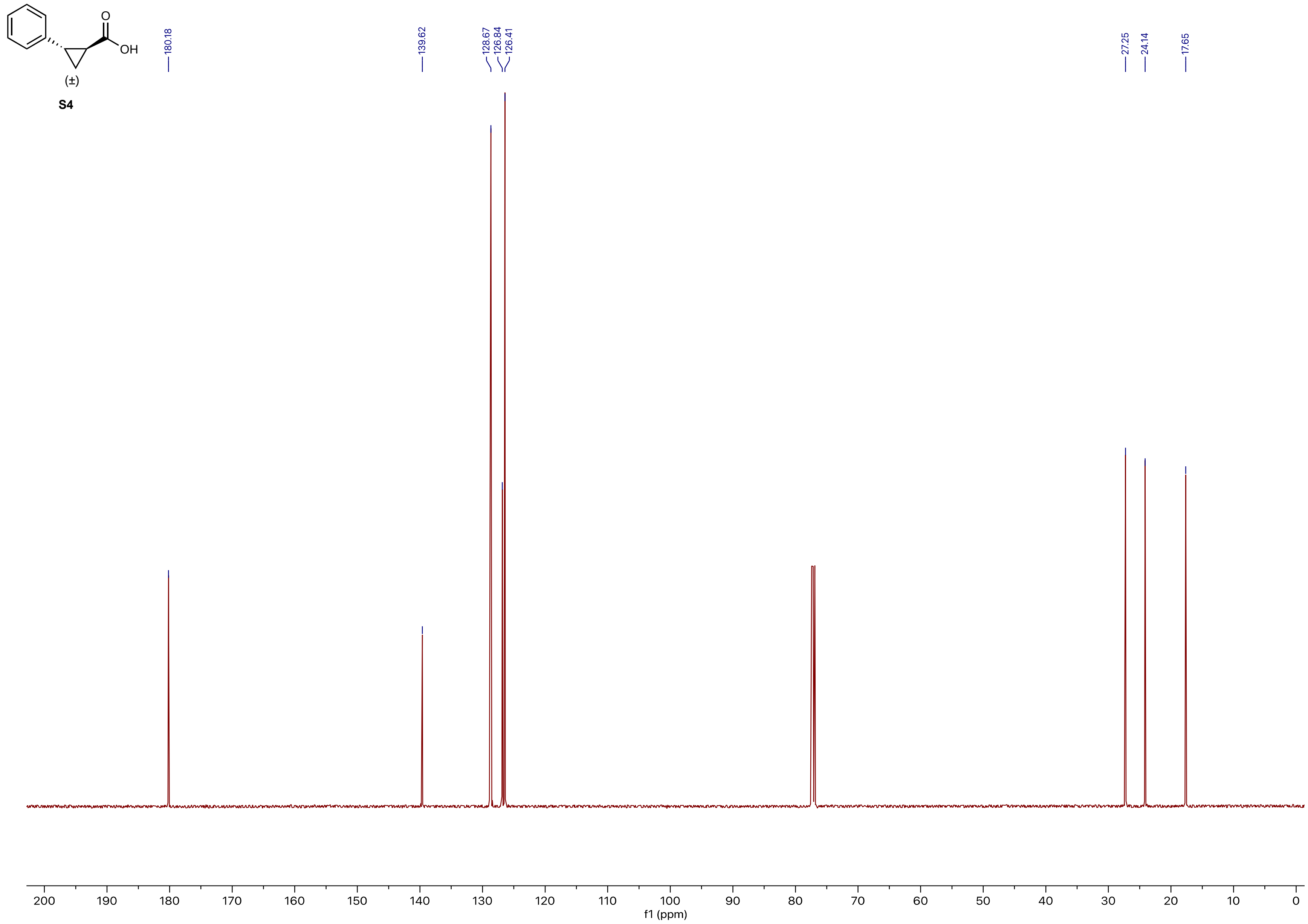

Supplementary Fig. 9 | ^{13}C-NMR (126 MHz, $CDCl_3$) of S4.

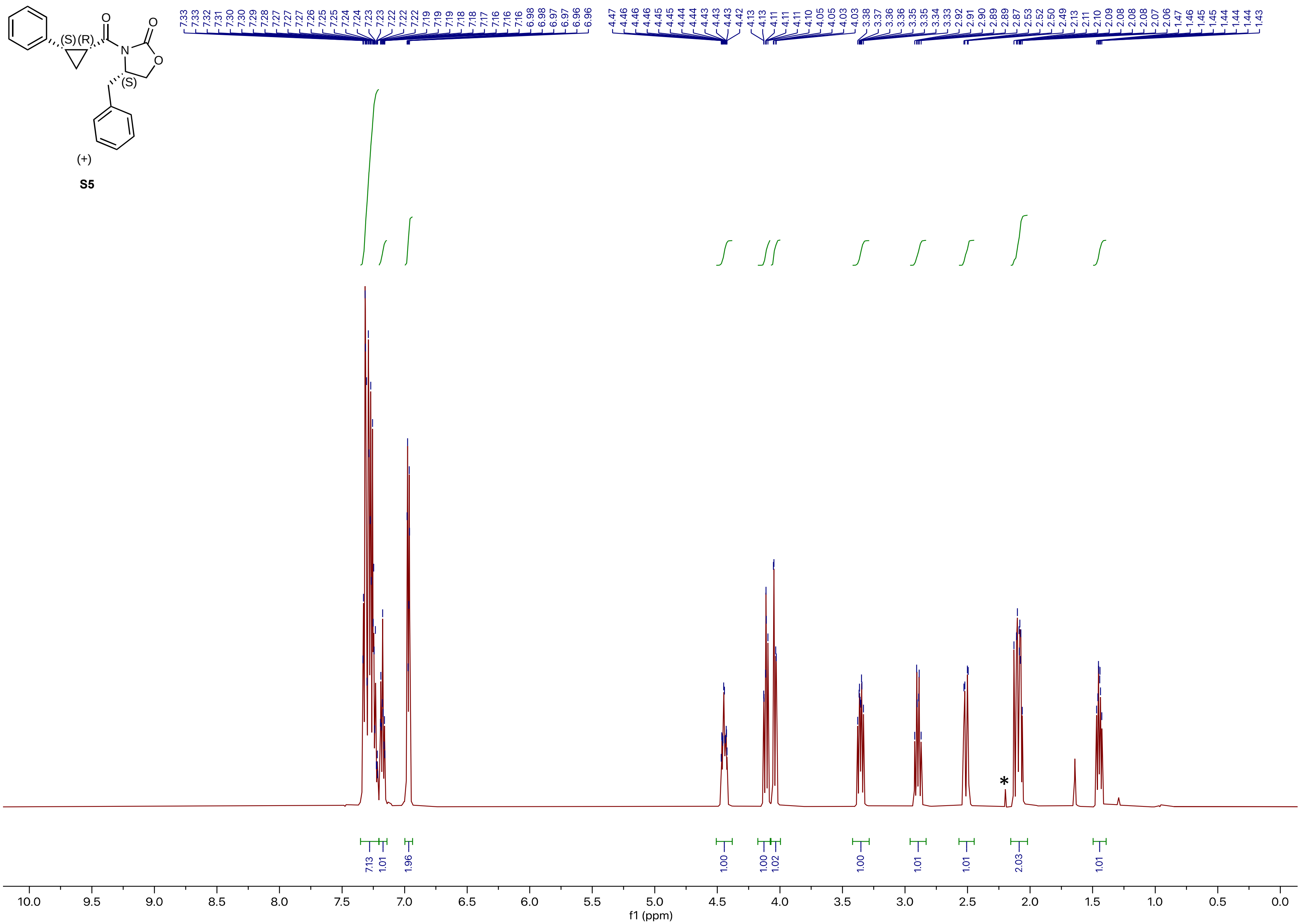

Supplementary Fig. 10 | ^{1}H-NMR (500 MHz, $CDCl_3$) of S5. *Impurity the result of acetone.

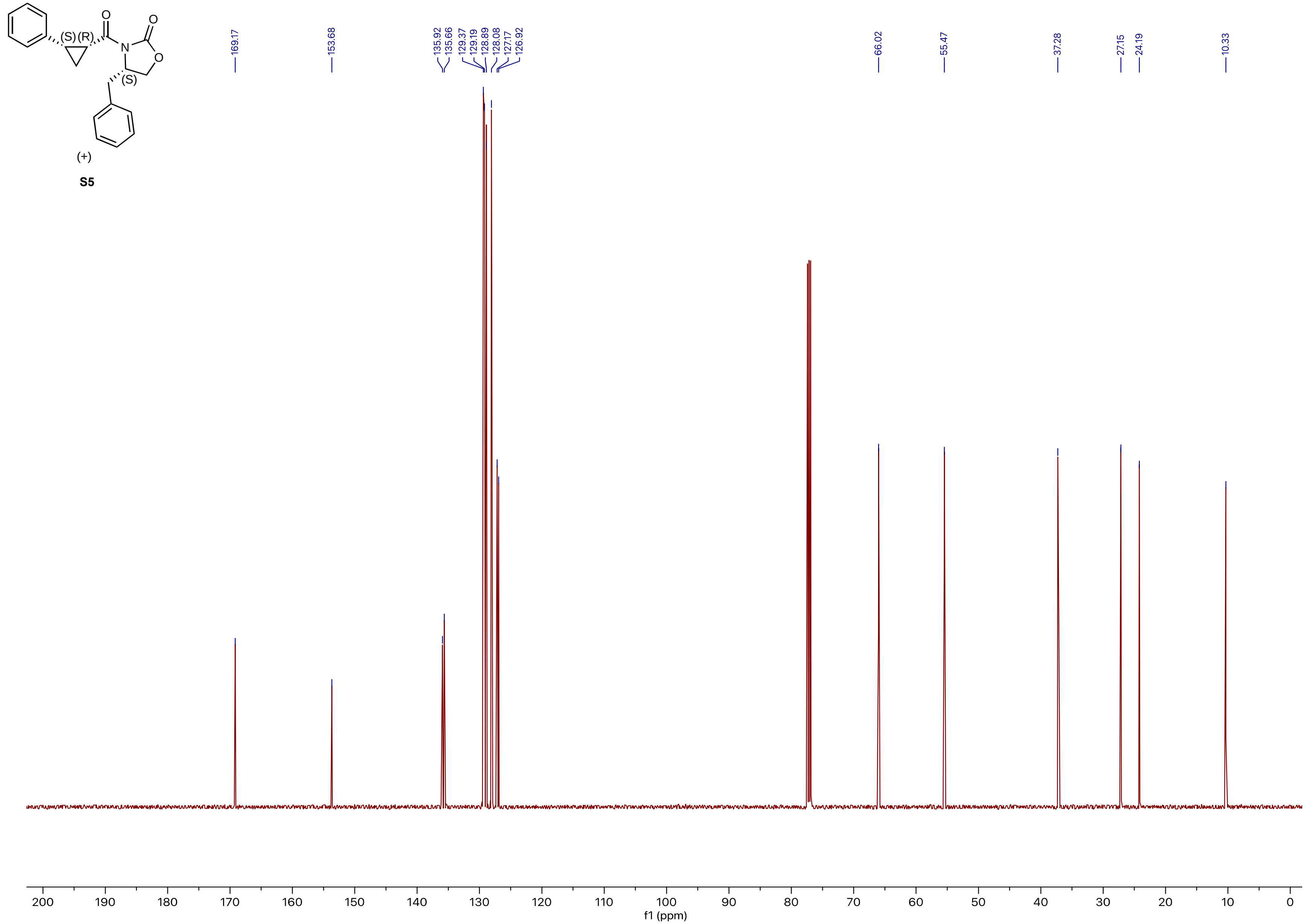

Supplementary Fig. 11 | ^{13}C-NMR (126 MHz, $CDCl_3$) of S5.

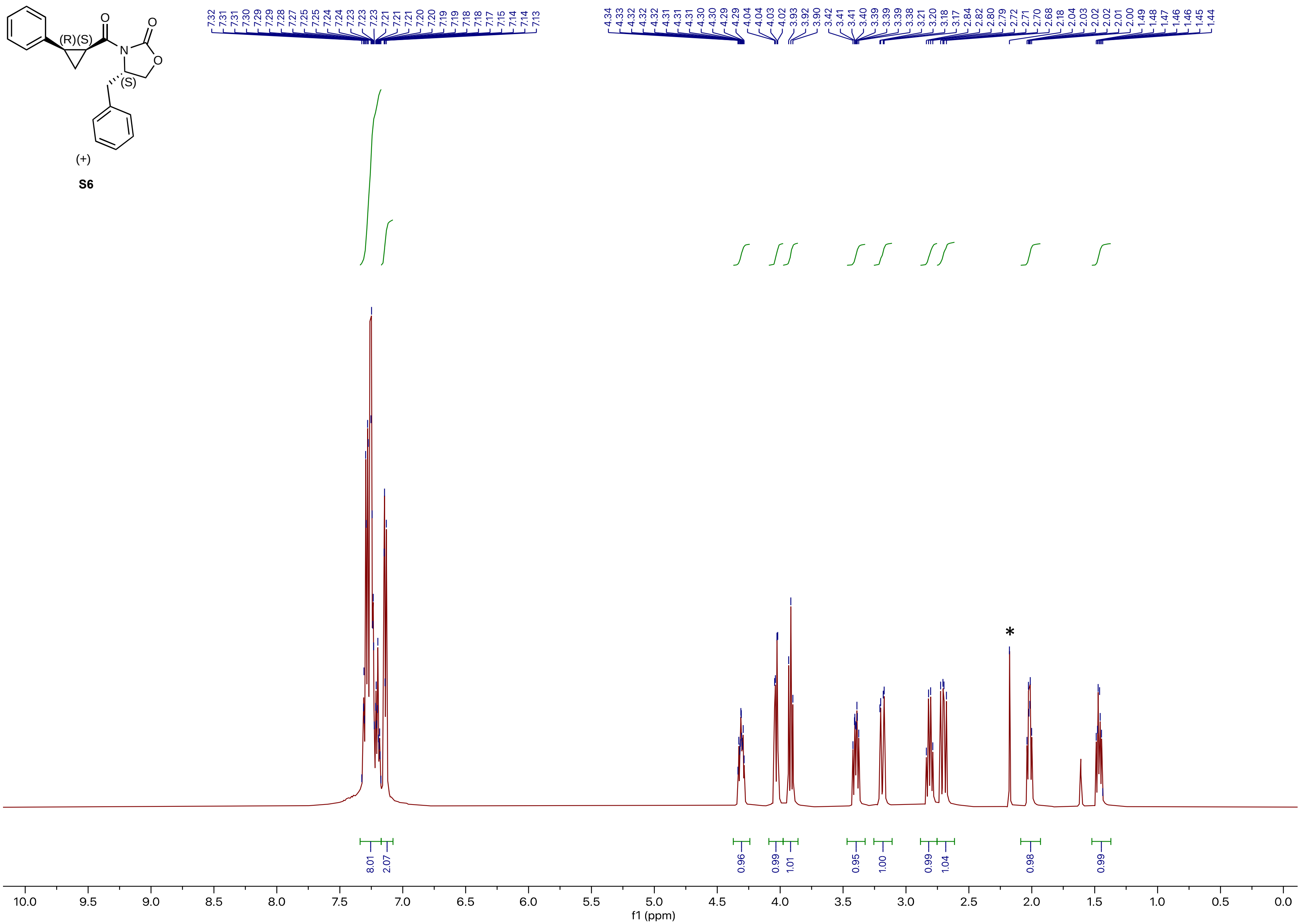

Supplementary Fig. 12 | ¹H-NMR (500 MHz, CDCl₃) of S6. *Impurity the result of acetone.

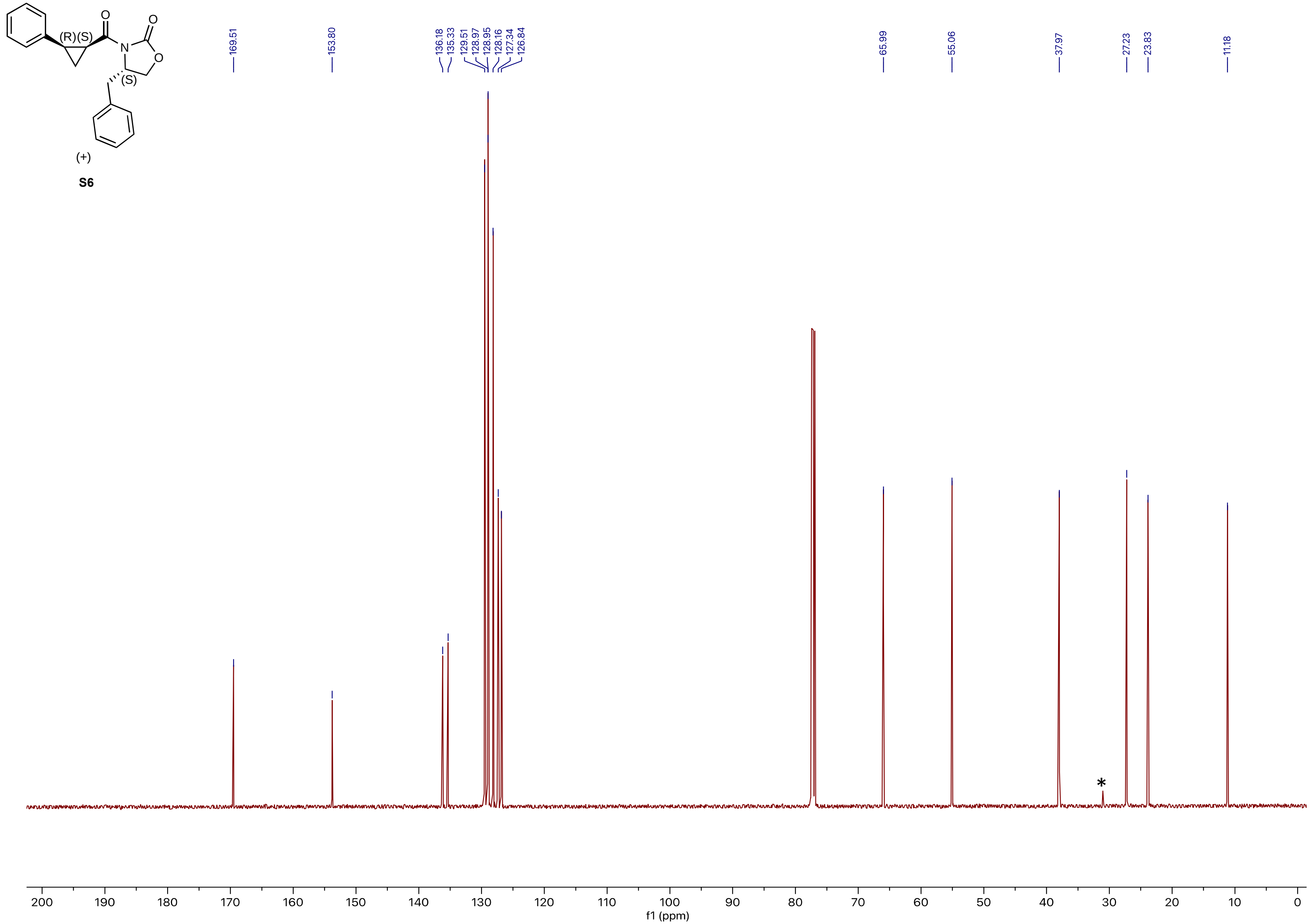

Supplementary Fig. 13 | ^{13}C-NMR (126 MHz, $CDCl_3$) of S6. *Impurity the result of acetone.

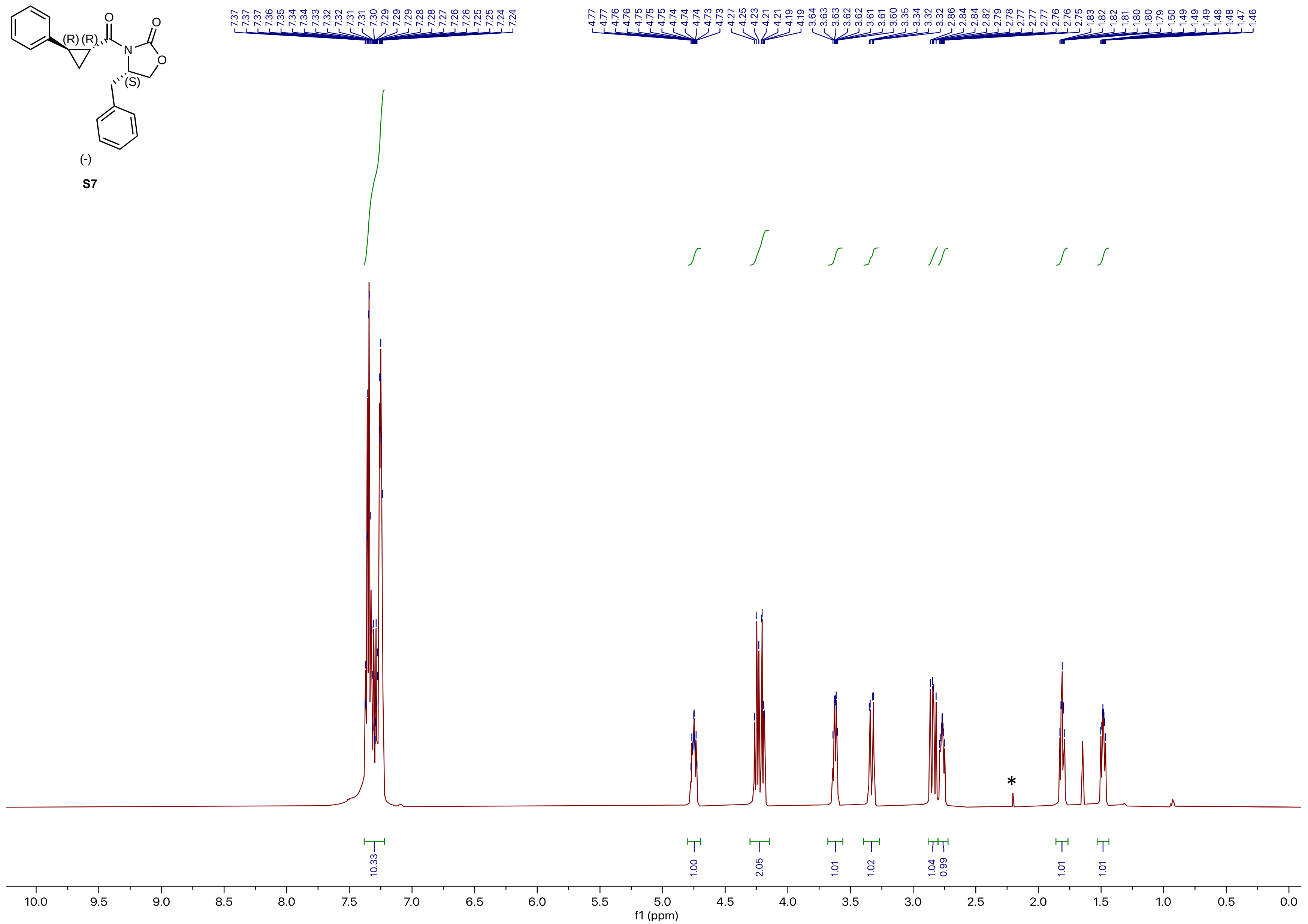

Supplementary Fig. 14 | ^{1}H-NMR (500 MHz, $CDCl_3$) of S7. *Impurity the result of acetone.

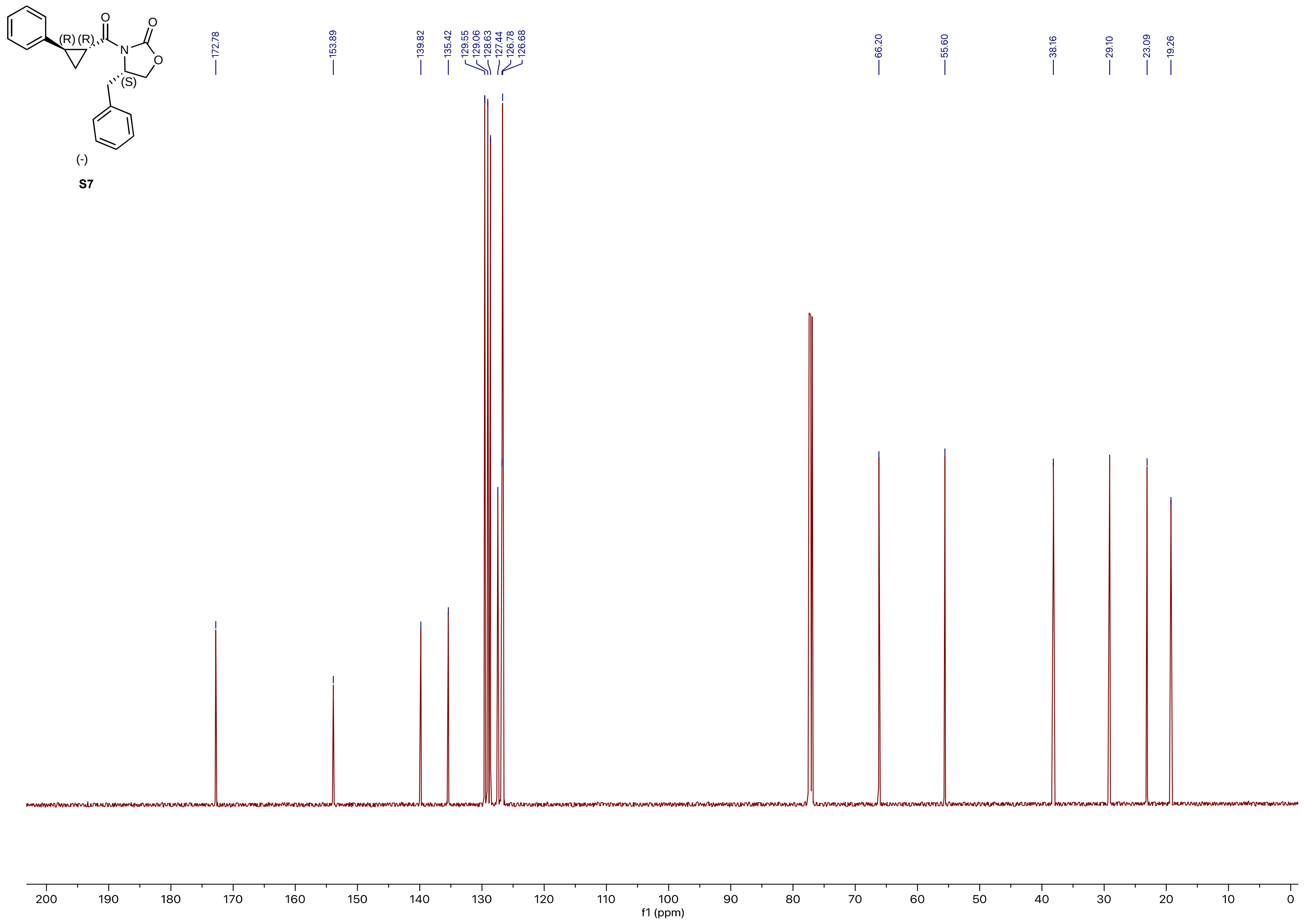

Supplementary Fig. 15 | ^{13}C-NMR (126 MHz, $CDCl_3$) of S7.

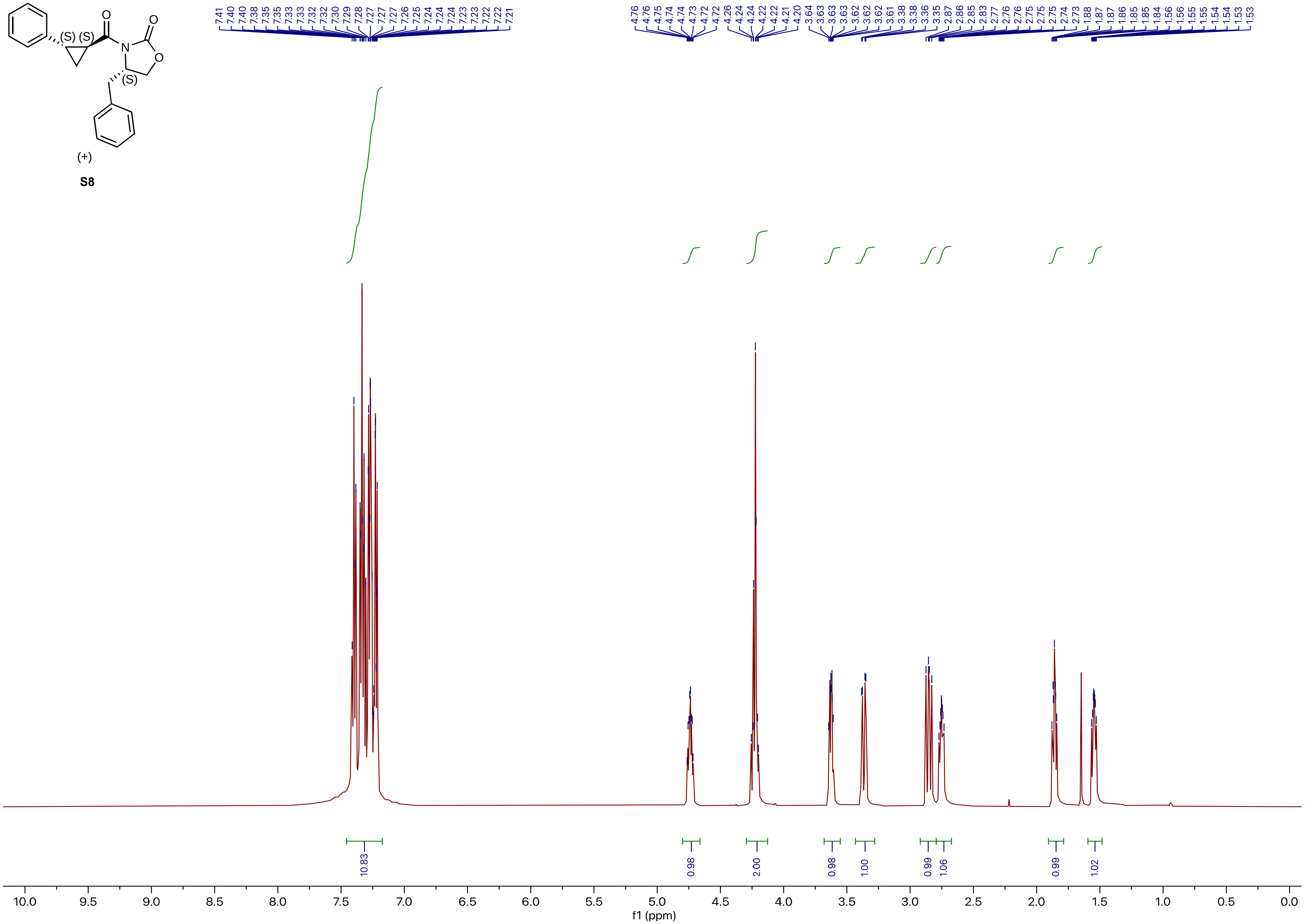

Supplementary Fig. 16 | ^{1}H-NMR (500 MHz, $CDCl_3$) of S8.

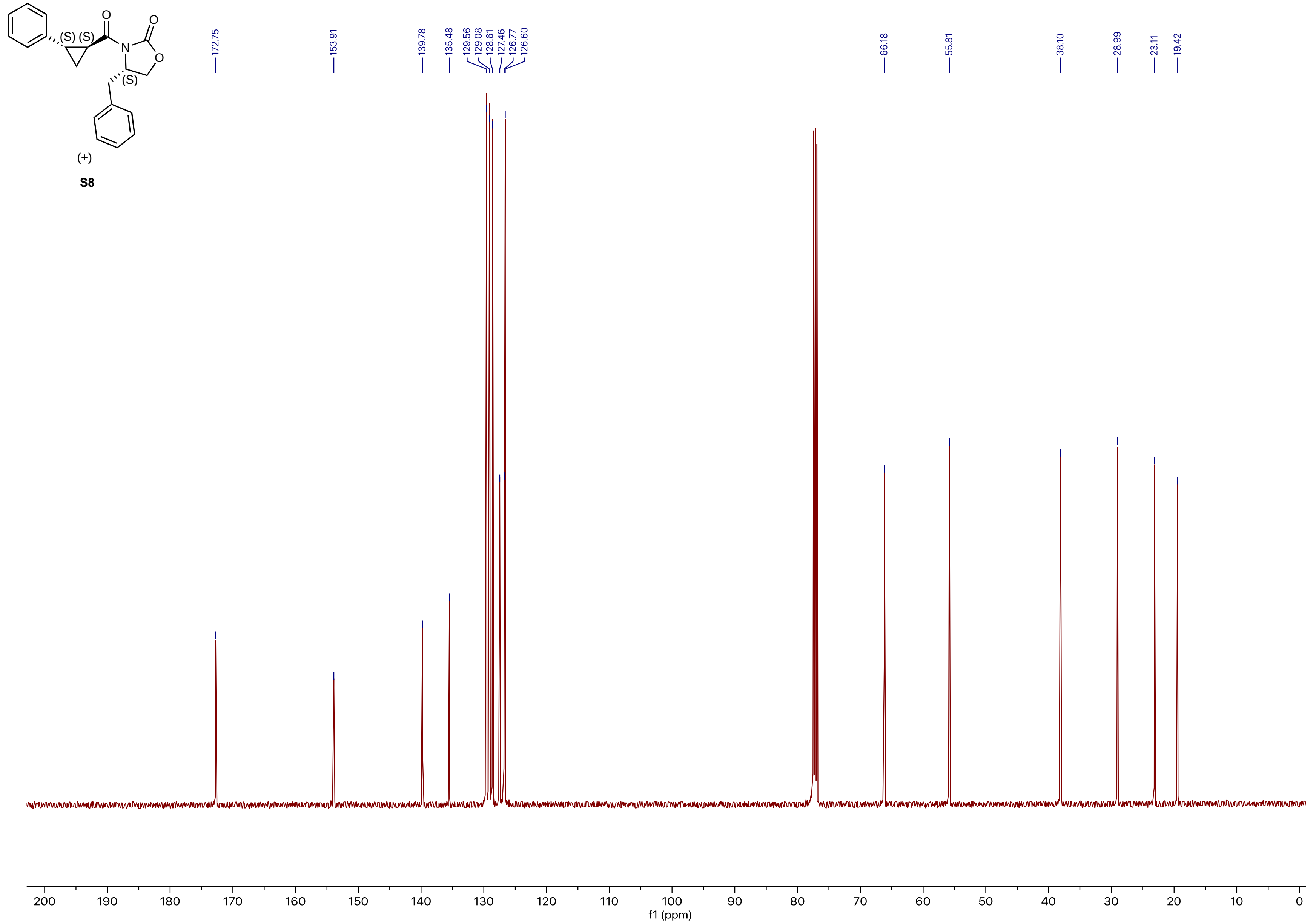

Supplementary Fig. 17 | ^{13}C-NMR (126 MHz, CDCl$_3$) of S8.

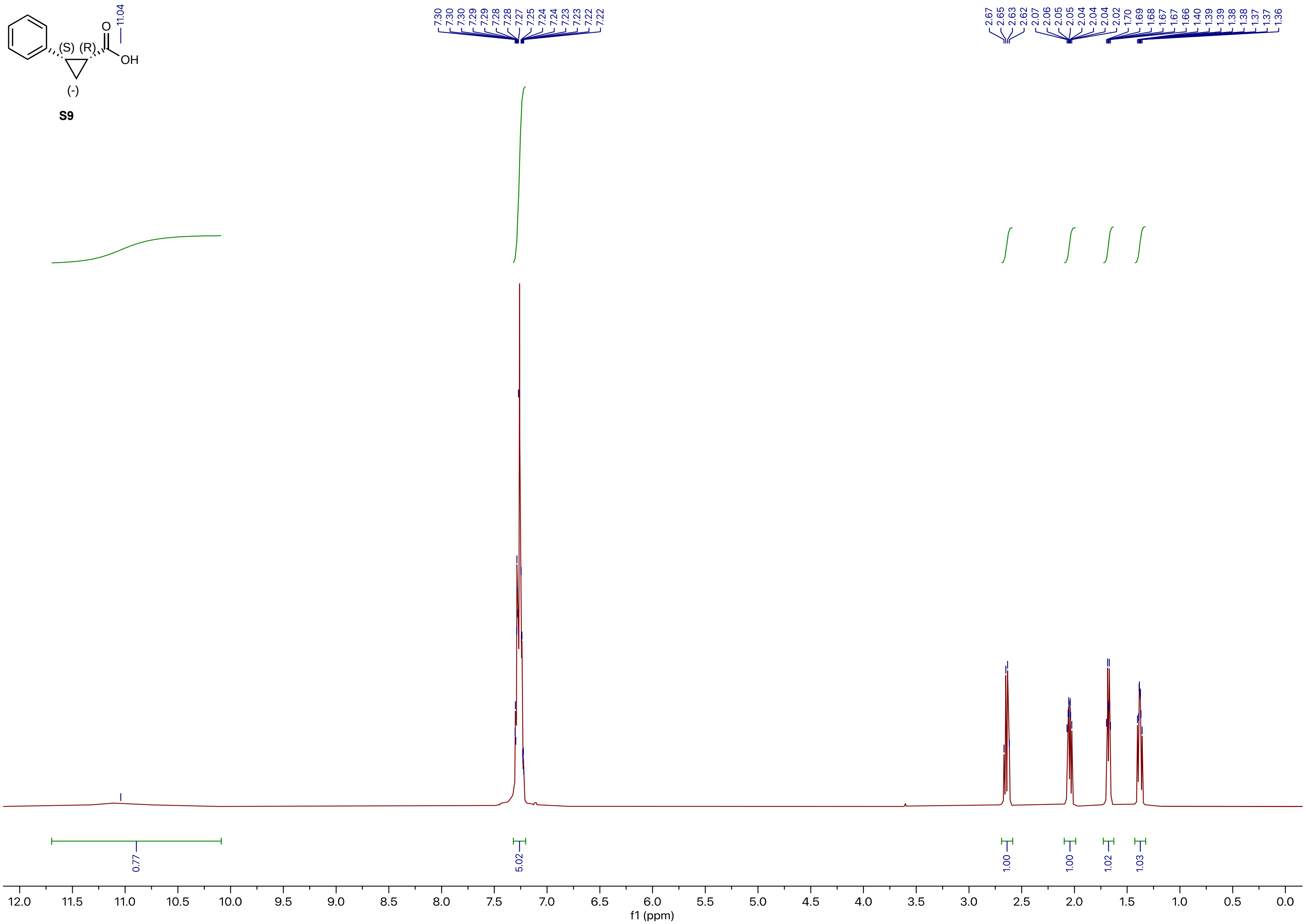

Supplementary Fig. 18 | ^{1}H-NMR (500 MHz, $CDCl_3$) of S9.

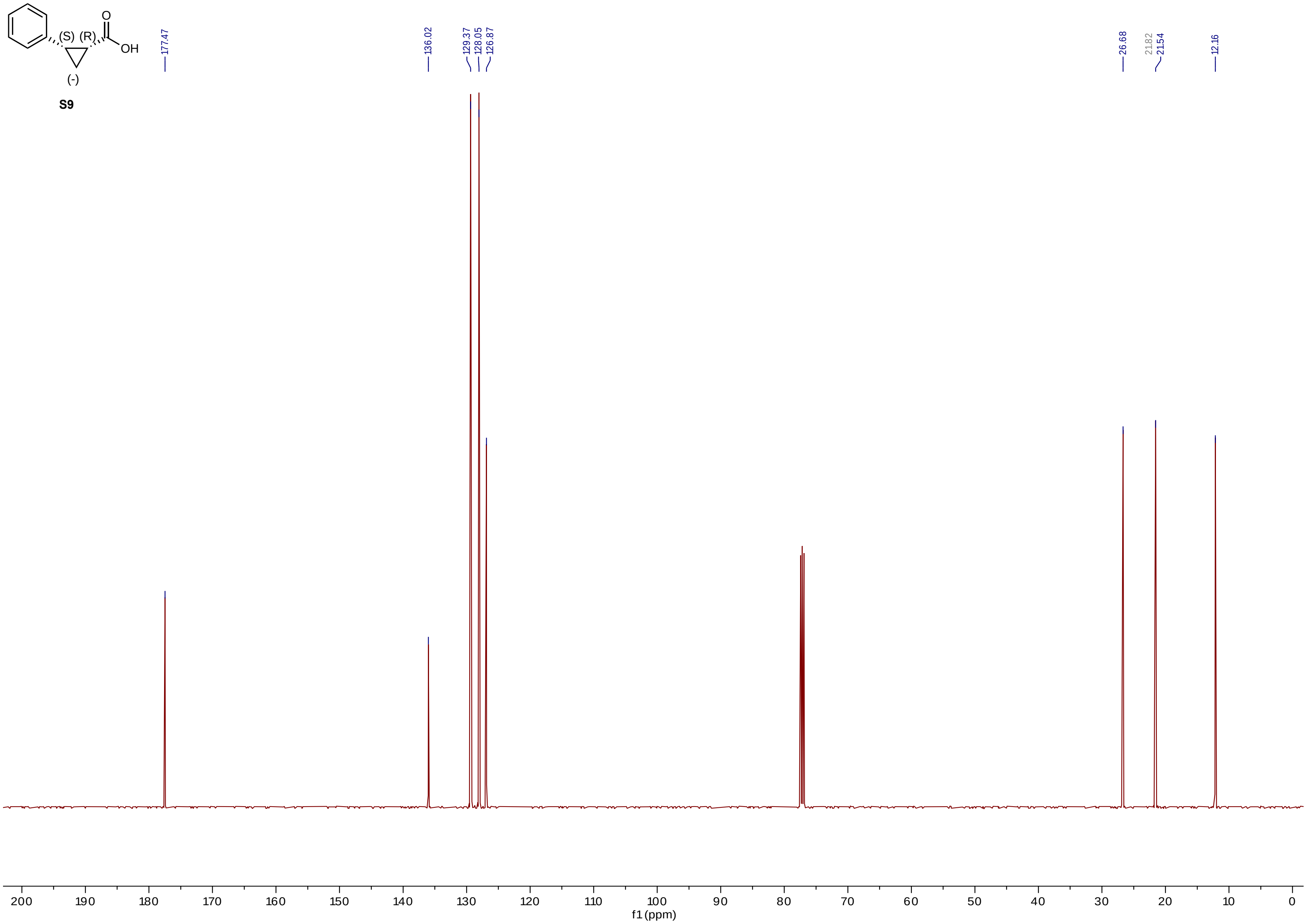

Supplementary Fig. 19 | ^{13}C-NMR (126 MHz, $CDCl_3$) of S9.

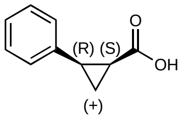

S10

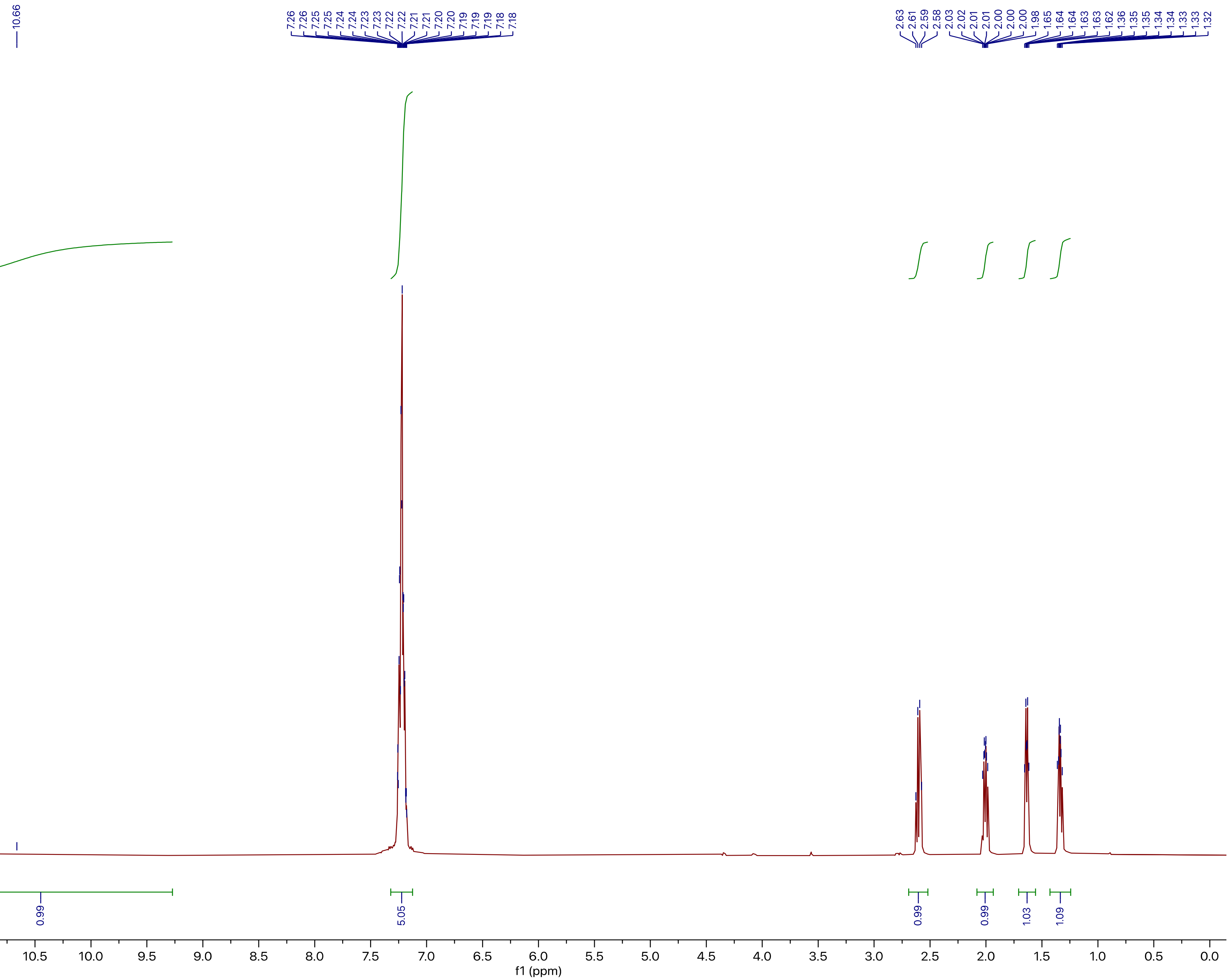

Supplementary Fig. 20 | ^{1}H-NMR (500 MHz, $CDCl_3$) of S10.

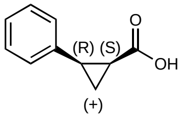

S10

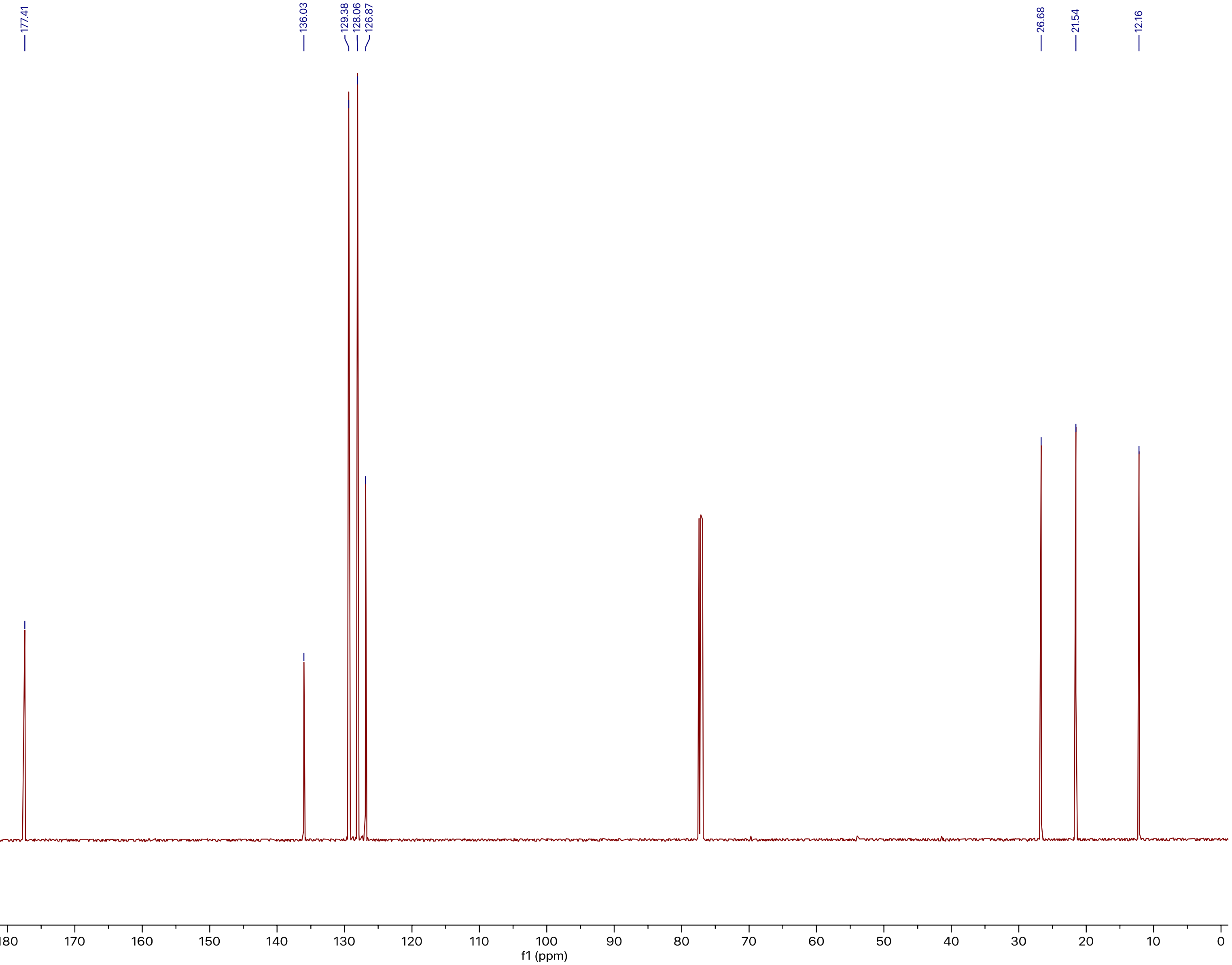

Supplementary Fig. 21 | ^{13}C-NMR (126 MHz, CDCl$_3$) of S10.

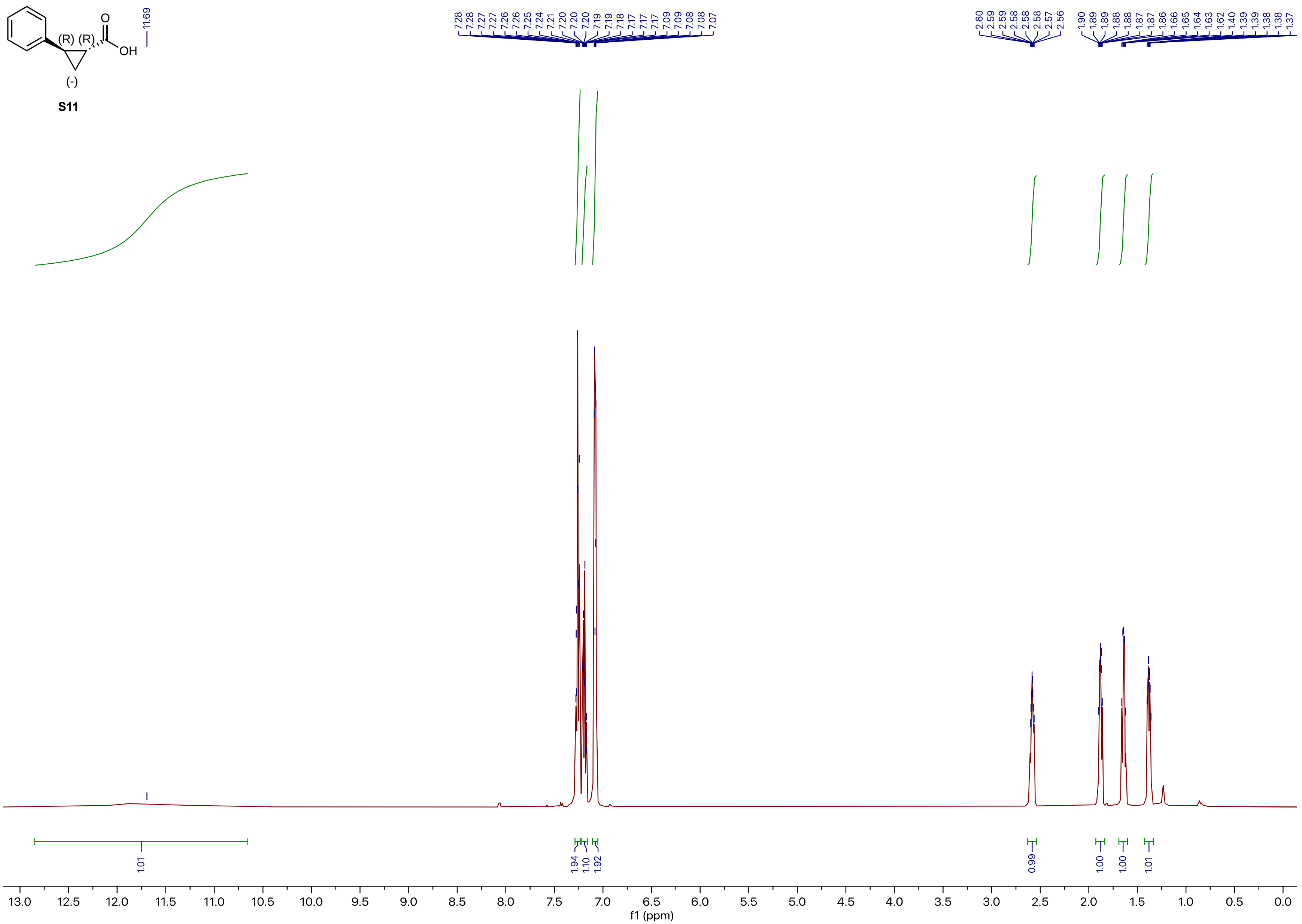

Supplementary Fig. 22 | ^{1}H-NMR (500 MHz, $CDCl_3$) of S11.

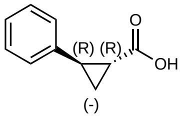

S11

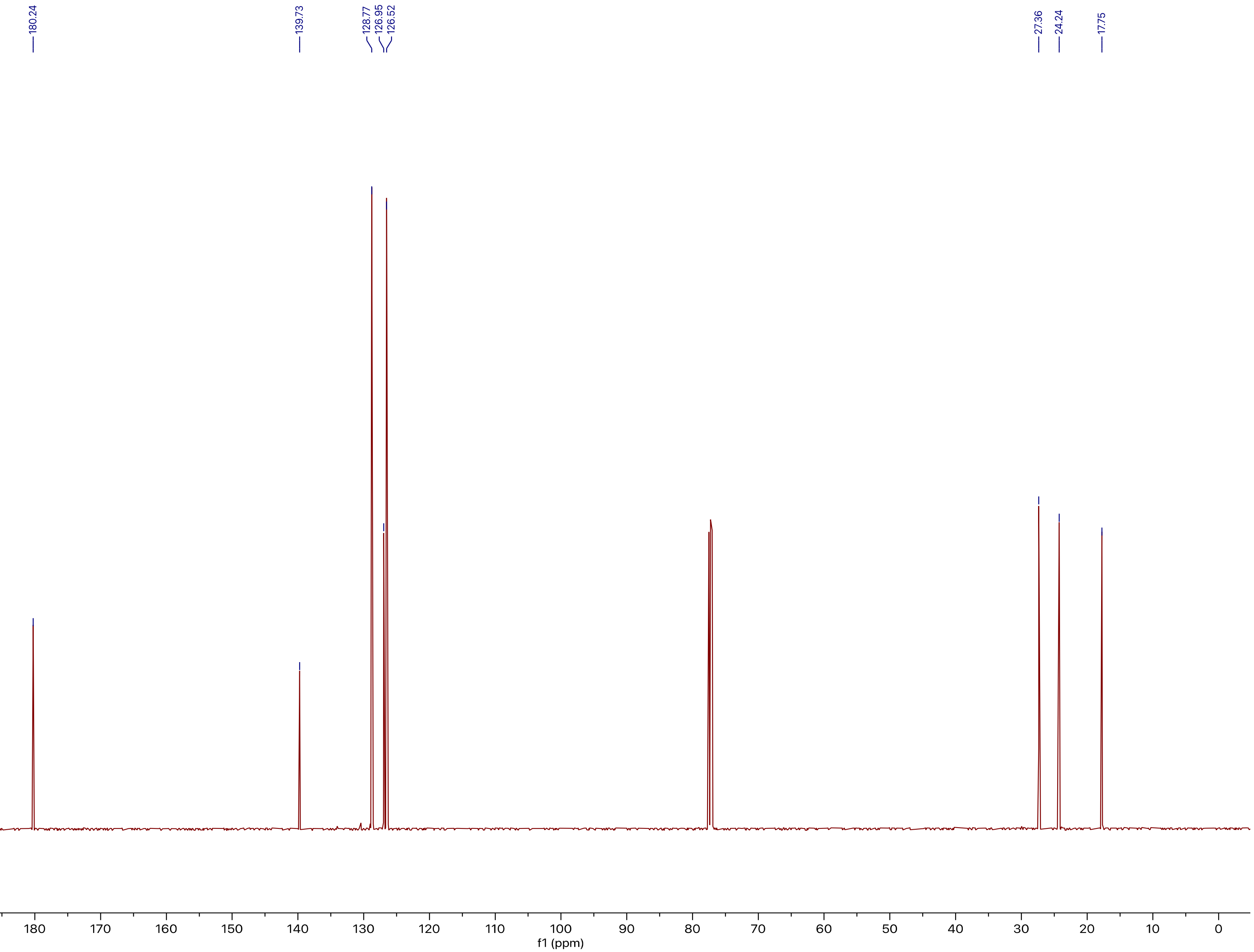

Supplementary Fig. 23 | ^{13}C-NMR (126 MHz, $CDCl_3$) of S11.

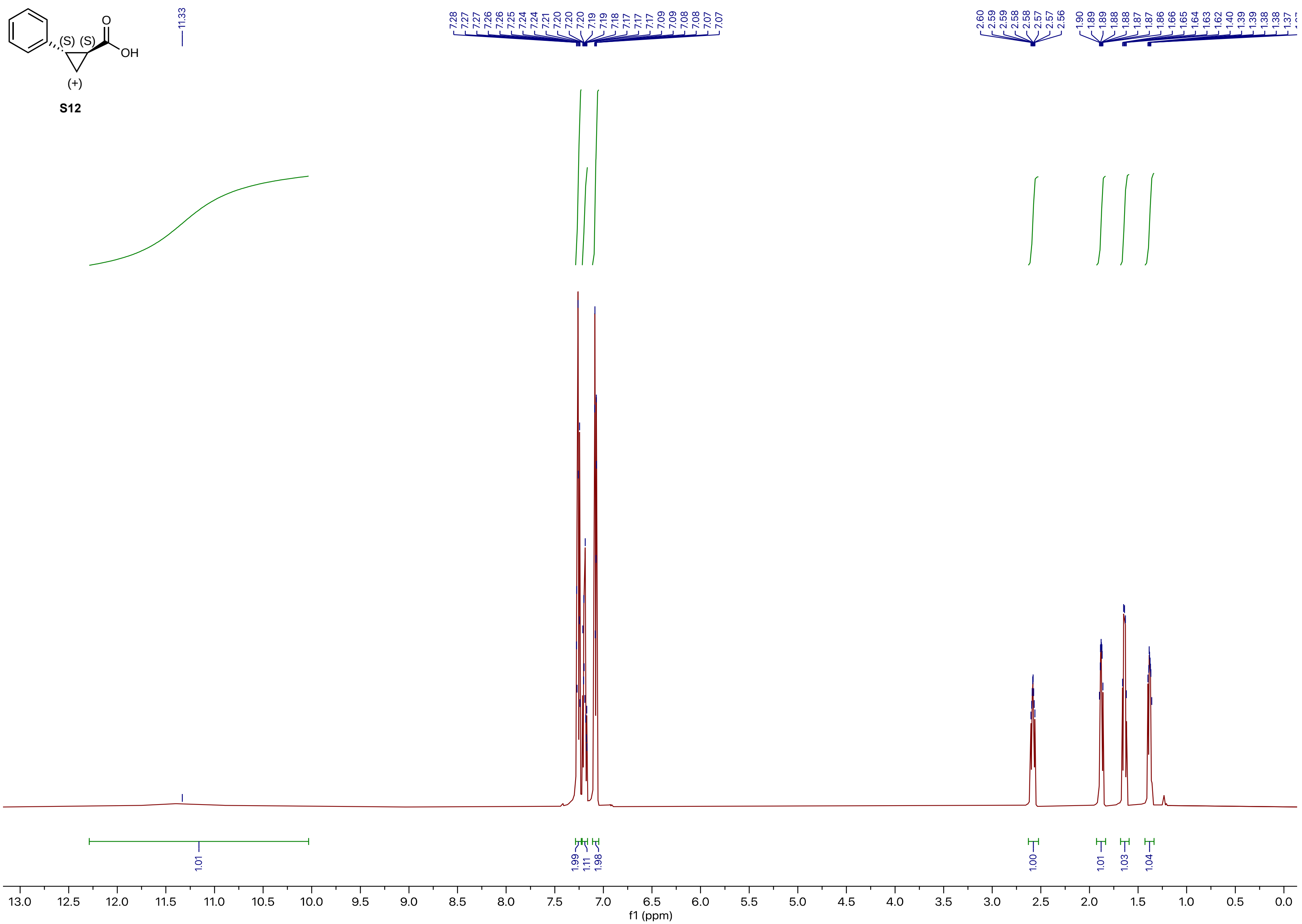

Supplementary Fig. 24 | ^{1}H-NMR (500 MHz, $CDCl_3$) of S12.

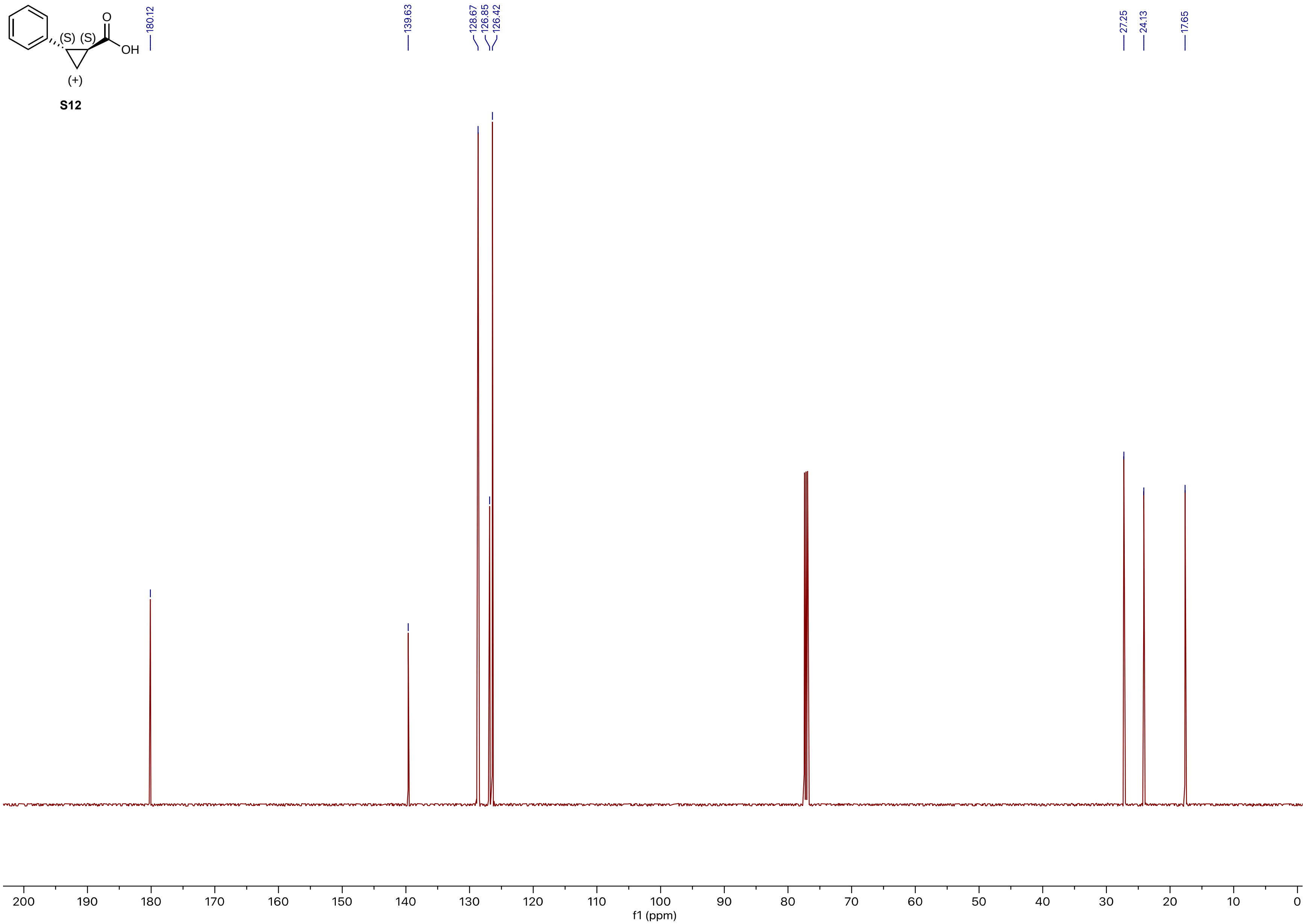

Supplementary Fig. 25 | ^{13}C-NMR (126 MHz, $CDCl_3$) of S12.

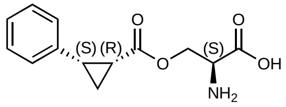

1

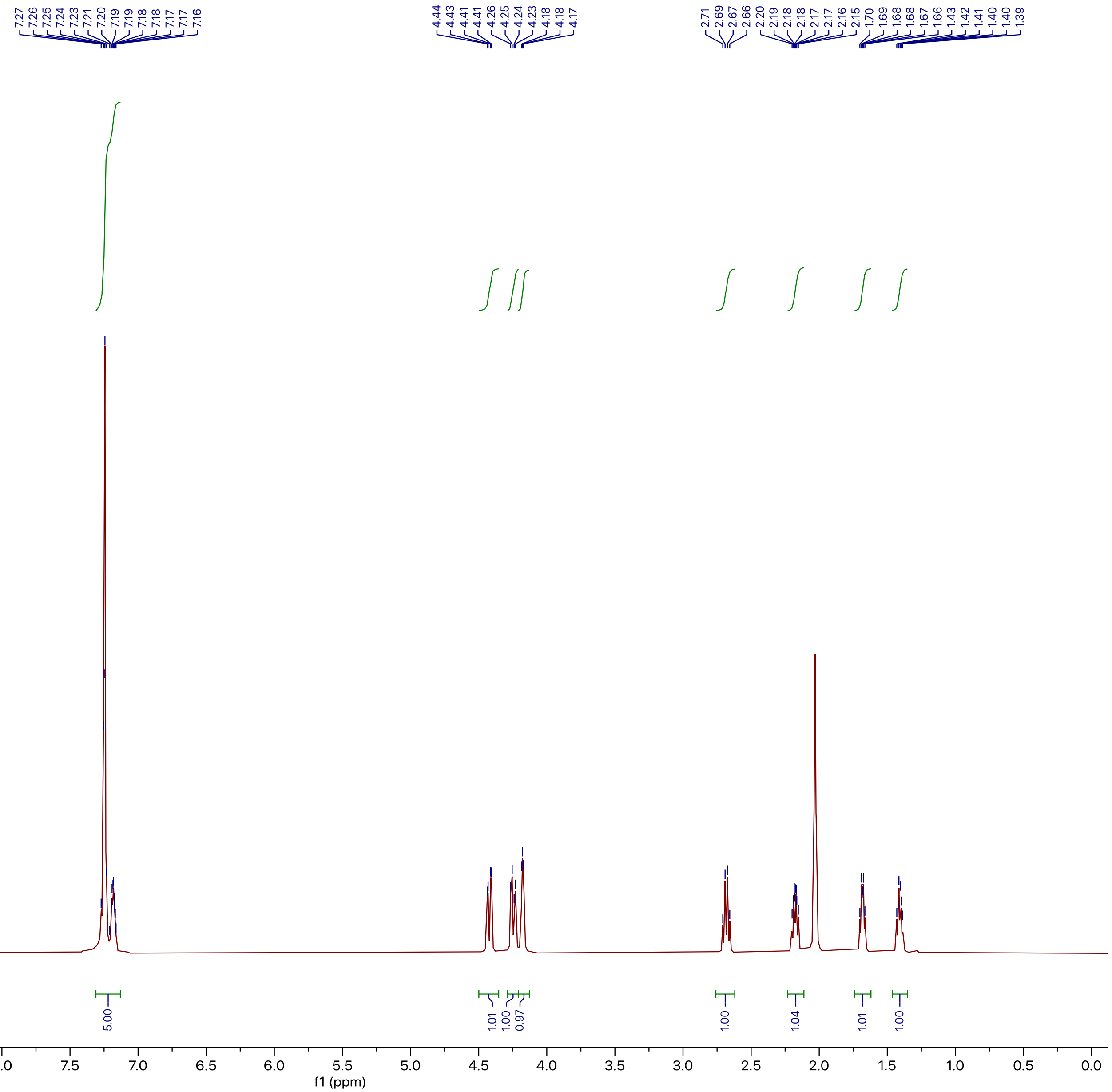

Supplementary Fig. 26 | ^{1}H-NMR (500 MHz, d_4-acetic acid) of 1.

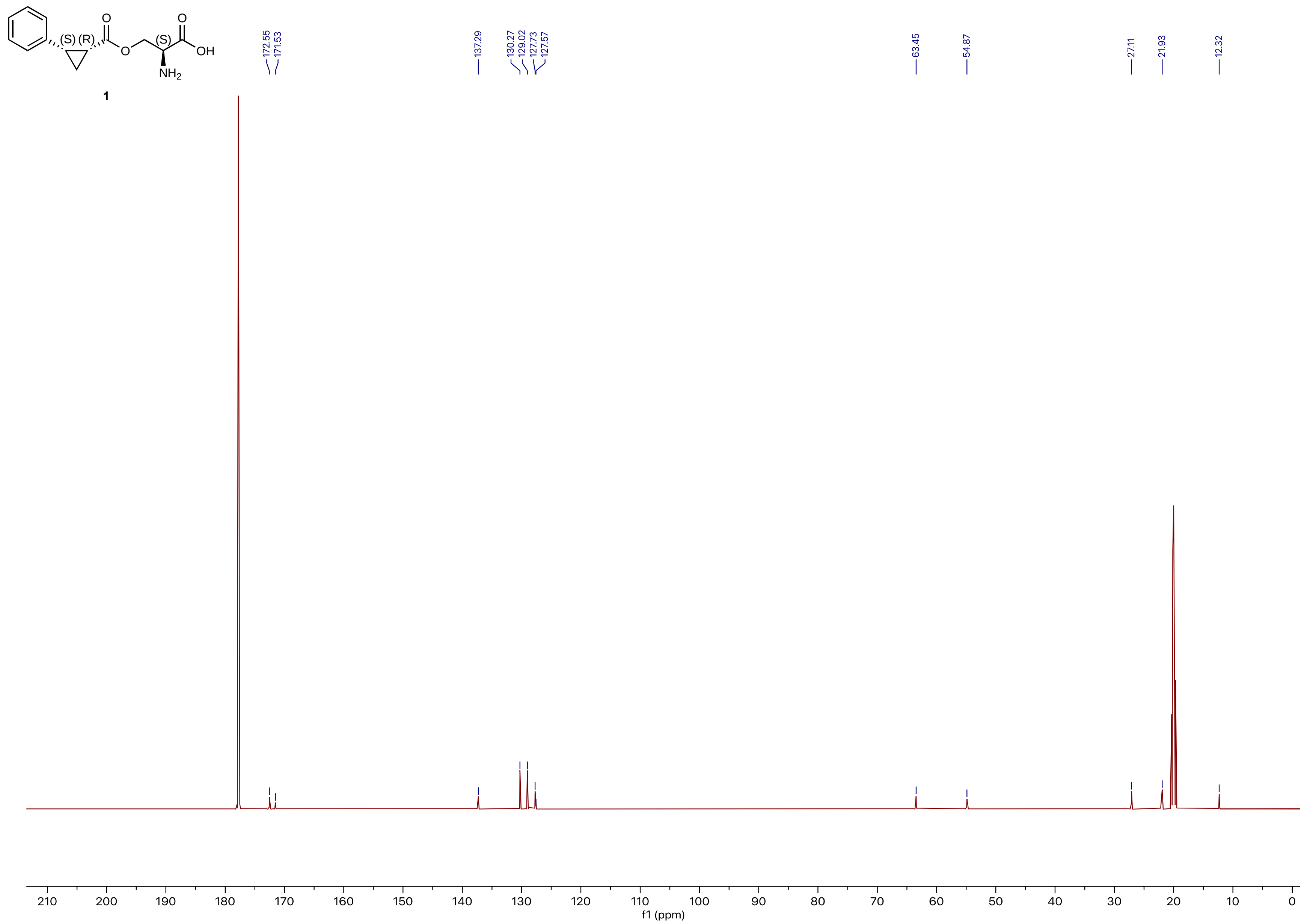

Supplementary Fig. 27 | ^{13}C-NMR (126 MHz, d_4-acetic acid) of 1.

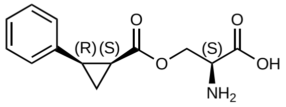

2

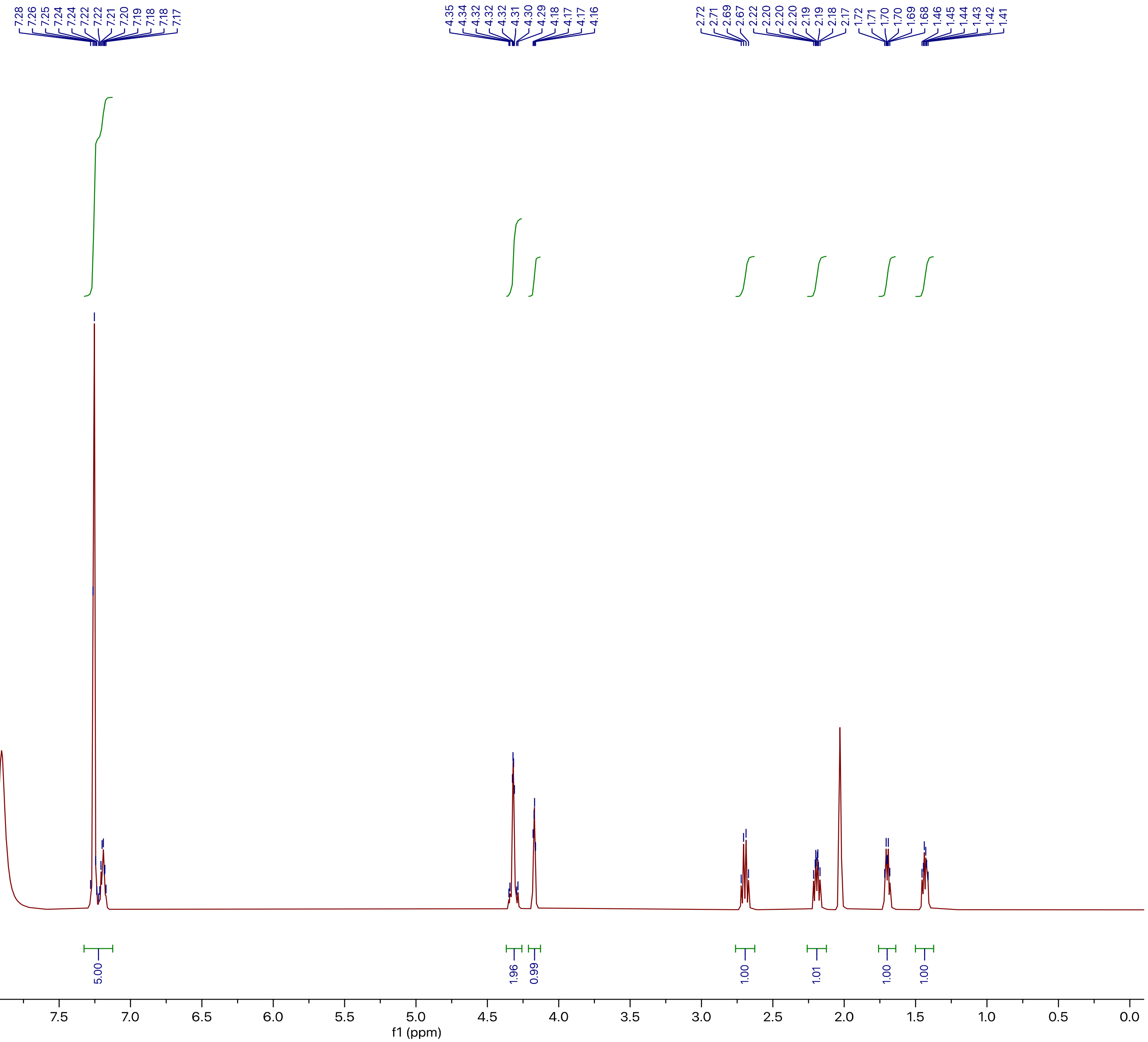

Supplementary Fig. 28 | ^{1}H-NMR (500 MHz, d_4-acetic acid) of 2.

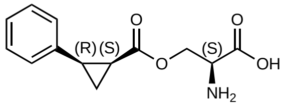

2

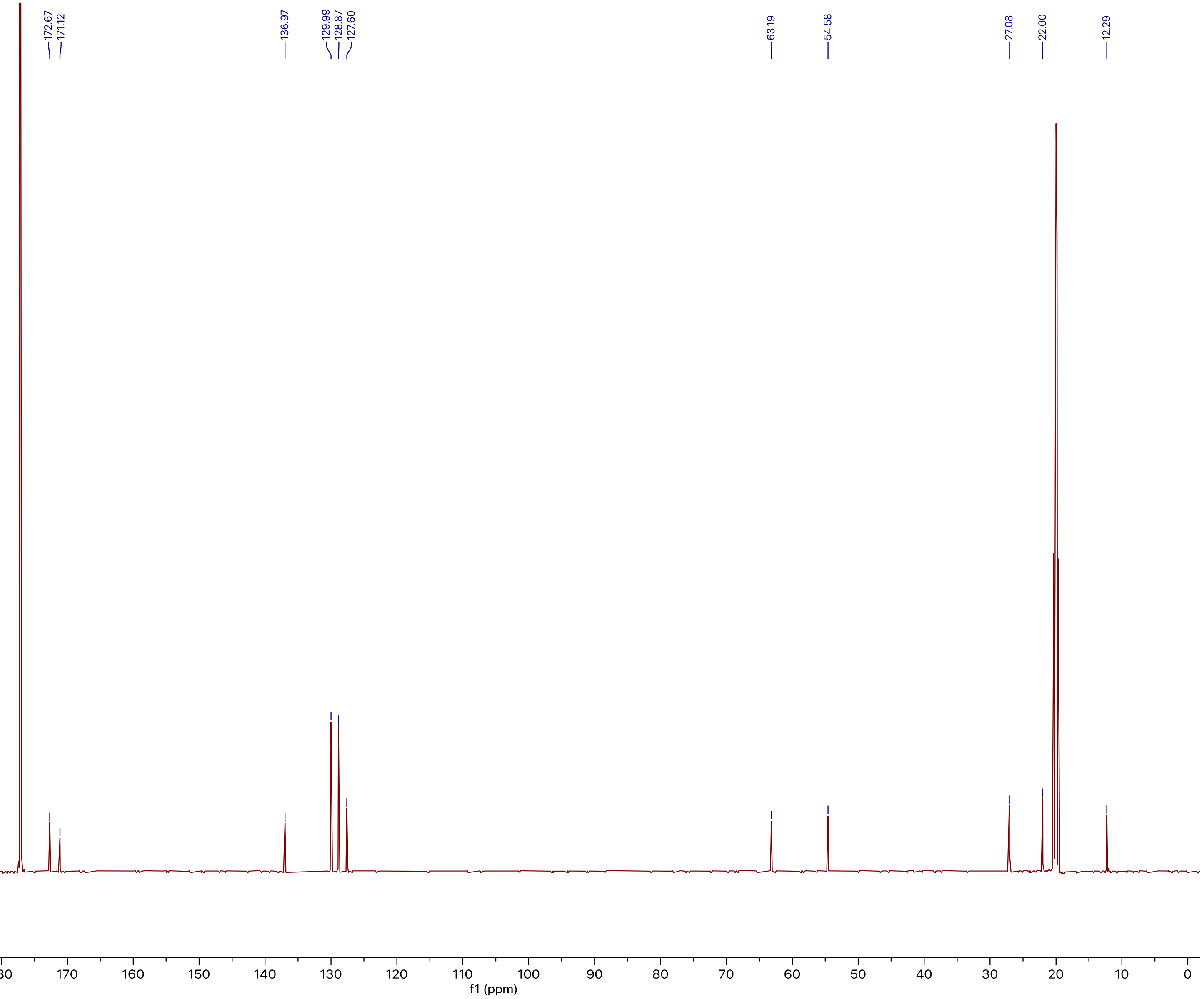

Supplementary Fig. 29 | ^{13}C-NMR (126 MHz, $CDCl_3$) of 2.

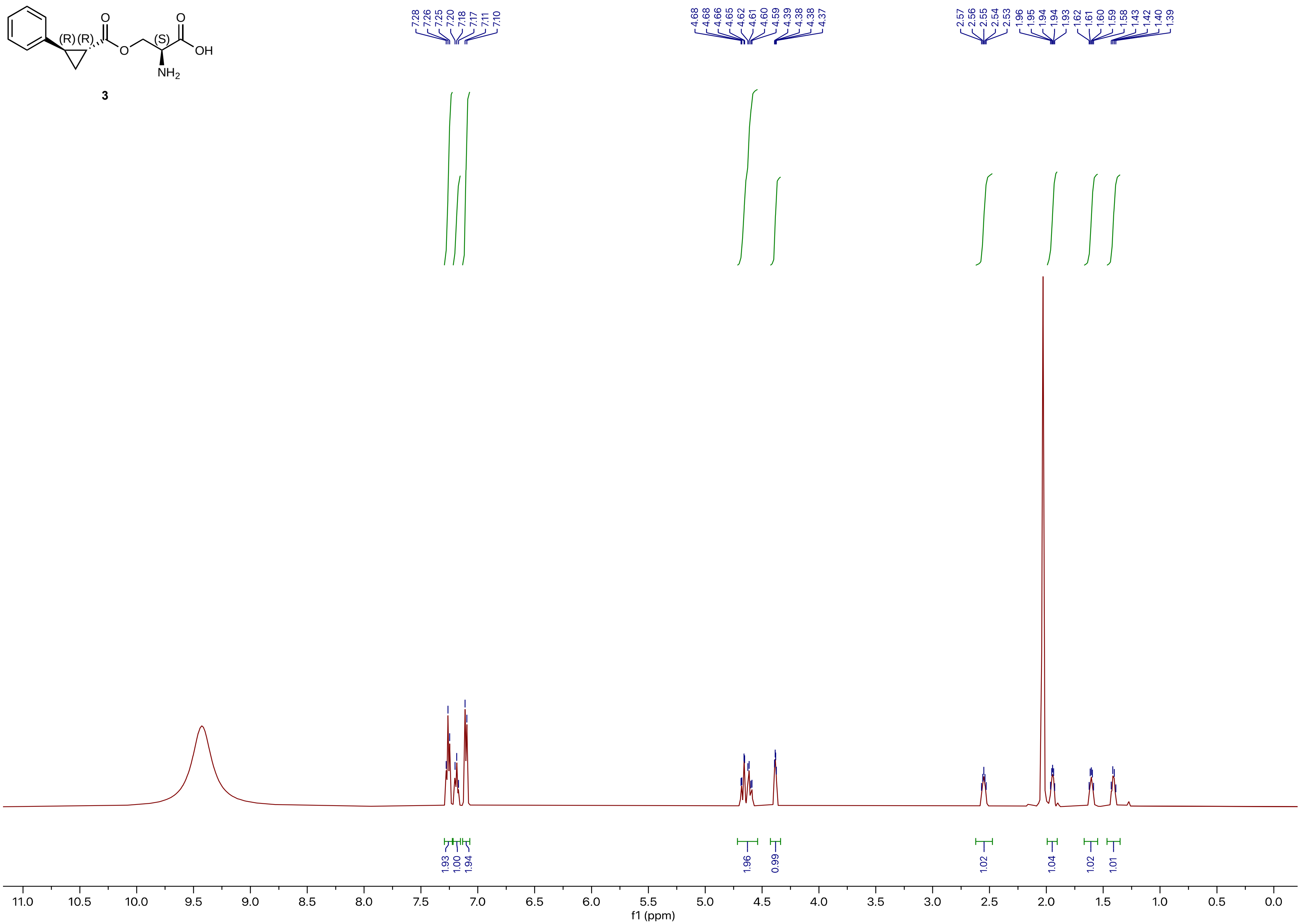

Supplementary Fig. 30 | ^{1}H-NMR (500 MHz, d_4-acetic acid) of 3.

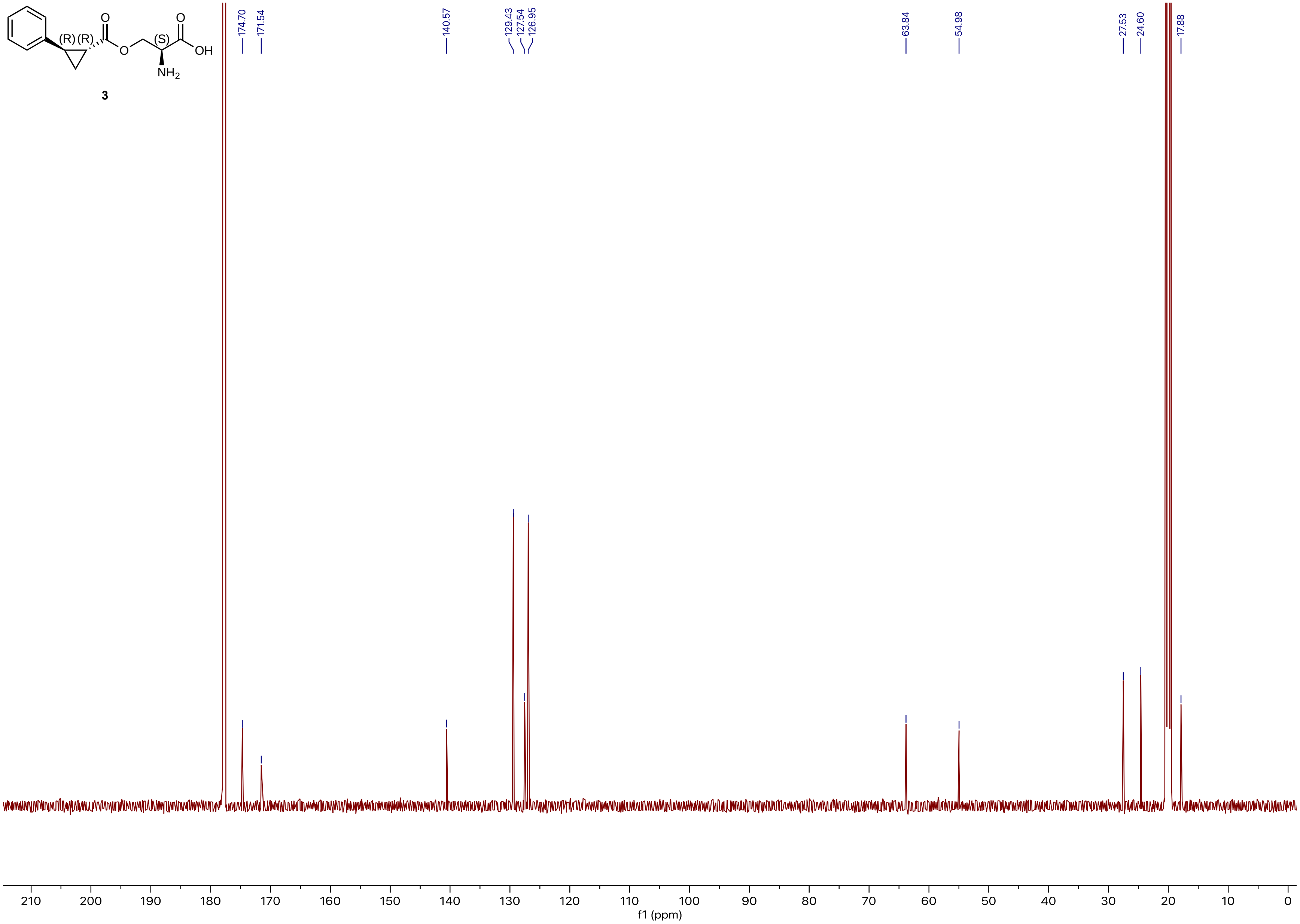

Supplementary Fig. 31 | ^{13}C-NMR (126 MHz, d$_4$-acetic acid) of 3.

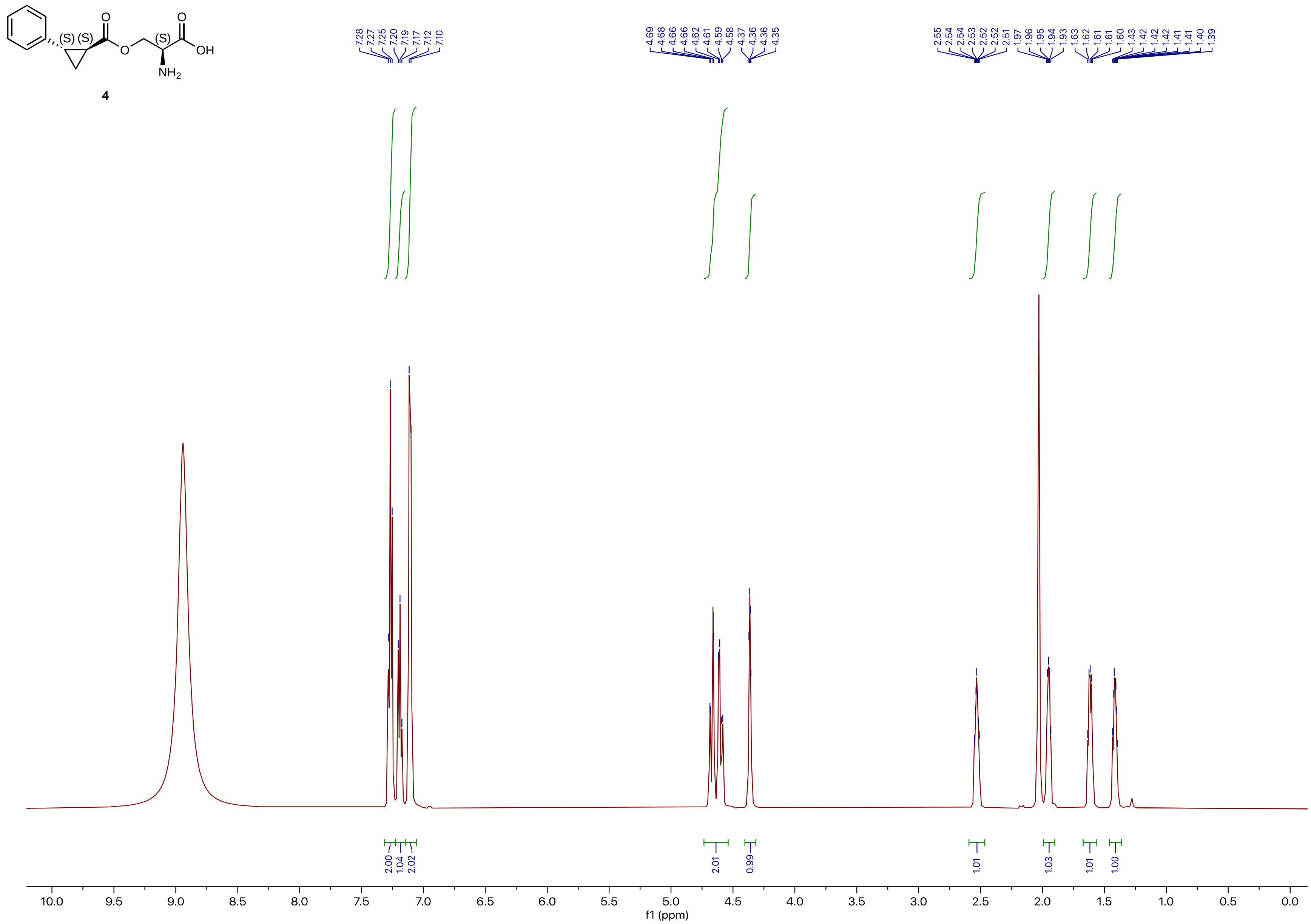

Supplementary Fig. 32 | ^{1}H-NMR (500 MHz, d4-acetic acid) of 4.

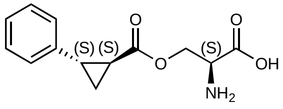

4

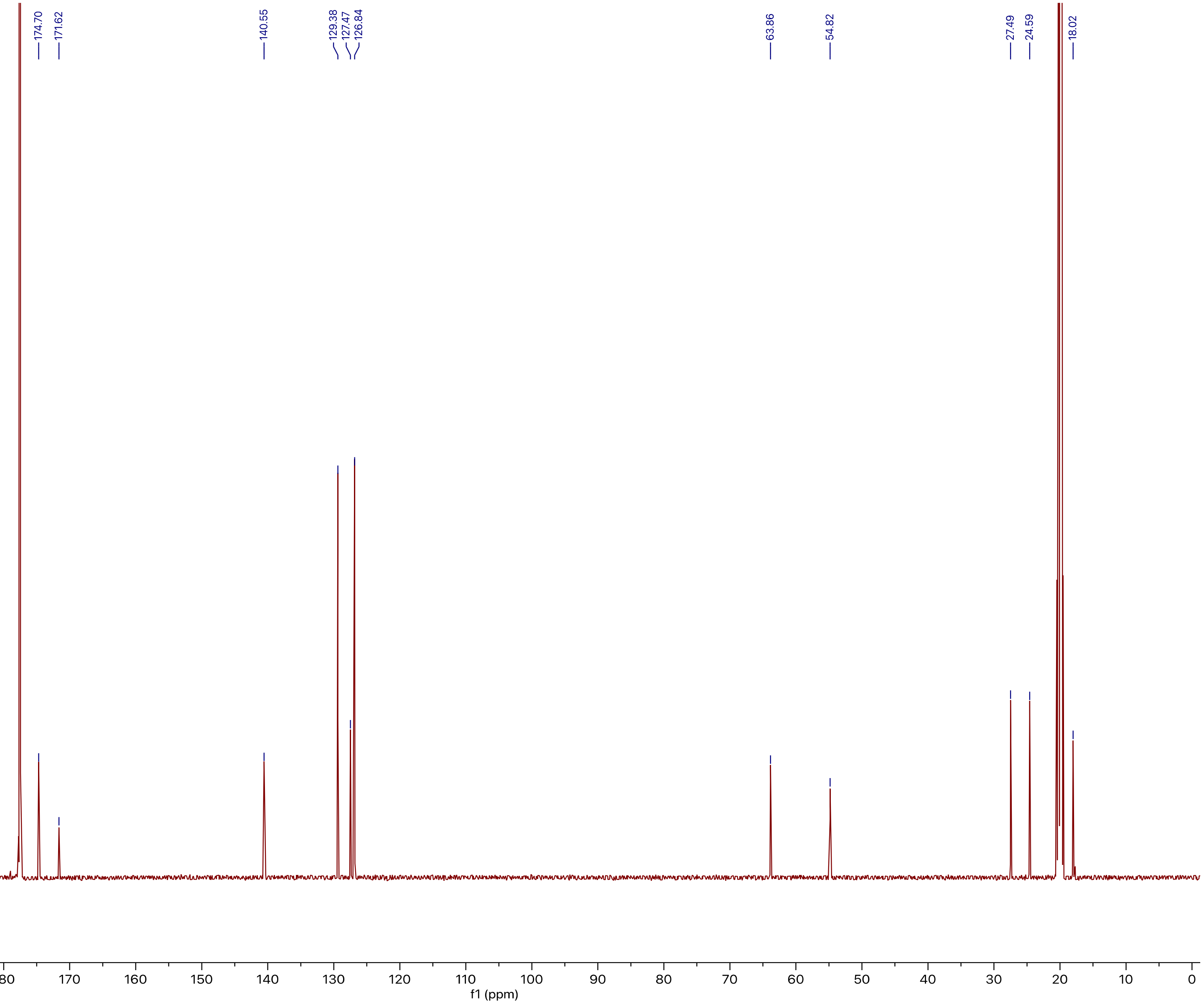

Supplementary Fig. 33 | ^{13}C-NMR (126 MHz, d$_4$-acetic acid) of 4.

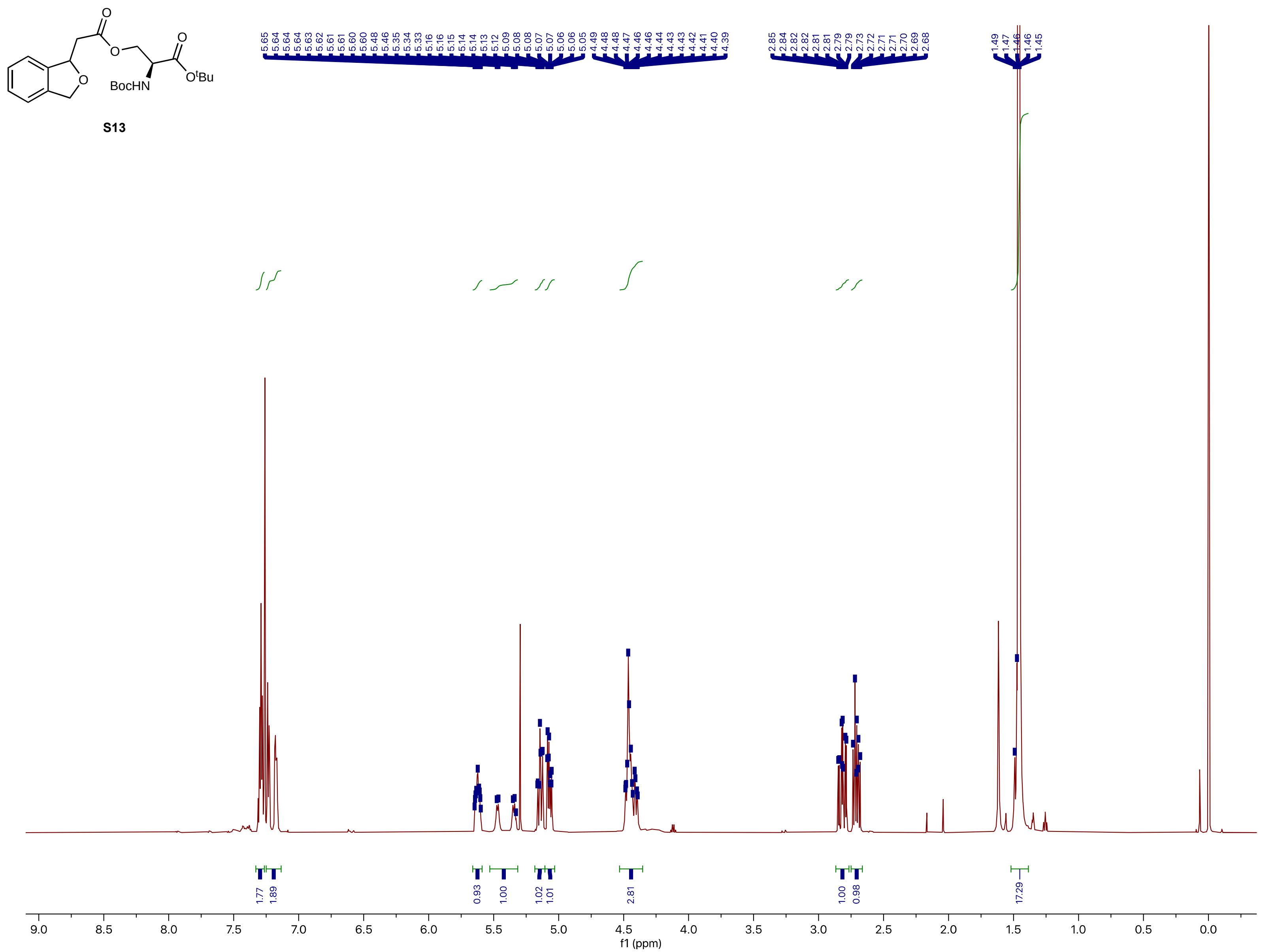

Supplementary Fig. 34 | ^{1}H-NMR (600 MHz, $CDCl_3$) of S13.

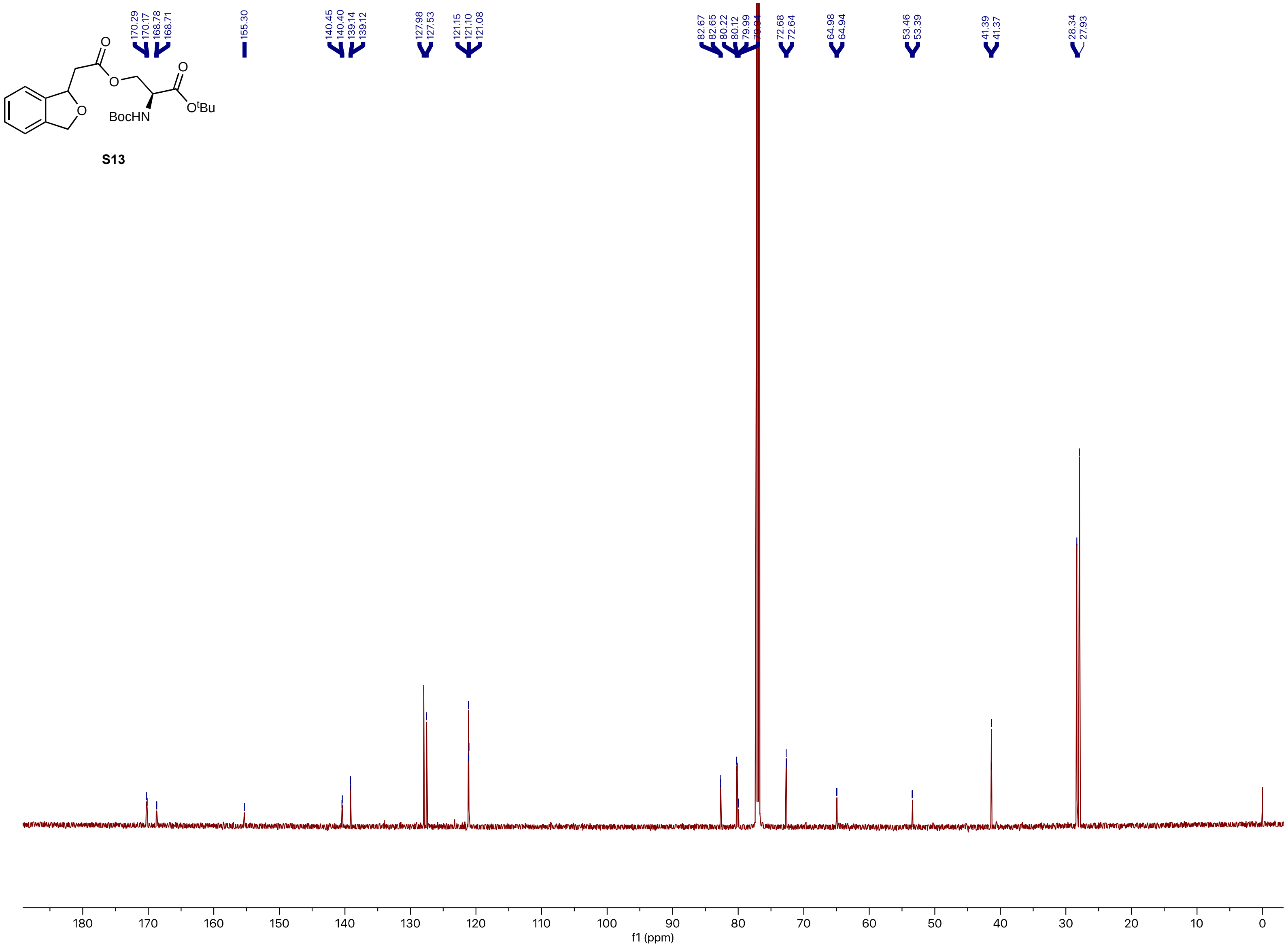

Supplementary Fig. 35 | ^{13}C-NMR (151 MHz, $CDCl_3$) of S13.

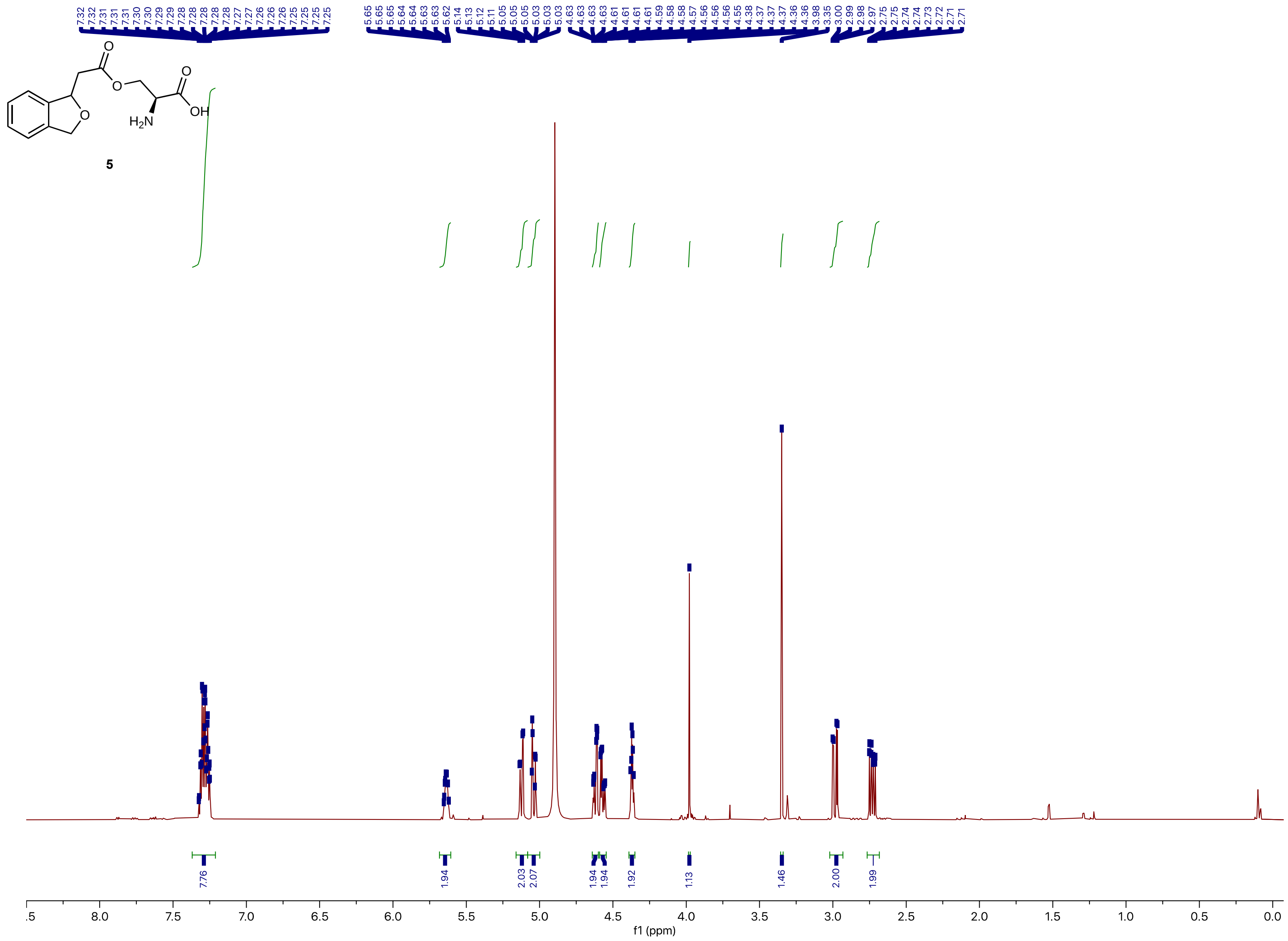

Supplementary Fig. 36 | ^{1}H-NMR (600 MHz, CD_3OD) of 5.

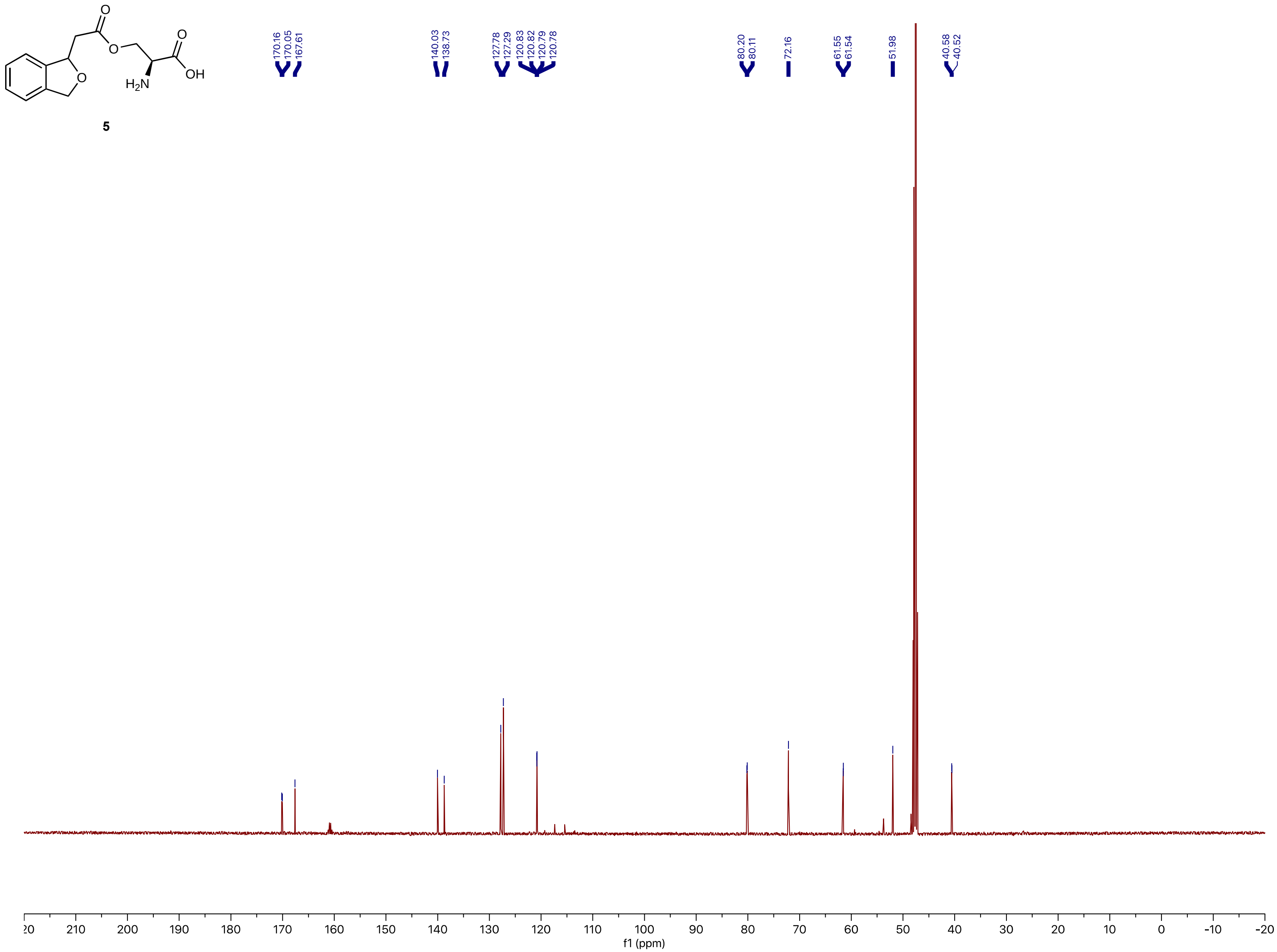

Supplementary Fig. 37 | ^{13}C-NMR (151 MHz, CD_3OD) of 5.

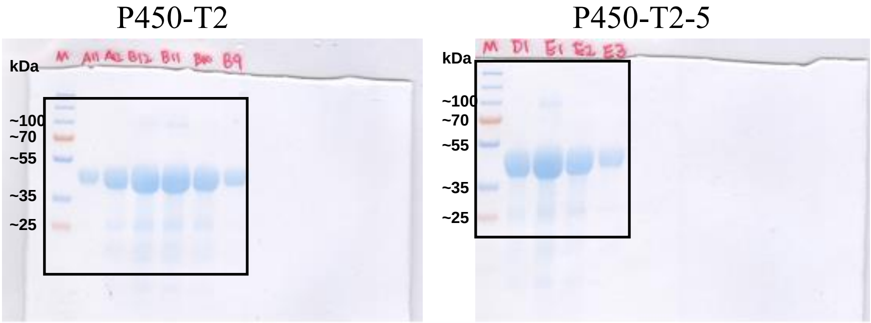

Supplementary Fig. 38 | Raw gel image for Extended Data Fig. 5a. Region in black rectangle was displayed.

Supplementary References

65 Gober, J. G. *et al.* P450-mediated non-natural cyclopropanation of dehydroalanine-containing thiopeptides. *ACS Chem. Biol.* **12**, 1726-1731 (2017).

66 Nguyen, K. T. *et al.* Characterization of a thermophilic cytochrome P450 of the CYP203A subfamily from Binh Chau hot spring in Vietnam. *FEBS Open Bio* **11**, 124-132 (2021).

67 Huang, J. *et al.* Unnatural biosynthesis by an engineered microorganism with heterologously expressed natural enzymes and an artificial metalloenzyme. *Nat. Chem.* **13**, 1186-1191 (2021).

68 Wang, Z. J. *et al.* Improved cyclopropanation activity of histidine-ligated cytochrome P450 enables the enantioselective formal synthesis of levomilnacipran. *Angew. Chem. Int. Ed.* **53**, 6810-6813 (2014).

69 Li, Y., Huang, J. S., Zhou, Z. Y., Che, C. M. & You, X. Z. Remarkably stable iron porphyrins bearing nonheteroatom-stabilized carbene or (alkoxycarbonyl) carbenes: isolation, X-ray crystal structures, and carbon atom transfer reactions with hydrocarbons. *J. Am. Chem. Soc.* **124**, 13185-13193 (2002).

70 Rosenberg, M. L., Vlasana, K., Sen Gupta, N., Wragg, D. & Tilset, M. Highly cis-selective Rh(I)-catalyzed cyclopropanation reactions. *J. Org. Chem.* **76**, 2465-2470 (2011).

71 Concellon, J. M., Rodriguez-Solla, H. & Simal, C. The first cyclopropanation reaction of unmasked alpha,beta-unsaturated carboxylic acids: direct and complete stereospecific synthesis of cyclopropanecarboxylic acids promoted by Sm/CHI3. *Org. Lett.* **9**, 2685-2688 (2007).

72 Evans, D. A., Britton, T. C. & Ellman, J. A. Contrasteric carboximide hydrolysis with lithium hydroperoxide. *Tetrahedron Lett.* **28**, 6141-6144 (1987).

73 Cheng, K. J. *et al.* Probes for narcotic receptor mediated phenomena. 41. Unusual inverse mu-agonists and potent mu-opioid antagonists by modification of the N-substituent in enantiomeric 5-(3-hydroxyphenyl)morphans. *J. Med. Chem.* **54**, 957-969 (2011).
